# Simple Expression Domains Are Regulated by Discrete CRMs During *Drosophila* Oogenesis

**DOI:** 10.1534/g3.117.043810

**Published:** 2017-06-20

**Authors:** Nicole T. Revaitis, Robert A. Marmion, Maira Farhat, Vesile Ekiz, Wei Wang, Nir Yakoby

**Affiliations:** * Center for Computational and Integrative Biology, Rutgers, The State University of New Jersey, Camden, New Jersey 08103; † Department of Biology, Rutgers, The State University of New Jersey, Camden, New Jersey 08103; ‡ Lewis-Sigler Institute for Integrative Genomics, Princeton University, New Jersey 08544

**Keywords:** gene regulation, eggshell patterning, GAL4, genetic tools

## Abstract

Eggshell patterning has been extensively studied in *Drosophila melanogaster*. However, the *cis*-regulatory modules (CRMs), which control spatiotemporal expression of these patterns, are vastly unexplored. The FlyLight collection contains >7000 intergenic and intronic DNA fragments that, if containing CRMs, can drive the transcription factor GAL4. We cross-listed the 84 genes known to be expressed during *D. melanogaster* oogenesis with the ∼1200 listed genes of the FlyLight collection, and found 22 common genes that are represented by 281 FlyLight fly lines. Of these lines, 54 show expression patterns during oogenesis when crossed to an UAS-GFP reporter. Of the 54 lines, 16 recapitulate the full or partial pattern of the associated gene pattern. Interestingly, while the average DNA fragment size is ∼3 kb in length, the vast majority of fragments show one type of spatiotemporal pattern in oogenesis. Mapping the distribution of all 54 lines, we found a significant enrichment of CRMs in the first intron of the associated genes’ model. In addition, we demonstrate the use of different anteriorly active FlyLight lines as tools to disrupt eggshell patterning in a targeted manner. Our screen provides further evidence that complex gene patterns are assembled combinatorially by different CRMs controlling the expression of genes in simple domains.

The spatiotemporal control of gene expression is a fundamental requirement for animal development (Levine 2010; Davidson and Erwin 2006). Research in *Drosophila melanogaster* has provided insight into the complex process of tissue patterning and cell-fate determination during animal development (*e.g*., Konikoff *et al.* 2012; Lecuyer *et al.* 2007; Tomancak *et al.* 2002). Large-scale screens for *cis*-regulatory modules (CRMs), which control spatiotemporal expression of genes, provided compelling examples of gene patterning in embryo, central nervous system (CNS), and imaginal disc development (Jenett *et al.* 2012; Manning *et al.* 2012; Pfeiffer *et al.* 2008; Jory *et al.* 2012; Li *et al.* 2014). Despite comprehensive screens to systematically search for CRMs in *Drosophila*, our understanding of how genes are regulated in time and space is still limited (Manning *et al.* 2012; D. C. Arnold *et al.* 2013; Kvon *et al.* 2014; Pfeiffer *et al.* 2008). Furthermore, analysis of gene regulation during *Drosophila* oogenesis still remains underexplored.

The *D. melanogaster* eggshell is an established experimental system to study the patterning of the 2D epithelial tissue that forms the intricate 3D structures of the eggshell (*e.g*., Wasserman and Freeman 1998; Berg 2005; Neuman-Silberberg and Schüpbach 1993; Hinton 1981; Horne-Badovinac and Bilder 2005; Niepielko *et al.* 2011; Osterfield *et al.* 2015; Peri and Roth 2000; Twombly *et al.* 1996; Yakoby *et al.* 2008b; Zartman *et al.* 2008). Studies have focused on the role of cell signaling pathways in follicle cell patterning and eggshell morphogenesis (Lembong *et al.* 2009; Marmion *et al.* 2013; Neuman-Silberberg and Schüpbach 1993; Niepielko *et al.* 2012; Peri and Roth 2000; Queenan *et al.* 1997; Sapir *et al.* 1998; Schnorr *et al.* 2001; Wasserman and Freeman 1998; Zartman *et al.* 2011). Numerous studies demonstrated that gene expression is dynamic and diverse during oogenesis of *D. melanogaster* and other *Drosophila* species (Kagesawa *et al.* 2008; Nakamura and Matsuno 2003; Berg 2005; Jordan *et al.* 2005; Niepielko *et al.* 2011, 2014; Yakoby *et al.* 2008a; Zartman *et al.* 2009b). While these studies gathered substantial information on the patterning dynamics of genes, the analysis of active CRMs during oogenesis is restricted to a handful of genes (Andrenacci *et al.* 2000; Marmion *et al.* 2013; Fuchs *et al.* 2012; Cheung *et al.* 2013; Charbonnier *et al.* 2015; Tolias *et al.* 1993; Cavaliere *et al.* 1997; Andreu *et al.* 2012).

Tolias and colleagues demonstrated that a seemingly uniform expression in the follicle cells is actually regulated by distinct spatial and temporal elements (Tolias *et al.* 1993). Motivated by this and the prediction that complex patterns of genes are comprised of simple expression domains (Yakoby *et al.* 2008a), we used the FlyLight collection of flies to search for oogenesis-related CRMs. FlyLight lines, which were initially selected for those genes that showed expression in the adult brain (Jenett *et al.* 2012), contain the transcription factor GAL4 downstream of the DNA fragments. We crossed 281 FlyLight lines, which represent 22 of the 84 genes known to be expressed during oogenesis, to a UAS-GFP. We found 54 lines positive for GFP. In 30% of these lines, the full or partial pattern of the associated endogenous pattern was recapitulated. In addition, we found that CRM distribution is significantly enriched in the first intron of the gene locus model. Finally, we demonstrated the use of several fly lines as a tool to perturb eggshell patterning.

## Materials and Methods

### Fly stocks

The FlyLight lines (Pfeiffer *et al.* 2008, 2010) were obtained through the Bloomington *Drosophila* Stock Center, Indiana University. All tested FlyLight stocks are listed in Supplemental Material, Figure S1. FlyLight lines (males) were crossed to P[UAS-Stinger]GFP:NLS (Barolo *et al.* 2000) virgin females. To overcome lethality associated with genetic perturbations (see below), FlyLight lines were first crossed to a temperature-sensitive GAL80, P[tubP-GAL80^ts^]10 (Bloomington ID# 7108). The *dad-lacZ* and *dpp-lacZ* reporters (see below) were crossed to E4-GAL4 (Queenan *et al.* 1997) and a UAS-*dpp* (a gift from Trudi Schüpbach). EGFR signaling was upregulated by a UAS-λtop-4.2 [caEGFR (Queenan *et al.* 1997)] and downregulated by a UAS-dnEGFR (a gift from Alan Michelson). Progeny were heat shocked at 28° for 3 d to alleviate repression by GAL80^ts^. Flies were grown on cornmeal agar at 23°.

The *dad-lacZ* and *dpp-lacZ* reporters were constructed based on the *dad*^44C10^ and *dpp*^18E05^ DNA fragments. The coordinates for these DNA fragments were taken from http://flweb.janelia.org/cgi-bin/flew.cgi. These fragments were amplified from OreR using phusion polymerase (NEB), A-tailed with Taq, and cloned into a PCR8/GW/TOPO vector by TOPO cloning (Invitrogen, Carlsbad, CA). The fragments were then Gateway cloned into a pattBGWhZn (Marmion *et al.* 2013). Both reporter constructs were injected into the attP2 line (Stock# R8622, Rainbow Transgenic Flies) and integrated into the 68A4 chromosomal position by PhiC31/attB-mediated integration (Groth *et al.* 2004).

### Immunofluorescence and microscopy

Immunoassays were performed as previously described (Yakoby *et al.* 2008b). In short, flies 3- to 7-d old were put on yeast and dissected in ice cold Grace’s insect medium, fixed in 4% paraformaldehyde, washed three times, permeabilized (PBS and 1% Triton X-100), and blocked for 1 hr (PBS, 0.2% Triton X-100, and 1% BSA). Ovaries were then incubated overnight at 4° with primary antibody. After washing three times with PBST (0.2% Triton X-100), ovaries were incubated in secondary antibodies for 1 hr at 23°. Then, ovaries were washed three times and mounted in Flouromount-G (Southern Biotech). Primary antibodies used were sheep anti-GFP (1:5000; Serotec), rabbit anti-β-galactosidase (1:1000; Invitrogen) (Yakoby *et al.* 2008b), mouse anti-Broad (anti-BR) (1:400; stock #25E9.D7, Hybridoma Bank), and rabbit anti-phosphorylated-Smad1/5/8 (1:3600; a gift from D. Vasiliauskas, S. Morton, T. Jessell, and E. Laufer) (Yakoby *et al.* 2008b). Secondary antibodies used were Alexa Fluor 488 (anti-mouse), Alexa Fluor 488 (anti-sheep), Alexa Fluor 568 (anti-mouse), and Alexa Fluor 568 (anti-rabbit) (1:2000; Molecular Probes) for the screen, perturbations, and β-galactosidase staining. Nuclear staining was performed using DAPI (84 ng/ml). The pattern of BR was used as a spatial reference to characterize the dorsal side of the egg chamber. Two ovaries from an internal positive control, *rho*^38A01^, were added in each immunoassay due to the unique expression pattern in the border cells (Figure S2Tc). Unless specified differently, all immunoflourescent images were captured with a Leica SP8 confocal microscope (confocal Core Facility, Rutgers University Camden). For SEM imaging, eggshells were mounted on double-sided carbon tape and sputter coated with gold palladium for 60 sec. Images were taken using a LEO 1450EP.

### RNA-sequencing analysis

The specific isoform expressed during oogenesis for each of the 22 genes was identified using RNA-sequencing (RNA-seq) analysis (Figure S2). Egg chambers were analyzed at three developmental stages: (1) egg chambers at stages 9 or earlier, (2) egg chambers at stages 10A and 10B, (3) egg chambers at stages S11 or greater. Isolation of egg chambers was done manually as previously described (Yakoby *et al.* 2008a). All RNA samples (∼200 egg chambers from each developmental group) were extracted using RNeasy Mini Kit (QIAGEN, Valencia, CA). 1 μg of total RNA from each sample was subjected to poly-A-containing RNA enrichment by oligo-dT beads and then converted to the RNA-seq library using the automated Apollo 324 NGS Library Prep System and associated kits (WaferGen, Fremont, CA), according to the manufacturer’s protocol, using different DNA barcodes in each library. The libraries were examined on Bioanalyzer (Agilent, Santa Clara, CA) DNA HS chips for size distribution, and quantified by the Qubit Fluorometer (Invitrogen). The set of three RNA-seq libraries was pooled together at equal amounts and sequenced on Illumina HiSequation 2500 in rapid mode as one lane of single-end, 65-nt reads following the standard protocol. Raw sequencing reads were filtered by Illumina HiSeq Control Software and only the pass-filter (PF) reads were used for further analysis. PF reads were demultiplexed using the Barcode Splitter in FASTX-Toolkit. Then the reads from each sample were mapped to the dm3 reference genome with gene annotation from FlyBase using TopHat 1.5.0 software. Expression level was further summarized at the gene level using htseq-count 0.3 software, including only the uniquely mapped reads. Data were viewed using IGV software (Thorvaldsdottir *et al.* 2013; Robinson *et al.* 2011). The RNA-seq alignments show the coverage plot of each of the screened genes aligned to the reference genome gene track(s) (Figure 4 and Figure S2, Aa–Va). The peaks in the coverage plot represent the number of reads per base pair. The color code represents miscalls or SNPs to the reference genome. In these cases, red, blue, orange, and green represent cytosine, thymine, guanine, and adenosine mismatches, respectively; and gray represents a match. The RNA-seq data are available here: http://dx.doi.org/doi:10.7282/T3ZS300V.

### Statistical analysis of DNA fragments distribution

Fragments of DNA were divided into three bins: those upstream of the transcription start site (TSS) were categorized as *Proximal*, fragments within the first intron were categorized as *Intron 1*, and those downstream of the second exon were categorized as *Distal*. The TSS was assigned by selecting the longest isoform expressed, which was extrapolated from the RNA-seq data, unless otherwise noted from references in the literature. Introns <300 bp were not included in the FlyLight collection (Pfeiffer *et al.* 2008). Statistical analysis was performed using a χ^2^ test (*n* = 3, thus d.f. = 2). The null hypothesis is that the frequency of the CRMs among categories is identical. Pairwise testing for enrichment of specific categories was determined using a one-tailed binomial test, where the number of expected GFP-expressing lines was 22%, and the size for each of the categories was 140, 72, and 69 for *Proximal*, *Intron 1*, and *Distal*, respectively. The observed numbers of GFP-expressing fragments were 23, 22, and 9 for *Proximal*, *Intron 1*, and *Distal*, respectively. The *P*-values were calculated in MatLab using the myBinomTest (s,n,p,Sided) script available at http://www.MathWorks.com.

### Pattern annotation and matrix formation

**Figure 1:**
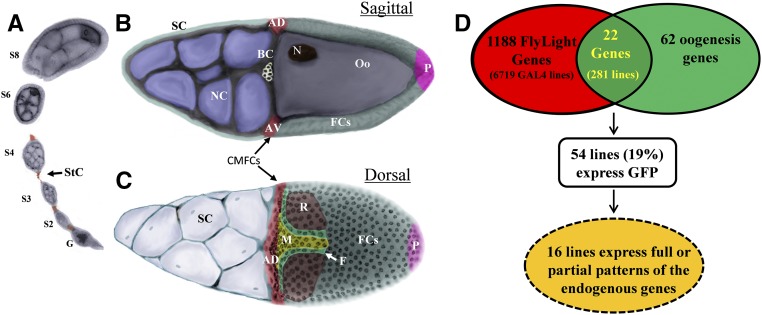
Screening for expression domains during oogenesis. (A) Early stages of egg chamber (stages 2–8). Germarium (G) and stalk cells (StC). Egg chamber at stage 10B in (B) sagittal and (C) dorsal views. Different groups/domains of cells are marked, including border cells (BC), stretched cells (SC), nurse cells (NC), centripetally migrating follicle cells (CMFCs), oocyte-associated follicle cells (FCs), oocyte (Oo), and oocyte nucleus (N). Cellular domains are marked, including the anterior (A), posterior (P), midline (M), floor (F), and roof (R). (D) Summary of the screen for CRMs during oogenesis.

We adopted the previously developed annotation system for patterning of follicle cells (Niepielko *et al.* 2014; Yakoby *et al.* 2008a). Briefly, the annotation of gene patterning is based on simple domains, primitives, which repeat across different expression patterns. The assembly of primitives provides a tool for the description of complex gene expression patterns in the follicle cells. Each domain is coded into a binary matrix as 0 (no expression) or 1 (expression), which allows us to simply add new domains into the matrix. In our screen, in addition to different domains in the follicle cells, other domains were added, including stretched cells, border cells, polar cells, and the germarium (Figure 1, A–C). In addition, we added two new domains, the dorsal appendages and operculum, for stage 14, which are used for the calculations in Figure 5. These domains are presented in Figure S2. The annotations of the endogenous gene patterns and the patterns of GFP-positive FlyLight lines were performed by three independent researchers. Each pattern was annotated into an Excel spreadsheet using a binary system for each domain. The annotations for individual lines were collapsed to represent one input for that gene at stages where the endogenous pattern is detected. To determine the overlap between the FlyLight GFP expression pattern and the endogenous expression pattern of the gene, the matrix of GFP-positive domains was compared to the *in situ* hybridization matrix (the sources of these expression patterns are included in the captions of Figure S2). The overlay of matrices was done in MatLab using imagesc.

### Data availability

Flies are available in the Bloomington *Drosophila* Stock Center. Figure S1 contains the detailed description of all FlyLight lines used in this study. Figure S2 contains all GFP expression patterns of the study. All RNA-seq data are publicly available at http://dx.doi.org/doi:10.7282/T3ZS300V.

## Results

### Screening for regulatory domains

During oogenesis, the egg chamber, the precursor of the mature egg, is extensively patterned through 14 morphologically distinct stages (Figure 1, A–C) (Berg 2005; Yakoby *et al.* 2008a; Jordan *et al.* 2005; Spradling 1993). Previously, we characterized the expression pattern of >80 genes in the follicle cells, a layer of epithelial cells surrounding the oocyte (Niepielko *et al.* 2014; Yakoby *et al.* 2008a). We established that genes are expressed dynamically in distinct domains that can be combinatorially assembled into more complex patterns. Here, in addition to the follicle-cell expression domains, we also documented gene/reporter expression in additional domains, including the germarium (G), stalk cells (StC), border cells (BC), and stretched cells (SC) (Figure 1, A–C). These domains serve as a platform for the spatiotemporal patterning analysis.

To identify the CRMs of genes controlling tissue patterning, we took advantage of the FlyLight collection of flies, which consists of ∼7000 fly lines containing intronic and intergenic DNA fragments, representing potential regulatory regions of ∼1200 genes (Jenett *et al.* 2012; Pfeiffer *et al.* 2008). Our screen focused on the 84 genes known to pattern the follicle cells during oogenesis (*e.g.*, Yakoby *et al.* 2008a; Fregoso Lomas *et al.* 2013; Dequier *et al.* 2001; Jordan *et al.* 2005; Deng and Bownes 1997; Ruohola-Baker *et al.* 1993). Cross-listing the 84 genes with the FlyLight list yielded 22 common genes (Figure 1D). These genes are associated with a total of 281 fly lines containing CRMs that are potentially active during oogenesis. All DNA fragments in the FlyLight collection are upstream to the transcription factor GAL4. Crossing these lines to a UAS-pStinger-GFP fly yielded 54 GFP-positive lines. Of importance, 16 of the 54 fly lines recapitulated the full or partial endogenous pattern of the corresponding genes (Figure 1D and Figure S2).

**Figure 2:**
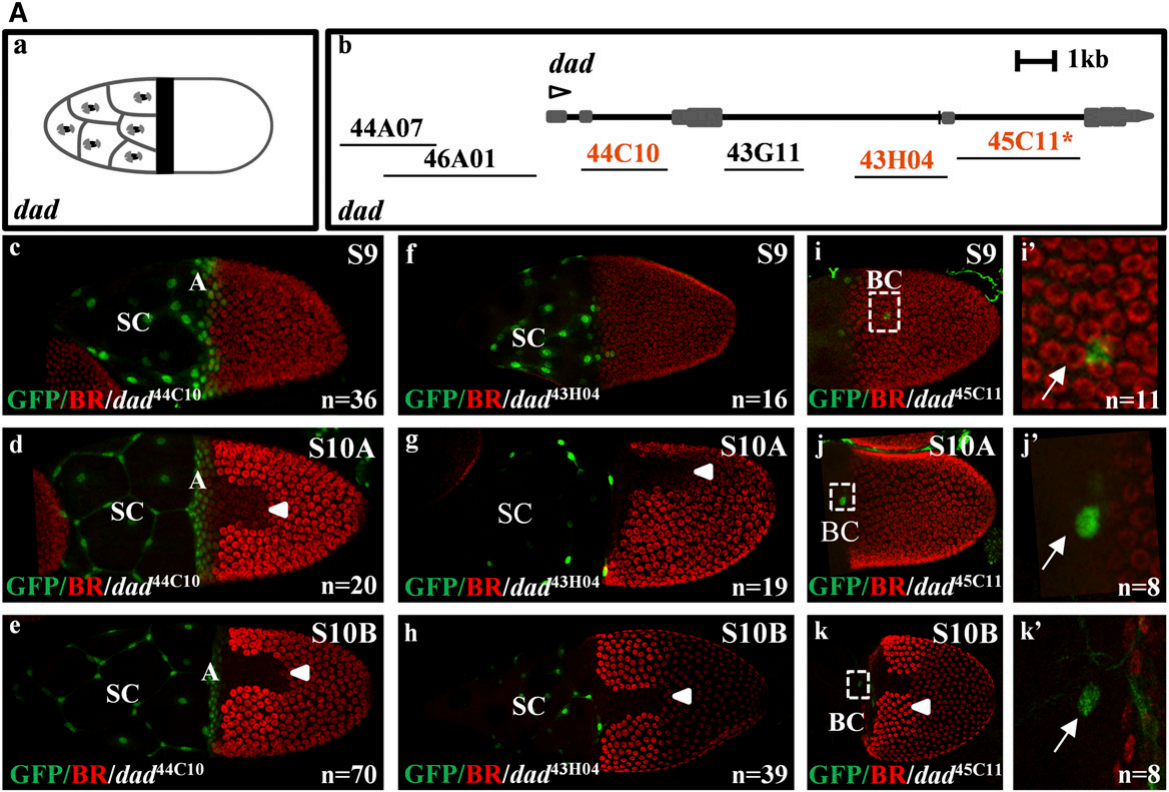

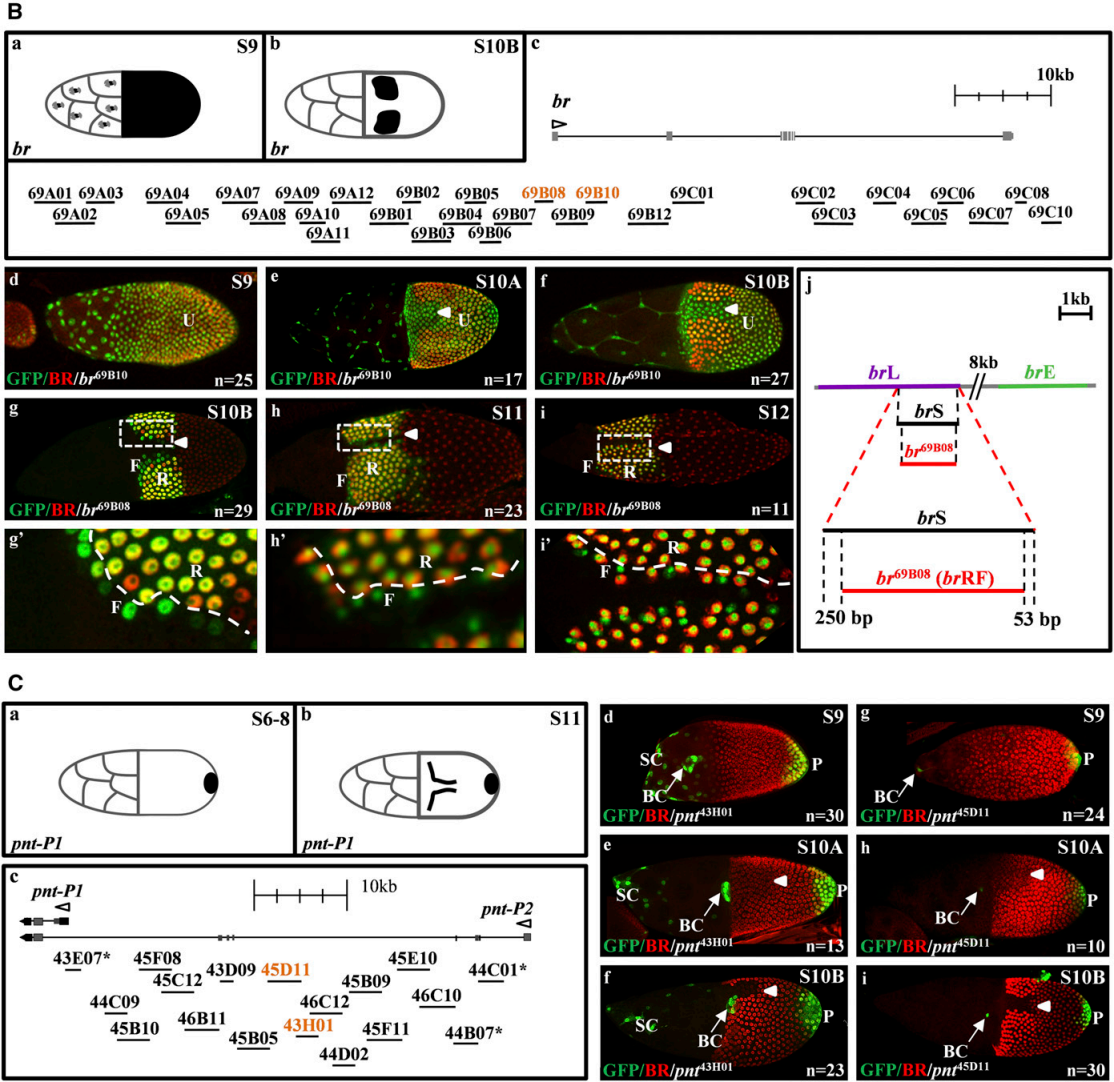

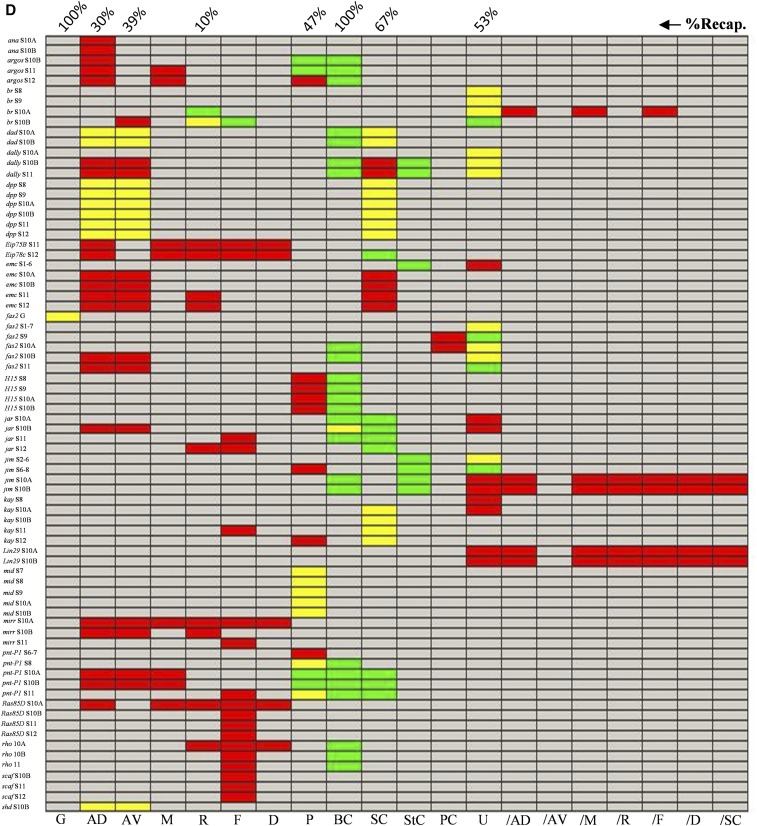
Expression domains of several FlyLight lines. (Aa) A cartoon describing the *daughters against dpp* (*dad*) expression pattern in the stretched cells (SC) and anterior (A) domains. (Ab) The gene model for *dad* and the associated FlyLight fragments screened during oogenesis. The GFP-positive lines are marked in orange. * indicates a line with expression not seen in endogenous gene patterns. Open arrowhead denotes the TSS and the direction of the gene in the locus. (Ac–e) GFP expression driven by *dad*^44C10^ during stages 9–10B in the stretched cells (SC) and centripetally migrating follicle cells, denoted as the anterior (A) domain. BR is used as a spatial marker. *n* is the number of images represented by this image. Arrowheads denote the dorsal midline. (Af–h) GFP expression driven by *dad*^43H04^ during stages 9–10B in the SC. (Ai–k) GFP expression driven by *dad*^45C11^ during stages 9–10B in the border cells (BC). (Ai′–k′) Insets of (i–k) (white arrow denotes the BC). Additional stages can be found in Figure S2D. (Ba and b) A cartoon describing the expression patterns of early and late *br*. (Bc) The gene model for *br* and the associated FlyLight fragments screened during oogenesis. The GFP-positive lines are marked in orange. (Bd–f) GFP expression driven by *br*^69B10^ during stages 9–10B is uniform in all follicle cells. (Bg–i) GFP expression driven by *br*^69B08^ during stages 10B–12 in the roof (R) and floor (F) domains (*br*RF). (Bg′–i′) Insets of (Bg–i). (Bj) The position of the different *br* fragments: *br*E, *br*L, and *br*S (Charbonnier *et al.* 2015), and *br*^69B08^ (*br*RF, this screen). *br*^69B08^ is 250 and 53 bp shorter on the left and right ends, respectively, than the *br*S fragment. Additional stages can be found in Figure S2C. (Ca and b) A cartoon describing the expression patterns of *pointed*-P1 (*pnt*-P1) during stages 6–8 in the posterior (P) domain, and at the floor (F) and P domains at stage 11. (Cc) The gene model for *pnt* isoforms and the associated FlyLight fragments screened during oogenesis. The GFP-positive lines are marked in orange. (Cd–f) GFP expression driven by *pnt*^43H01^ during stages 9–10B in the SC, border cells (BC), and P domains. (Cg–i) GFP expression driven by *pnt*^45D11^ during stages 9–10B in the BC and P domains. Additional stages can be found in Figure S2R. (D) A binary matrix representing all gene expression patterns (red) and FlyLight GFP-positive lines (green). The overlap between the two data sets is denoted in yellow. The matrix is based on assigning mutually exclusive domains to patterns (Figure 1 and Figure S2, i and ii). Domains include germarium (G), splitting the anterior domain to anterior dorsal (AD) and anterior ventral (AV), midline (M), roof (R), floor (F), dorsal (D), posterior (P), stretched cells (SC), stalk cells (StC), polar cells (PC), and uniform (U). Additional domains are included as not one of the previously listed (/) for domain exclusions. The complete description of these domains can be found in Figure S2, i and ii. On the *y*-axis is the gene name at a specified developmental stage. Percent recapitulation (%Recap.) represents the percent of GFP patterns that overlap with the endogenous pattern in each domain.

The BMP inhibitor, *daughters against dpp* (*dad*) (Inoue *et al.* 1998; Tsuneizumi *et al.* 1997), is expressed in the stretched cells and the anterior follicle cells (Jordan *et al.* 2005; Yakoby *et al.* 2008a; Muzzopappa and Wappner 2005) (Figure 2Aa). Three of the six associated FlyLight lines express GFP (Figure 2A, b–k). The *dad*^44C10^ line recapitulates the *dad* endogenous expression pattern (Figure 2A, c–e). The *dad*^44C10^ line is expressed in the centripetally migrating cells and in the stretched cells from stage 8 (Figure S2Dc). Interestingly, the *dad*^43H04^ line is restricted to the stretched cells (Figure 2A, f–h). Previously, we referred to the anterior domain as the centripetally migrating cells, which include the anterior oocyte-associated follicle cells (Figure 1, B and C) (Niepielko *et al.* 2014; Yakoby *et al.* 2008a). Here, we found that the anterior pattern is comprised of two patterns, one is restricted to the stretched cells and another includes both the stretched cells and centripetally migrating follicle cells (Figure 2A, d and g). The *dad*^45C11^ line is expressed in one to two border cells (we cannot distinguish whether these are border cells or polar cells) at stages 9–10B (Figure 2A, i–k).

The zinc-finger transcription factor *broad* (*br*) is expressed in a dynamic pattern during oogenesis. At early developmental stages, *br* is uniformly expressed in all follicle cells. Later, it is expressed in two dorsolateral patches on either side of the dorsal midline (Figure 2B, a and b) (Deng and Bownes 1997; Yakoby *et al.* 2008b). Two lines, *br*^69B10^ and *br*^69B08^, express GFP (Figure 2B, c–i). The former is expressed in a uniform pattern, similar to the early expression pattern of *br* CRM (*br*E) (Figure 2B, d–f). However, unlike the early pattern of *br*, it does not clear from the dorsal domain at stage 10A (Cheung *et al.* 2013; Fuchs *et al.* 2012). The other line, *br*^69B08^, is expressed in the roof domain, like the late pattern of the *br* CRM (*br*L) (Figure 2B, g–i). Interestingly, unlike the *br* gene and the published *br*L, this line is also expressed in the floor domain (Figure 2B, g′–i′). We further discuss these CRMs later (Figure 2Bj).

The ETS transcription factor *pointed* (*pnt*) is necessary for proper development of numerous tissues, including the eye and eggshell (Morimoto *et al.* 1996; Zartman *et al.* 2009a; Deng and Bownes 1997; Freeman 1994). Two isoforms are expressed in the follicle cells during oogenesis, *pnt-P1* and *pnt-P2* (Figure 2C, a and b). The *pnt-P1* isoform is expressed in the posterior domain from stage 6 to 9. At stages 10A and 10B, it is expressed in the dorsal midline (Morimoto *et al.* 1996; Boisclair Lachance *et al.* 2014). Later, at stage 11, the *pnt-P1* isoform is expressed in the floor and posterior domains (Yakoby *et al.* 2008a). Two overlapping FlyLight lines show a similar pattern of GFP expression (Figure 2C, c–i). The *pnt*^45D11^ and *pnt*^43H01^ lines express GFP in the posterior and border cells (Figure 2C, d–i). In addition, *pnt*^43H01^ is broadly expressed in the stretched cells (Figure 2C, d–f). None of the screened lines associated with the *pnt* gene were found to contain the information for the midline expression pattern of *pnt-P1*. The midline pattern of the *pnt-P1* transcript could be visualized by the GFP tagging of the endogenous *pnt* gene (Boisclair Lachance *et al.* 2014). In addition, none of these lines recapitulate the pattern of *pnt-P2*, which is expressed in the midline (at stage 10A) and roof (at stage 10B) domains (Figure S2Rb) (Morimoto *et al.* 1996).

To understand the overlap between the patterns of the GFP-positive lines and the endogenous gene, all patterns were annotated as previously described (Figure 2D) (Niepielko *et al.* 2014). The annotation system is based on simple domains of gene expression that are induced by cell signaling pathways, including BMP (AD, AV, SC) and EGFR (M, D, P), and domains of future dorsal appendages (R, F) (Yakoby *et al.* 2008a,b; Niepielko *et al.* 2014; Nilson and Schüpbach 1999; Peri and Roth 2000; Twombly *et al.* 1996). This system was developed to annotate follicle cell patterning as a binary matrix, which allows the addition of domains found in our screen, including germarium (G), stalk cells (StC), border cells (BC), and polar cells (PC) (Figure 1, A–C). The overlay of the GFP expression patterns and the endogenous gene patterns revealed that the majority of recapitulated patterns are within the anterior (AD, AV), stretched cells (SC), posterior (P), and uniform (U) domains (Figure 2D).

Numerous genes are uniformly expressed in the follicle cells during early oogenesis (Figure 2D). At the same time, the uniform “inducer” is still unknown. Several reporter lines are expressed in the border cells (Figure 2D). With the exception of one line, none of the known associated genes were reported to be expressed in these cells. Since the border cells travel through the nurse cells, which turn dark during most *in situ* hybridization procedures, it is possible that gene expression in the border cells is masked by the dark nurse cells (Yakoby *et al.* 2008a). The roof and floor domains (R and F) are regulated jointly by multiple signaling pathways, including EGFR and BMP (Deng and Bownes 1997; Ward and Berg 2005; Ward *et al.* 2006). It is possible that the single floor and roof patterns found is due to the complex regulation of these domains that may require more enhancers working together (Figure 2D). The midline and dorsal domains were not found in our screen; these CRMs may reside in neighboring genes, require longer DNA fragments, or are present in the gene locus but not covered by the screened fragments.

### The FlyLight lines are a new resource for gene perturbations in oogenesis

The GAL4-UAS system has been a valuable method to manipulate genes in *D. melanogaster* (Brand *et al.* 1994; Duffy 2002). To increase the perturbation efficiency, it is necessary to refine and restrict the affected domain. The GFP-positive lines present an opportunity to manipulate genes in a domain-specific manner. As far as we know, none of the previously published GAL4 lines are expressed only in the anterior domain, including the centripetally migrating follicle cells and stretched cells. Here, we used two of the anterior lines, *dad*^44C10^ and *dpp*^18E05^, to determine their function in genetic perturbations. A limitation of the GAL4-UAS system is the undesired expression of some drivers in multiple tissues, which, in many cases, leads to lethality. Indeed, a complete lethality was observed when these lines were crossed directly to UAS lines of perturbations in EGFR signaling (data not shown). Thus, we used a GAL80^ts^ to circumvent the problem.

**Figure 3:**
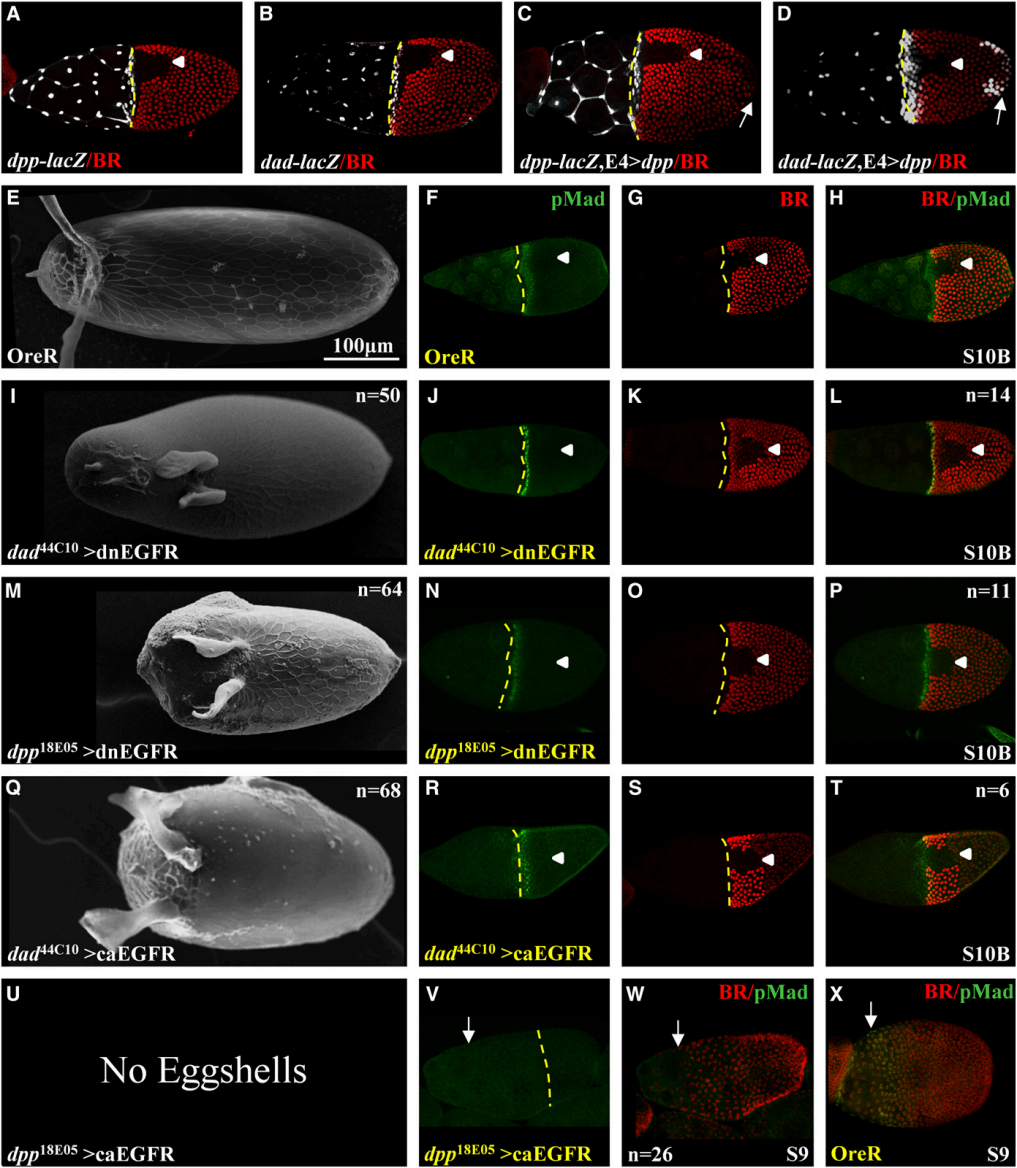
Genetic perturbations using *dad*^44C10^ and *dpp*^18E05^ FlyLight lines. (A and B) β-galactosidase expression patterns of *dad*^44C10^ and *dpp*^18E05^ lines in the anterior and stretched cells domains (*dad-lacZ* and *dpp-lacZ*). (C and D) Expression of *dpp* in the posterior end (E4*>dpp*) induces ectopic expression of β-galactosidase expression in the posterior domain in *dad-lacZ* but not in *dpp-lacZ* (denoted by a white arrow). BR staining is used as a spatial marker. Arrowheads denote the dorsal midline. Broken yellow lines denote the anterior boundary of the oocyte. (E–H) OreR (E) eggshell, (F) pMad (green), (G) BR (red), and (H) merge. (I–L) *dad*^44C10^ driving the expression of a dnEGFR: (I) eggshell, (J) pMad, (K) BR, and (L) merge. (M–P) *dpp*^18E05^ driving the expression of a dnEGFR: (M) eggshell, (N) pMad, (O) BR, and (P) merge. (Q–T) *dad*^44C10^ driving the expression of a caEGFR: (Q) eggshell, (R) pMad, (S) BR, and (T) merge. (U–X) *dpp*^18E05^ driving the expression of caEGFR: (U) no eggshell, (V) pMad [↓ denoted the anterior boundary of the future oocyte-associated follicle cells, also in (W)], and (W) merged image of pMad and BR (a separate BR image is not shown). (X) For comparison, we included the wild-type (OreR) merged BR/pMad image at S9. We note that oogenesis stopped at stage 9 in the *dpp*^18E05^ > caEGFR background. No pMad is present in egg chambers. Egg chambers’ developmental stages are denoted. All images are a dorsal view and anterior is to the left.

**Figure 4:**
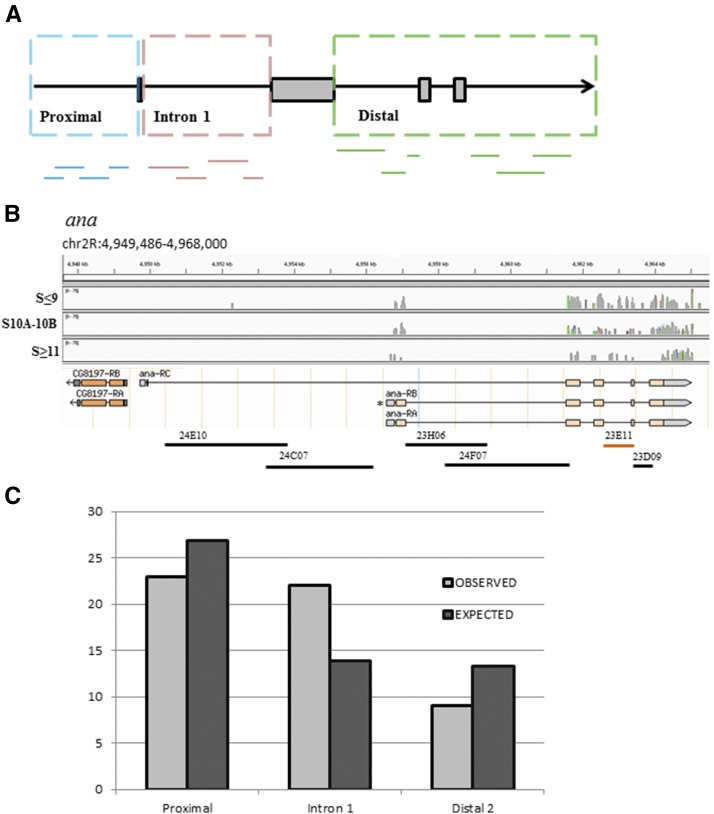
(A) A cartoon representation of the gene-fragments’ binning that is based on the relative position of a fragment in the gene model. *Proximal* includes all fragments that are upstream to the first exon. *Intron 1* includes all fragments that are in the first intron. *Distal* includes all fragments downstream of the second exon. (B) An example of one of the genes screened, *ana*, and its three isoforms. The gene has two “first” exons. Using RNA-seq data, we demonstrate in the coverage plot that the *ana*-RA and/or *ana*-RB isoforms (marked by *) are expressed during all stages of oogenesis, whereas *ana*-RC is not expressed. The RNA-seq data are divided into three developmental groups (stage 9 and younger, stages 10A and 10B, and stages 11 and older egg chambers). Peaks indicate the number of reads per base. Gray peaks indicate matched base pairs and colored peaks indicate mismatches (see *Materials and Methods* for details). The fragments mapped below the model were screened. The analysis shows that *ana*^23E11^ fragment (orange) is in the *Distal* bin. (C) A χ^2^ test shows that the GFP-expressing FlyLight fragments are distributed significantly different (*P* = 0.034, d.f. = 2) from the expected distribution.

The regulation of *dpp* during oogenesis is not fully understood. While an earlier study mapped numerous regulatory elements downstream of the 3′ end of the *dpp* transcription unit, it did not report expression during oogenesis (Blackman *et al.* 1991). The posterior repression of *dpp* requires the activation of EGFR signaling (Peri and Roth 2000; Twombly *et al.* 1996). Unlike *dpp*, *dad* is a known target of BMP signaling (Marmion *et al.* 2013; Weiss *et al.* 2010). The expression patterns of *dad*^44C10^ and *dpp*^18E05^ are nearly identical (Figure S2, D and F). However, if the two CRMs are regulated by different mechanisms, perturbations in cell signaling pathways may impact their activities in a different manner. To test this idea, we used the corresponding fragments of DNA in *dad*^44C10^ and *dpp*^18E05^ (Figure 2A, Figure 4B, Figure S1, and Figure S2, D and F) to generate *lacZ* reporter lines. As expected, the two reporter lines are expressed in a similar anterior pattern (Figure 3, A and B). To test whether these reporter lines are regulated by BMP signaling, we crossed these lines to a fly expressing *dpp* in the posterior end of the egg chamber (E4>*dpp*). Interestingly, we detected ectopic posterior expression of β-galactosidase in the *dad-lacZ* line, but not in the *dpp-lacZ* background (Figure 3, C and D). Based on the β-galactosidase results, we conclude that the two drivers are regulated differently, and thus perturbations, in addition to affecting the tissue, may have a positive or negative impact on the drivers themselves.

Eggshell structures are highly sensitive to changes in EGFR signaling (Neuman-Silberberg and Schüpbach 1993; Queenan *et al.* 1997; Yakoby *et al.* 2005). Therefore, we aimed to demonstrate the use of the two drivers to disrupt EGFR signaling and monitor the impact on eggshell structures and egg chamber patterning. Each driver was crossed to a dominant-negative EGFR (dnEGFR) and a constitutively activated EGFR (caEGFR). We looked at patterning of BR, BMP signaling (pMad), and eggshell structures. *D. melanogaster* eggshell has two long dorsal appendages. At stage 10B, pMad appears in three rows of cells in the anterior domain, while BR is expressed mostly in two dorsolateral patches on either side of the dorsal midline (Figure 3, E–H) (Deng and Bownes 1997; Yakoby *et al.* 2008b). The eggshell of *dad*^44C10^ > dnEGFR has an elongated narrow operculum and two shortened dorsal appendages (Figure 3I). Interestingly, the pattern of pMad and BR remained in one and two rows, respectively, of cells in the anterior domain (Figure 3, J–L). The *dpp*^18E05^ > dnEGFR generated a short eggshell with a large and wide operculum and two short dorsal appendages (Figure 3M). Unlike the anterior domain of pMad and BR in Figure 3L, this perturbation led to ectopic pMad in the anterior domain but not BR (Figure 3, N–P). An activation of EGFR in the posterior domain represses *dpp* expression (Twombly *et al.* 1996). Following the same logic, overexpression of dnEGFR alleviates the anterior repression of *dpp*, and consequently increases BMP signaling and the operculum size (Dobens and Raftery 1998).

Overactivation of EGFR signaling in the anterior domain with the two drivers generated different phenotypes. In *dad*^44C10^ > caEGFR, the eggshell was short with a reduced operculum that extends to the ventral domain (Figure 3Q), which is expected for an increase in anterior EGFR activation (Queenan *et al.* 1997). The pMad pattern was shifted anteriorly over the stretched cells. Also, BR was shifted anteriorly (Figure 3, R–T). These phenotypes indicate that, in addition to the increase in EGFR signaling, there is also a decrease in BMP signaling (Yakoby *et al.* 2008b). Interestingly, *dpp*^18E05^ > caEGFR did not produce any eggs (Figure 3U). The ovarioles and egg chambers of flies grown at 30° appeared deformed (data not shown). Reducing the temperature to 28° allowed the egg chambers to develop up to stage 9. However, pMad could not be detected (Figure 3, V and W) in comparison to the corresponding pMad pattern in the wild type at this developmental stage (Figure 3X). This cross was repeated five times and all egg chambers ceased development at stage 9. These results are consistent with the previously published decrease in BMP signaling: a medium decrease generated short eggshells, while a strong decrease stopped egg chamber development at stage 9 (Twombly *et al.* 1996). These results further support the negative regulation of *dpp* by EGFR activation.

### Mapping the distribution of CRMs in the gene model

To date, the prediction of CRMs has not been straightforward. Since the FlyLight fragments cover the entire length of the gene, we aimed to determine whether certain locations of the gene locus are more likely to contain CRMs. We binned the distributions of all GFP-positive FlyLight lines into three groups, based on their relative position to the first exon of the gene model (Figure 4A). All DNA fragments that are upstream of the first exon were classified as *Proximal*. All fragments that are downstream of the first exon and within the first intron are categorized as *Intron 1*, and all other downstream fragments are classified as *Distal*. One problem with this analysis is that several genes have multiple isoforms with different locations of the first exon. For example, the *ana* gene has three isoforms, two have the same first exon (*ana*-RA and *ana*-RB) and the third (*ana*-RC) has a different first exon (Figure 4B). Since no information is available on the oogenesis-specific isoform(s), we carried out an RNA-seq analysis of egg chambers at three developmental groups. Specifically, egg chambers were collected at early (stages ≤9), middle (stages 10A–B), and late (stages ≥11) stages of oogenesis. We found that the *ana* gene has only two isoforms (*ana*-RA and *ana*-RB) that could be expressed during oogenesis; both have the same TSS (Figure 4B). The RNA-seq analysis eliminated discrepancies among isoform transcripts for nine additional genes (Figure S2).

Next, we tested the distribution of the GFP-positive FlyLight lines in the three categories (*Proximal*, *Intron 1*, and *Distal*). The null hypothesis is that the frequency of CRMs among the categories is identical. In this case, the observed frequency of positive CRMs is equal to the expected frequency of positive CRMs for the total number of DNA fragments for each of the three categories. The expected distribution is calculated as the percent of the number of GFP-positive lines (54) out of the total number of lines (281), which is 19%. The *Proximal* category includes 140 lines. The expected number of lines expressing GFP in this category is 27. The observed number of GFP-expressing lines is 23, which is 15% less than the expected value (Figure 4C). The *Intron 1* category includes 72 lines, thus the expected number of lines expressing GFP is 14. The observed number of GFP-expressing lines is 22, which is 57% more than the expected value (Figure 4C). The *Distal* category includes 69 lines, and the expected number of lines expressing GFP is 13. The observed number of GFP-expressing lines is 9, which is 31% less than the expected value (Figure 4C). Using a χ^2^ test, we determined that the distribution of the observed values is significantly different from the expected values (*P* = 0.034, d.f. = 2).

To determine whether the distribution of CRMs is significantly different than the expected value for each category, we used a binomial test, which checks the significance of deviation between two results. As stated above, the calculated success rate (positive GFP expression) is 19%. Based on this rate, we employed a one-tailed binomial test for each category. The probability that 23 or less out of the 140 fragments in the *Proximal* category will drive GFP expression is 0.25. The probability that 22 or more out of the 72 fragments in the *Intron 1* category will drive GFP expression is 0.012. The probability that 9 or less out of the 69 fragments in the *Distal* category will drive GFP expression is 0.13. Based on the binomial test, we conclude that the number of CRMs in the *Intron 1* category is significantly greater (*P* = 0.012) than the expected number. We note that the average DNA fragment size for the *Proximal* is 3.17 ± 0.06 kb, for the *Intron 1* is 3.06 ± 0.1 kb, and for the *distal* is 2.56 ± 0.13 kb (fragment size ± SE kb). While the average size of the *Distal* fragments is significantly shorter than the fragments in the other two categories (*P* < 0.01), we do not consider this difference to be the cause for the CRM enrichment in *Intron 1*, since shorter fragments were found to be more active (Nam *et al.* 2010). Also, the average fragment size is not significantly different between the *Proximal* and *Intron 1* categories.

### The FlyLight lines control expression in multiple tissues

**Figure 5:**
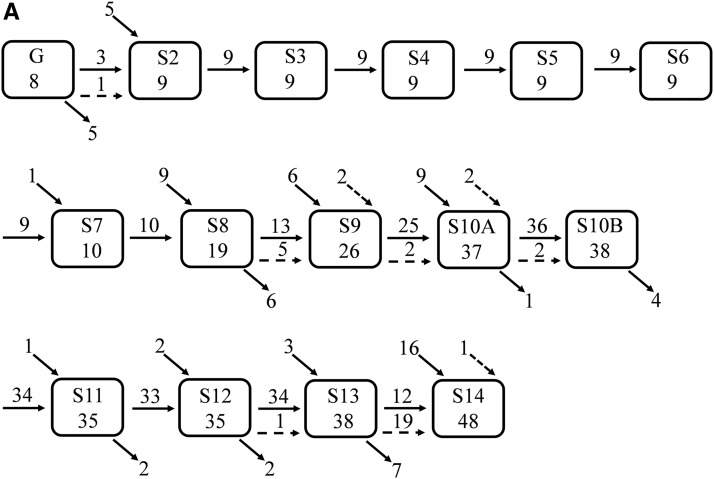

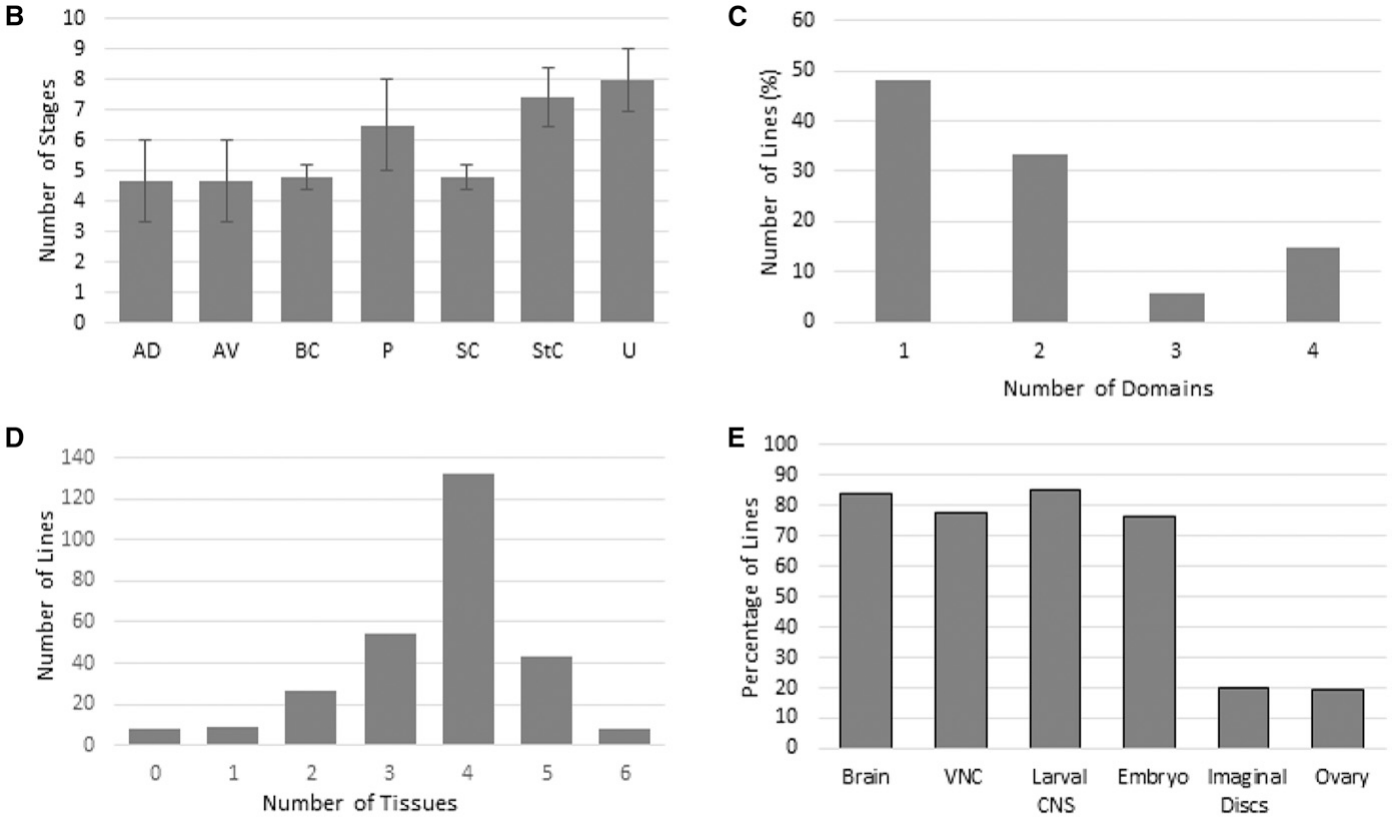
(A) A summary of the temporal distribution of GFP-positive FlyLight patterns throughout oogenesis. Each box represents a developmental stage and the number of lines expressing GFP. Horizontal arrows represent the number of lines with the same spatial pattern in the next developmental stage (referred to in the text as line patterns). Diagonal bottom arrows represent the number of lines not expressed in the next developmental stage. The diagonal top arrows represent the new lines expressed in this developmental stage. Horizontal broken arrows represent the number of lines expressed in the next developmental stage that change their spatial pattern. Diagonal broken arrows represent lines expressed in an early developmental stage, and now are expressed in a later developmental stage that is not the proximal stage (the pattern is spatially different from the earlier pattern). (B) For each domain, the average number of developmental stages it is expressed in and the SD were calculated. (C) For each of the GFP-positive FlyLight lines, the number of expression domains was calculated. The data are presented as percentage of the total lines expressing GFP for all developmental stages. (D) Based on the available data for the Flylight expression patterns, we determined the frequency of expression of each of the 281 lines in the five FlyLight tissues and oogenesis (total of six tissues). (E) Of the 281 lines screened, 84% are expressed in the brain, 78% in the ventral nerve cord, 85% in the larval CNS, 77% in the embryo, 20% in the third instar larvae imaginal discs, and 19% in the ovary.

On average, the FlyLight lines contain ∼3-kb fragments of DNA. We were interested to understand whether these fragments contain one or more CRMs regulating expression during oogenesis. Assuming that each domain is regulated by a CRM, we aimed to determine if each fly line controls one or more domains during oogenesis. Each GFP expression domain was counted once for each line per developmental stage for a total of 339 “line patterns.” Interestingly, only 35 line patterns (10.3%) changed to a different pattern in the next developmental stage (30 line patterns) or at a later stage that is not the next stage (five line patterns) (Figure 5A). Most (34 of the 35) line-pattern changes are found after stage 8, which is the transition stage from anteroposterior axis to a dorsoventral axis determination as a result of changes in the position of EGFR signaling (Neuman-Silberberg and Schüpbach 1993). Interestingly, 20 line patterns change at stage 14, the stage of ovulation. Hence, most domains, once expressed, maintain the same pattern over multiple developmental stages (Figure 5B). CRMs are 500–1000 bp (Ivan *et al.* 2008; Levine and Tjian 2003). Under the assumption that all fragments contain CRMs, it is expected that each fragment has three or more CRMs. Our results suggest that the ∼3-kb DNA fragments mostly contain one (48%) or two (33%) CRMs that control the expression of a simple pattern during oogenesis (Figure 5C).

Many CRMs are tissue specific, therefore we wanted to determine whether the same fragments control expression in other tissues (Jenett *et al.* 2012; Jory *et al.* 2012; Manning *et al.* 2012; Li *et al.* 2014). Only 8 out of the 281 FlyLight lines are not expressed in any of the examined tissues (Figure 5D). A primary portion of the lines (∼50%) are expressed in four tissues (Figure 5D). These analyses cannot distinguish whether the same CRM is expressed in multiple tissues, or if each fragment contains numerous CRMs that are expressed in different tissues. Of the FlyLight lines positive for GFP, 75–85% are expressed in other tissues, including the brain, ventral notochord (VNC), larva CNS, and embryo (Figure 5E). Only ∼20% of the lines are also expressed in imaginal discs (Figure 5E and Figure S3). These results support our observation that lethality of the tested lines in perturbations without GAL80^ts^ is likely related to the expression of these lines in multiple tissues.

## Discussion

Simple expression domains, called primitives, have been used combinatorially to recapitulate the entire complexity of follicle cell patterning (Yakoby *et al.* 2008a). The initial set of primitives was comprised of six nonmutually exclusive domains. In a later study of the *Chorion protein* gene family, these primitives were further divided into mutually exclusive domains (Niepielko *et al.* 2014). It was hypothesized that these domains are regulated by discrete CRMs. With the exception of the midline and dorsal domains, our screen successfully found the associated basic patterns (Figure 1 and Figure S2), which further supports the combinatorial assembly of CRMs as a mechanism to pattern tissues. Our screen characterized a resource of GAL4 drivers that covers all stages of egg development (from the germarium through stage 14). Furthermore, within each of these stages, we identified discrete expression domains. Thus, in addition to identifying new CRMs, this screen provides valuable tools for spatiotemporal perturbation in multiple tissues during oogenesis.

### Multiple CRMs control the dynamics of gene expression

An example for the complexity of CRM analysis is found in the regulation of the transcription factor BR. This gene is necessary for dorsal appendage formation on the *Drosophila* eggshell (Deng and Bownes 1997; Tzolovsky *et al.* 1999). In the follicle cells, the dynamic expression of *br* is regulated by two independent CRMs (Cheung *et al.* 2013; Fuchs *et al.* 2012; Charbonnier *et al.* 2015). The *br*E is uniformly expressed during early stages of egg development. Later, at stage 10A, it clears from a broad dorsal domain by the activity of the transcription factor Mirror (MIRR). This domain is later occupied by the *br*L pattern (Cheung *et al.* 2013; Fuchs *et al.* 2012). We found another uniform CRM (*br*U), which is not repressed in the dorsal domain (Figure 2B). In this case, while independent from the *br*E CRM (Figure 2B, c and j), a MIRR binding site may be absent from the *br*U isolated segment. In the genome context, it may use the same binding site that is associated with the *br*E enhancer. Further analysis is needed to determine whether *br*U functions during *br* expression in the genomic context.

As mentioned above, the *br*L enhancer is expressed in two dorsolateral patches on either side of the dorsal midline. The Pyrowolakis Laboratory demonstrated by CRM analysis that a shortened *br*L enhancer (*br*S) lost anterior and midline repression due to the loss of pMad/Brk and Pointed (PNT) binding sites, respectively (Charbonnier *et al.* 2015). Interestingly, a shorter enhancer, *br*RF, not only reversed the loss of midline repression, as seen in *br*L, it also produced a precise new pattern that, in addition to the roof domain, is also active in the floor domain (Figure 2B, g–i). It was previously reported that the roof/floor boundary is regulated by Notch signaling (Ward *et al.* 2006). In this case, the expression in the floor domain may reflect the loss of Notch pathway regulation in zones 53 and/or 250 bp, two regions of DNA that are included in the *br*S but not in the *br*RF (*br* roof/floor, also denoted as *br*^69B08^) (Figure 2Bj). Alternatively, the Fos/Jun leucine zipper transcription factors are expressed in the floor domain (Dequier *et al.* 2001; Dobens *et al.* 2001; Ward and Berg 2005). The loss of zones 53 and 250 could have eliminated a target repressor of the Fos/Jun complex. The posterior boundary of the *br* pattern is regulated by the transcription factor Midline (Mid) (Fregoso Lomas *et al.* 2013). The *br*RF overlaps with the endogenous posterior boundary of BR, thus *br*RF is likely still regulated by Mid. Further analysis is required to determine the repression mechanism(s) of *br*RF in the dorsal midline and anterior domains. Understanding the regulatory mechanisms of *br* may shed light on the evolution of eggshell morphologies (Pyrowolakis *et al.* 2017).

### The same domain can be regulated by different regulatory mechanisms

An approach to CRM discovery is the search for coexpression of genes (Gallo *et al.* 2006; Konikoff *et al.* 2012). The assumption is that spatiotemporal overlapping domains have a similar regulatory mechanism. The anterior follicle cells are patterned by BMP signaling during egg development (Berg 2005; Deng and Bownes 1997; Dobens and Raftery 1998; Twombly *et al.* 1996; Yakoby *et al.* 2008b; Fuchs *et al.* 2012). Genes like *dad* and *wit* are expressed in the anterior domain and are targets of BMP signaling (Marmion *et al.* 2013; Weiss *et al.* 2010). Two CRMs, *dad*^44C10^ and *dpp*^18E05^, are expressed in the same anterior pattern (Figure 3, A and B). While the *dad* CRM is ectopically expressed by BMP signaling, the *dpp* CRM is not (Figure 3, C and D). Thus, different mechanisms pattern the anterior domain of the follicle cells. It was previously reported that in a *grk* null background, a *dpp* enhancer trap was ectopically expressed in the posterior end of the egg chamber, a domain that does not express *dpp* in wild-type flies (Twombly *et al.* 1996). Further evidence was obtained by the ectopic expression of *dpp* in the posterior end of cornichon null egg chambers, which reduced EGFR signaling in this domain (Peri and Roth 2000). This suggests that *dad* and *dpp* are regulated by BMP and EGFR signaling, respectively.

The finding that the two anterior CRMs are regulated by different signaling pathways may account for the differences in tissue patterning and eggshell morphologies in perturbations (Figure 3). Specifically, the large operculum obtained in *dpp*^18E05^ > dnEGFR and *dad*^44C10^ > dnEGFR backgrounds may be due to the reduction in anterior EGFR signaling that, consequently, alleviated repression of the endogenous anterior *dpp*, as expected for an increase in BMP signaling (Dobens and Raftery 1998; Twombly *et al.* 1996). At the same time, the operculum size is larger in the *dpp*^18E05^ > dnEGFR background, likely due to the direct increase of the *dpp* driver activity as a result of the reduction in EGFR signaling. Interestingly, in both backgrounds, pMad remained in the anterior domain. However, only in *dad*^44C10^ > dnEGFR was BR also present in a two-cell-wide anterior domain (Figure 3, J, K, N, and O). The difference can be explained by the lack of PNT induction in the anterior domain in both backgrounds. However, due to an amplified impact on both endogenous *dpp* and the *dpp*^18E05^ driver, the anterior BMP signaling was further increased and repressed the late expression of BR in the anterior domain (Figure 3, K and O) (Fuchs *et al.* 2012).

Increasing EGFR signaling with the *dad*^44C10^ > caEGFR generated an operculum that is reduced and expands to the ventral domain, as expected for the increase in EGFR signaling (Queenan *et al.* 1997). The BR domain also shifted anteriorly, as expected for a reduction in BMP signaling (Yakoby *et al.* 2008b). In addition, the eggs observed in this background are shorter than the wild-type eggs, which is consistent with reduction in BMP signaling (Twombly *et al.* 1996). No eggs were laid in the *dpp*^18E05^ > caEGFR background. Egg chambers in this background stopped developing at stage 9, which is in agreement with a severe reduction in BMP signaling (Twombly *et al.* 1996). In both cases, crosses with *dpp*^18E05^ produced more severe phenotypes than in the *dad*^44C10^ background. In addition to the different effects of the type of signaling perturbation affecting the GAL4 line, the *dpp* driver is expressed earlier than the *dad* driver (Figure S2iii), which is consistent with *dad* being a target of DPP signaling. While a follow-up study on the activation/repression of these drivers is needed, these findings allow the tailoring of perturbations’ intensities by different combinations of drivers and UAS lines.

### The distribution of CRMs in different domains of the gene model is not equal

Given the challenges in identifying enhancers within genes, it was encouraging to find a significant enrichment of them in the first intron (*P* = 0.012). Our study used the *Drosophila* synthetic core promoter (Pfeiffer *et al.* 2008), while other screens used primarily an HSP70 minimal promoter. In a high-throughput screen, enrichment of enhancers was found within the first intron (Arnold *et al.* 2013), which is in agreement with our findings. At the same time, it is important to note that choice of promoter may impact the activity of the enhancer and consequently the level of reporter gene expression. The *Drosophila* synthetic core promoter was found to be as potent as most tested endogenous promoters; however, other changes, including in the 3′ UTR and the number of transcription factor binding sites, affect the levels of transcription (Pfeiffer *et al.* 2008, 2010). Additionally, a housekeeping core promoter is used by a different set of enhancers, which tend to contain promoters themselves or be located in the 5′ proximal region of the gene. In contrast, the enhancers of developmental genes are predominantly enriched in introns (Zabidi *et al.* 2015). Screening for CRMs in the sea urchin, Nam *et al.* (2010) found that the 5′ proximal region is the most enriched, followed by the first intron. At the same time, this screen considered only temporal expression, while our screen was based on spatiotemporal expression. Since we used the FlyLight collection, the promoter/enhancer combination was already set. In the future, it will be interesting to determine whether each gene’s endogenous promoter and 3′ UTR play a role in the patterning of the corresponding genes.

## Supplementary Material

Supplemental material is available online at www.g3journal.org/lookup/suppl/doi:10.1534/g3.117.043810/-/DC1.
