# Supplementary material for: Simple Expression Domains Are Regulated by Discrete CRMs During Drosophila Oogenesis
Source: G3 (Bethesda). 2017 Jun 20;7(8):2705–18. doi: 10.1534/g3.117.043810 (PMC5555475; doi:10.1534/g3.117.043810)
Supplement: Supplementary file 1 [file 2705FigureS1.pdf]

Gene: Flylight Stock (Bloomington Stock)

***ana*, CG8084:** 23D09 (45126), 24F07 (45502), 23E11(49033), 23H06 (49050), 24C07 (49074)

***aos*, CG4531:** 24D09 (45846), 24G11 (45504), 25C01 (49115), 24C05 (49313)

***br*, CG11491:** 69A01 (39472), 69A02 (39473), 69A03 (39474), 69A04 (46588), 69A05 (46589), 69A07 (39475), 69A08 (46590), 69A09 (46591), 69A10 (39476), 69A11 (46592), 69A12 (39477), 69B01 (46593), 69B02 (39478), 69B03 (46594), 69B04 (41355), 69B05 (39479), 69B06 (39480), 69B07 (46595), 69B08 (39481), 69B09 (47391), 69B10 (46596), 69B12 (46597), 69C01 (39482), 69C02 (39483), 69C03 (46598), 69C04 (39484), 69C05 (39485), 69C06 (46092), 69C07 (39486), 69C08 (46599), 69C10 (46600)

***dad*, CG5201:** 44A07 (41261), 43G11 (47930), 43H04 (46251), 44C10 (45721), 45C11 (47600), 46A01 (46264)

***dally*, CG4974:** 53A07 (47375), 71A10 (39562), 60A10 (39234), 56G11 (47381), 59G08 (48226), 71G02 (47725), 59H01 (46423), 56H10 (NA), 53H08 (50454), 56H02 (39164), 60B04 (45892), 53B12 (49584), 71A06 (46647), 71B09 (46653), 71E03 (39587), 71E04 (46658), 71E07 (39590), 71F09 (46662), 71F06 (39596), 71F08 (48304), 59F12 (48224), 53F03 (48197), 71C10 (39576), 71D08 (NA), 71D04 (48300), 60D01 (39245)

***dpp*, CG9885:** 16G02 (47472), 17B11 (47474), 18B08 (45437), 19B04 (48839), 18D08 (45442), 18E05 (45107), 19D09 (45833), 19E03 (45111), 19E02 (49833), 17E04 (48770), 17G08 (48784), 19C03 (49283)

***Eip75b*, CG8127:** 44A12 (45714), 45A08 (47934), 45A09 (50219), 44D06 (45722), 43D02 (50182), 46D02 (50263), 45D02 (49561), 44D01 (50204), 44D07 (50207), 44E07 (45312), 46E12 (45747), 45E03 (50229), 43E02 (50186), 44G04 (45727), 44H03 (45729), 44H05 (45317), 44H06 (45318), 45H09 (46263), 45H10 (47937), 43H07 (48153), 45A02 (46525), 45A11 (45734), 45B12 (45322), 45B06 (49560), 46B12 (50255), 45G03 (46261), 46C07 (46269), 45C07 (48159), 46C06 (50261), 46F01 (45748), 46F05 (45328), 46F06 (45329)

***Eip78c*, CG18023:** 34G12 (45613), 36A03 (46207), 36A04 (49372), 36D03 (45231), 35D08 (49370), 35B06 (49817), 35B09 (49819), 35B10 (49820), 36C01 (49930), 34H04 (49804)
35E02, (49911)

***emc*, CG1007:** 10B08 (47314), 10H11 (47450), 10D04 (48257), 10B05 (48242), 10C04, (48249)

***fas2*, CG3665:** 10E01 (45004), 10H08 (45007), 12B04 (45021), 10F01 (45790), 10C11 (46123), 11D10 (46130), 10D12 (47315), 11D06 (47317), 10H06 (47846), 11A09 (47847), 11B05 (47848), 10B09 (48245), 10D07 (48259), 10F06 (48265), 10F07 (48266), 10H04 (48275), 11B04 (48286), 11C05 (48291), 10E04 (48439), 11C08 (48449), 11D09 (48456), 11E04 (48458), 11F03 (48464), 11F10 (48468), 11G01 (48469), 11G06 (48471), 11H09 (48478), 10C09 (49235), 10E06 (49236)

***H15*, CG6604:** 85A01 (40410), 85A02 (48627), 85A03 (40411), 85A04 (40412), 85A05 (47966), 85A07 (40413), 85A08 (46791), 85A09 (47805), 85A10 (40414), 85A11 (40415), 85A12 (47967), 85B01 (47968), 85B02 (47969), 85B03 (46792), 85B04 (40416), 85B05 (46793), 85B06 (40417), 81H10 (45366), 81H11 (47109)

***jar*, CG5695:** 90C12 (47129), 42H09 (50174), 45A05 (50218), 44B06 (38877), 43H10 (41260), 45F03 (50235)

***jim*, CG11352:** 80D09 (47061), 80D10 (40079), 80E01 (47062), 80E02 (40080), 80E03 (47063), 87E03 (45382), 87E04 (45923)

***kay*, CG33956:** 39E11 (45676), 41B11 (41240), 42B10 (50146), 39H03 (45264), 41G10 (45973), 41G05 (41243), 40G11 (48143), 42A04 (45283), 42A05 (45974), 42E11 (45292), 42F11 (45703), 39F12 (50060), 41C11 (47925)

***Lin29*, CG2052:** 40C06 (49385), 40C03 (45681), 40C10 (50081), 39C08 (50040), 40D08 (45270), 41D05 (45277), 42D06 (50153), 40F01 (50092), 40F02 (50093), 39F03 (45262), 36F07 (49938), 40H01 (46522), 40H06 (45688), 40H08 (45272), 40G01 (50098), 40G09 50100), 39G02 (50062)

***mid*, CG6634:** 86F08 (40463), 86F11 (40464), 86G04 (40467), 86G08 (47818), 86G05 (46825)

***mirr,* CG10601:** 32G07 (45210), 33B03 (45599), 34B11 (49774), 33B08 (45600), 34C01 (46200), 34C07 (49896), 33C10 (49744), 34C02 (49775), 34C05 (49778), 34D12 (47550), 34D08 (48122), 34E06 (50419), 33E04 (49752), 33H11 (48119)

***pnt,* CG17077:** 43E07 (45304), 44B07 (45717), 45B05 (45735), 45B10 (50223), 46B11 (50254), 45B09 (47935), 45C12 (45736), 46C10 (46271), 44C09 (41263), 46C12 (47938), 44C01 (48154), 45D11 (49563), 43D09 (49553), 44D02 (50205), 45E10 (50233), 45F11 (50239), 45F08 (49565), 43H01 (47931)

***Ras85d,* CG9375:** 13G12 (48587) 57A08 (39169)

***rho,* CG1004:** 37F01 (49378), 38A01 (45661), 37F11 (49379), 38A06 (49977), 38A10 (48134), 38F06 (45254), 37G12 (49967), 38A08 (49979), 38H04 (50027)

***scaf,* CG11066:** 50E05 (47622), 50H04 (46004), 51C04 (46016), 51D06 (46019)

***shd,* CG13478:** 81A02 (47770), HGTX 81A07 (40100), HGTX 81B02 (40101), HGTX 81A09 (47082), HGTX 81B05 (46099), HGTX 81A04 (48363)
