# Supplementary material for: Simple Expression Domains Are Regulated by Discrete CRMs During Drosophila Oogenesis
Source: G3 (Bethesda). 2017 Jun 20;7(8):2705–18. doi: 10.1534/g3.117.043810 (PMC5555475; doi:10.1534/g3.117.043810)
Supplement: Supplementary file 2 [file 2705FigureS2.pdf]

Figure S2: Collection of FlyLight lines expressed during oogenesis

Fig S2 i. Domain annotation

Abbreviation of patterns used:
AD: anterior dorsal
AV: anterior ventral
M: midline
R: roof
F: floor
D: dorsal
P: posterior
BC: border cells
SC: stretched cells
StC: stalk cells
PC: polar cells
U: uniform
Op: operculum
DA: dorsal appendages

Fig S2 i. Abbreviations of patterning domains used to annotate all GFP expression patterns in this Supplemental Material, Figure S2. The next page has the cartoons representing the spatial patterns of the annotated domains. With the exception of the cartoons of the germarium and early developmental stages, and the two egg chambers at stage 14, all egg chambers are shown at stage 10 (S10). The anterior end is to the left and posterior end is to the right. Unless otherwise stated, all egg chambers are in a dorsal view. While the morphology of the egg chamber changes during oogenesis, the expression pattern remains in the same sub group of cells.

Fig S2 i. continued

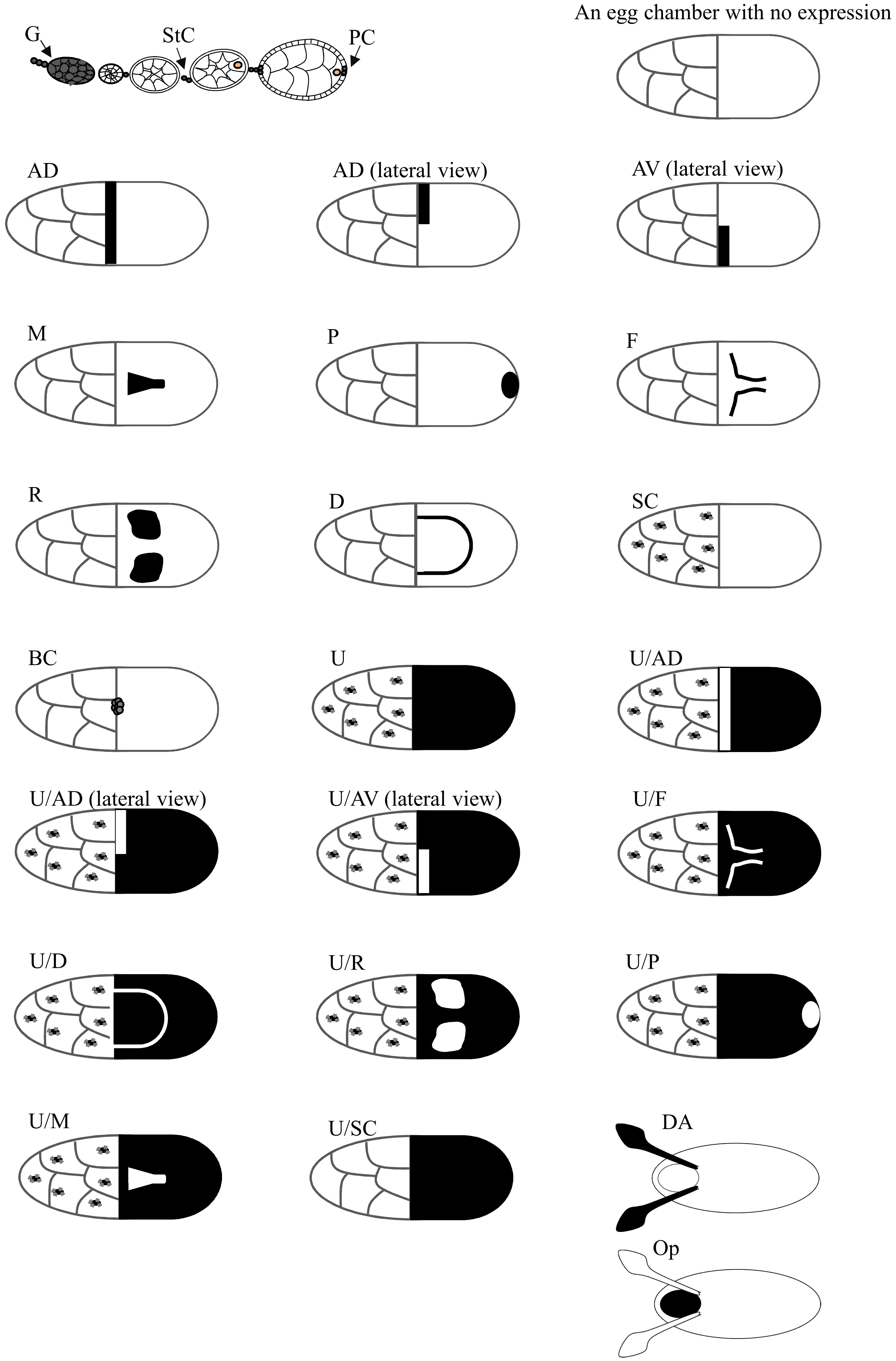

Fig S2 ii. A collection of gene/line pattern annotations per stage

CG	FlyLight Stock	Total Patterns	G	S2	S3	S4	S5	S6	S7	S8	S9	S10A	S10B	S11	S12	S13	S14
CG8084	23E11	2												SC	SC	SC	Op
CG4531	24G11	4								P	P	BC	BC, P	BC	BC	SC	Op
CG11491	69B08	3										R	R, F	R, F	R, F	R, F	DA
	69B10	1								U	U	U	U	U	U	U	U
CG5201	43H04	2	G								SC	SC	SC	SC	SC	SC	Op
	44C10	4									AD, AV, SC	AD, AV, SC	AD, AV, SC	AD, AV, SC	AD, AV, SC	AD, AV, SC	Op
	45C11	1									BC	BC	BC				
CG4974	56H02	1															BC
	60D01	1		StC	StC	StC	StC	StC	StC	StC							
	71D08	2		StC	StC	StC	StC	StC	StC	StC, U	U	U	U	U	U	U	U
	71E07	2		StC	StC	StC	StC	StC	StC	StC	StC, BC	StC, BC	StC, BC	StC, BC	StC	StC	BC
CG9885	17E04	1															U
	17G08	1															BC
	18E05	4								SC	AD, AV,SC	AD, AV,SC	AD, AV,SC	AD, AV,SC	AD, AV,SC	AD, AV,SC	Op
	19B04	1															BC
	19E02	1															Op
CG8127	44A12	1															DA
	44D06	2										SC	SC	SC	SC	SC	Op
	45A08	1											SC	SC	SC		
	45B06	2	G								BC	BC	BC				BC
	45C07	2													SC	SC	DA
	46E12	2										SC	SC	SC	SC	SC	DA
CG18023	35D08	1	G														
	36C01	1															BC
CG1007	10B05	1	G														
	10D04	1		StC	StC	StC	StC	StC	StC	StC							
CG3665	10E06	1	G														
	11G06	4	G	U	U	U	U	U	U	U	U	U, BC	U, BC	U, BC	U, BC	U, BC	DA
CG6604	85B02	1									BC	BC	BC	BC	BC	BC	BC
CG5695	44B06	3								SC	SC, BC	SC, BC	SC	SC	SC	SC	Op
	45F03	2															DA, P
CG11352	80D09	2	G	U	U	U	U	U	U	U							
	80D10	1															U
	80E01	4	G	StC, U	StC, U	StC, U	StC, U	StC, U	StC, U	StC, U		StC, BC	StC, BC	StC, BC	StC, BC	StC, BC	BC
CG33956	39H03	3										SC	SC	SC	SC	SC	Op, P
	41G05	1															P
	41G10	1															U
	42E11	2									SC	SC	SC	SC	SC	SC	U
	42A05	2															DA, P
CG2052	40C06	1		U	U	U	U	U	U	U							
	40G09	1															BC
CG6634	86F11	1							P	P	P	P	P	P	P	P	P
	86G04	2								P	P	P	P	P	P	P	P, BC
CG10601	34B11	2													SC	SC	Op
	34C01	1															P
CG17077	43H01	4								P, BC	P, BC, SC	P, BC, SC	P, BC, SC	P, BC, SC	P, BC, SC	P, BC, SC	P, DA
	44B07	2														SC	DA
	45C12	2														SC	DA
	45D11	2								P, BC	P, BC	P, BC	P, BC	P, BC	P, BC	P, BC	P, BC
	45E10	1															DA
CG9375	57A08	1															U
CG1004	38A01	1										BC	BC	BC	BC	BC	BC
CG13478	81A04	2														SC	DA
	81A07	4										AD, AV, SC	AD, AV, SC	SC	SC	SC	BC

Fig S2 ii. A collection of all annotations for each gene (CG#), per FlyLight line (stock #), for each developmental stage. Empty boxes represent no expression for this line at the specified developmental stage. The annotations dorsal appendages (DA) and operculum (Op) are reserved for S14 eggshells where we do not distinguish between roof and floor or midline, anterior dorsal, and anterior ventral. The total number of expression domains also appear in the table and a summary can be viewed in Fig. 5C.

Fig S2 A-V. For all RNA-seq coverage plots in the following figures, the peaks in the coverage plot represent the number of reads per base pair. The color code represents miscalls or SNPs to the reference genome. In these cases, red, blue, orange, and green represent cytosine, thymine, guanine, and adenosine mismatches, respectively, and gray represents a match.

Aa. *anachronism (ana)*, CG8084

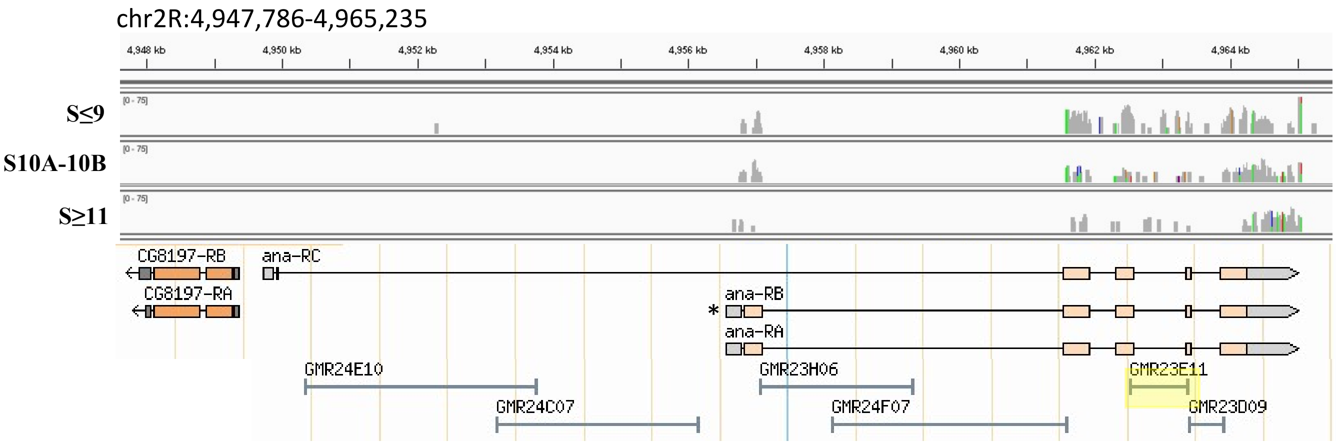

Ab.

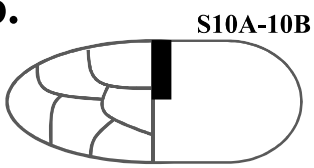

Ac.

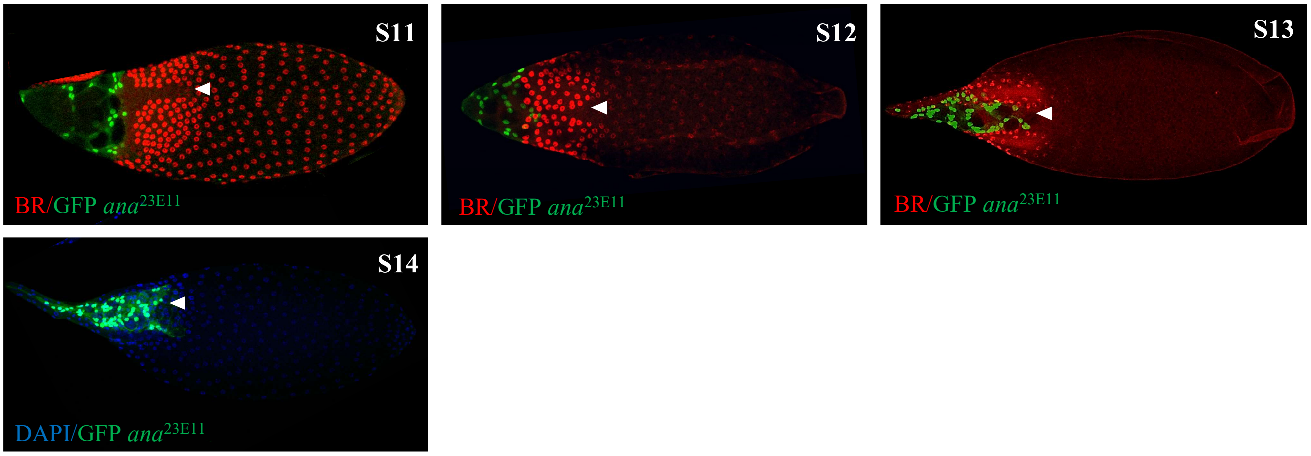

Fig S2 Aa. Gene model for *ana* with RNA-seq coverage plot showing isoforms RA/RB expressed during oogenesis. Reads per base range between 0-75 at all developmental stages. Fragments mapped below model indicate individual fly lines screened. Highlighted box (yellow) represents the line positive for GFP expression. **Ab.** Depiction of *ana* expression in AD at S10A-10B shown in lateral view (Yakoby et al. 2008). **Ac.** Line *ana*^{23E11}-GAL4 expressing GFP in SC at S11- 13 and in Op at S14. Arrowhead denotes the dorsal midline. Anterior is to the left.

Ba. *argos (aos)*, CG4531

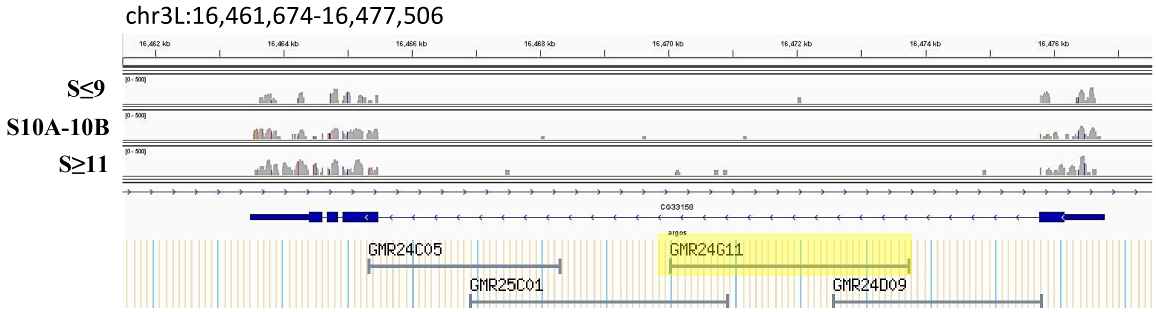

Bb.

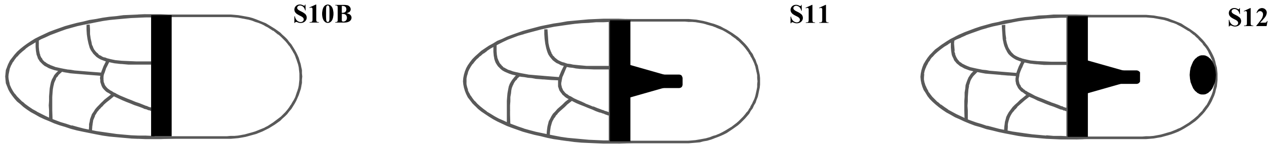

Bc.

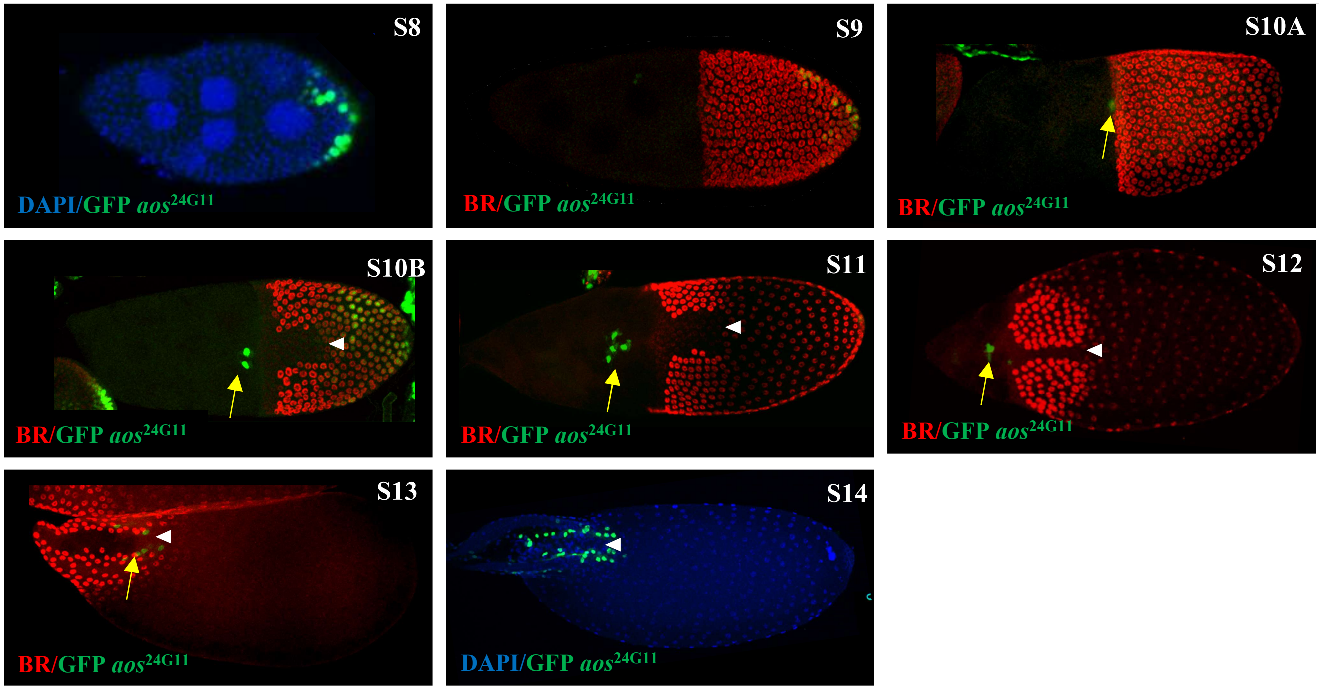

Fig S2 Ba. Gene model for *argos* with RNA-seq coverage plot showing only one active isoform during oogenesis. Reads per base range between 0-500 at all developmental stages. Fragments mapped below model indicate fly lines screened. Highlighted box (yellow) represents the GFP positive line. **Bb.** Depiction of endogenous *argos* expression in AD at S10B, AD and M at S11, and AD, M, and P at S12 (Queenan et al. 1997). **Bc.** Line *argos*^{24G11}-GAL4 expresses GFP in P domain during S8-9 and 10B, in BC at S10A-13, and in Op at S14. Arrowhead denotes the dorsal midline. Anterior is to the left.

Ca. *broad* (*br*), CG11491

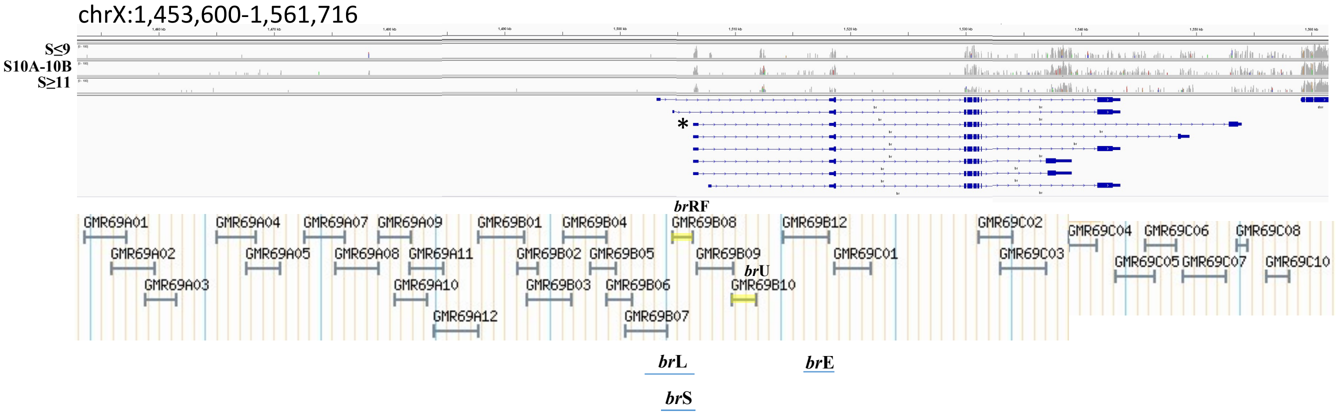

Cb.

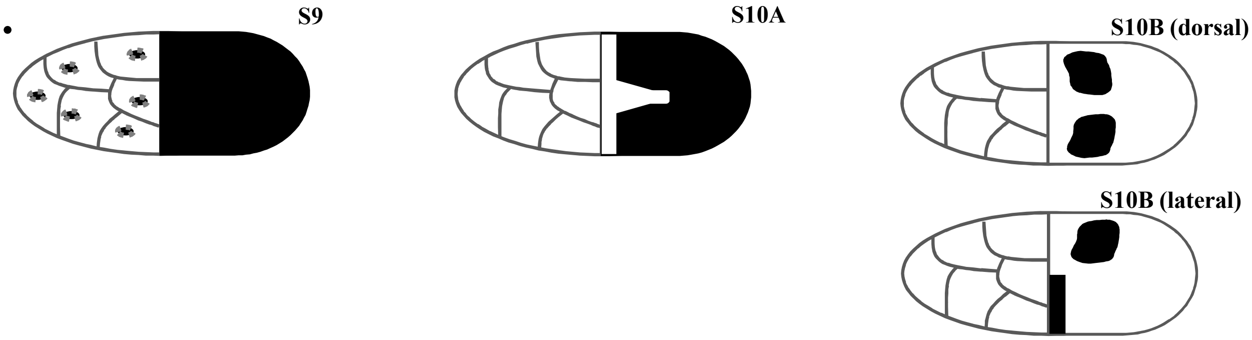

Cc.

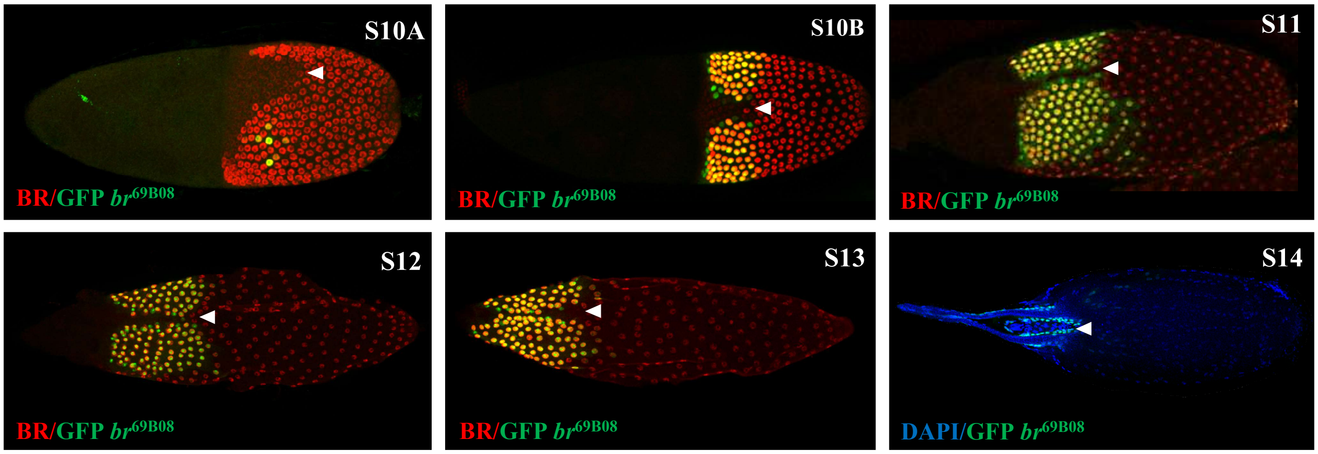

Cd.

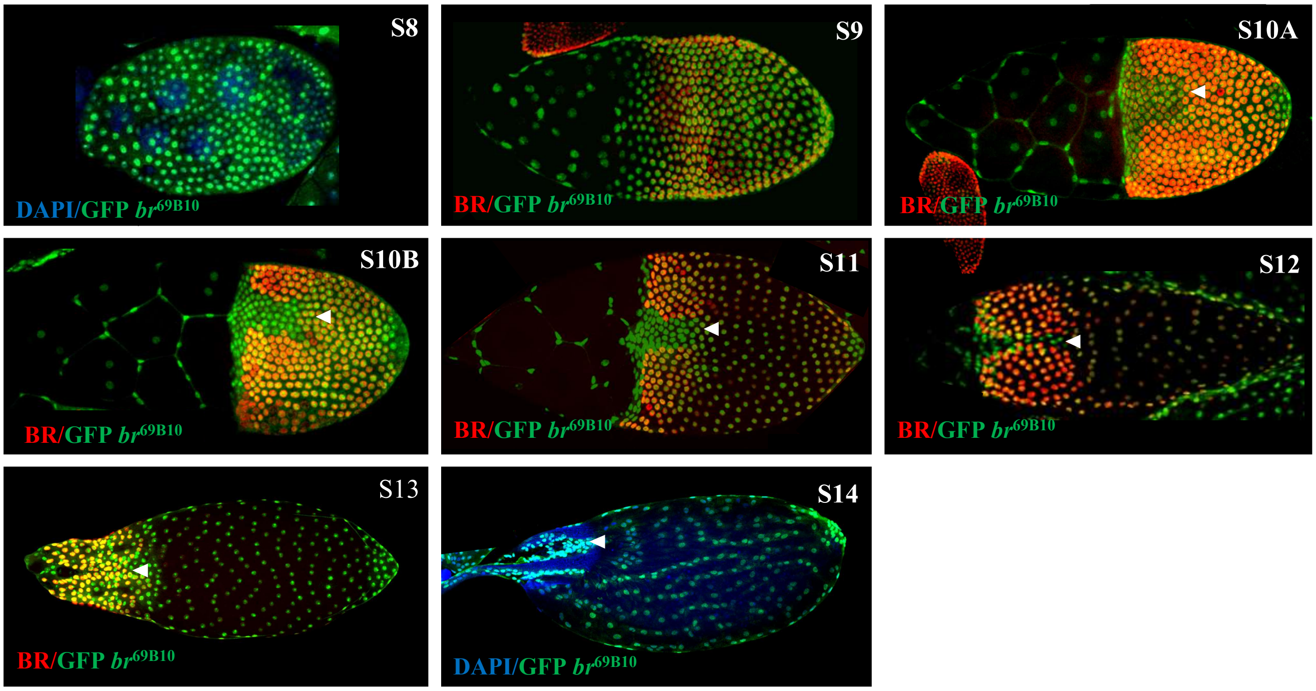

Fig S2 Ca. Gene model for *br* with RNA-seq coverage plot showing isoforms expressed during oogenesis. Asterisk (*) indicates isoform used for mapping analysis. Reads per base range between 0-100 at all developmental stages. Additional fragments of *BrL* and *BrE* (Fuchs et al., 2012) and *BrS* (Charbonnier et al., 2014) are included in model. Fragments mapped to gene model indicate fly lines screened with highlighted boxes representing those expressing GFP. **Cb.** Depiction of *br* expressed in U domain at S9, in U minus M, AD, and SC at S10A, and in the R and AV domain at S10B of oogenesis (Deng and Bownes 1997). **Cc.** Line br^{69B08}-GAL4 is expressed in the R domain at S10A, and R, F domains at S10B-13, and in DA at S14. **Cd.** Line br^{69B10}-GAL4 is expressed in U domain at S8-14. Arrowhead denotes the dorsal midline. Anterior is to the left.

Da. *daughters against dpp* (*dad*), CG5201

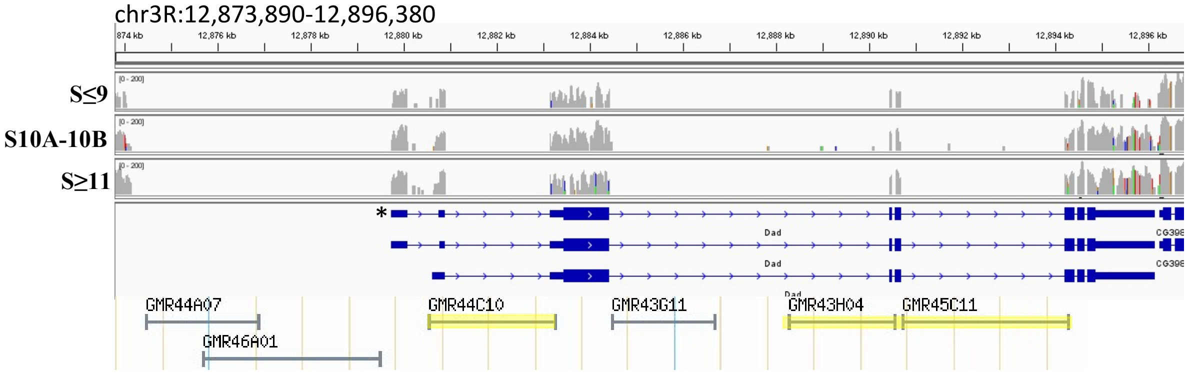

Db.

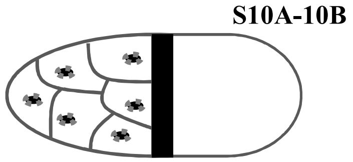

Dc.

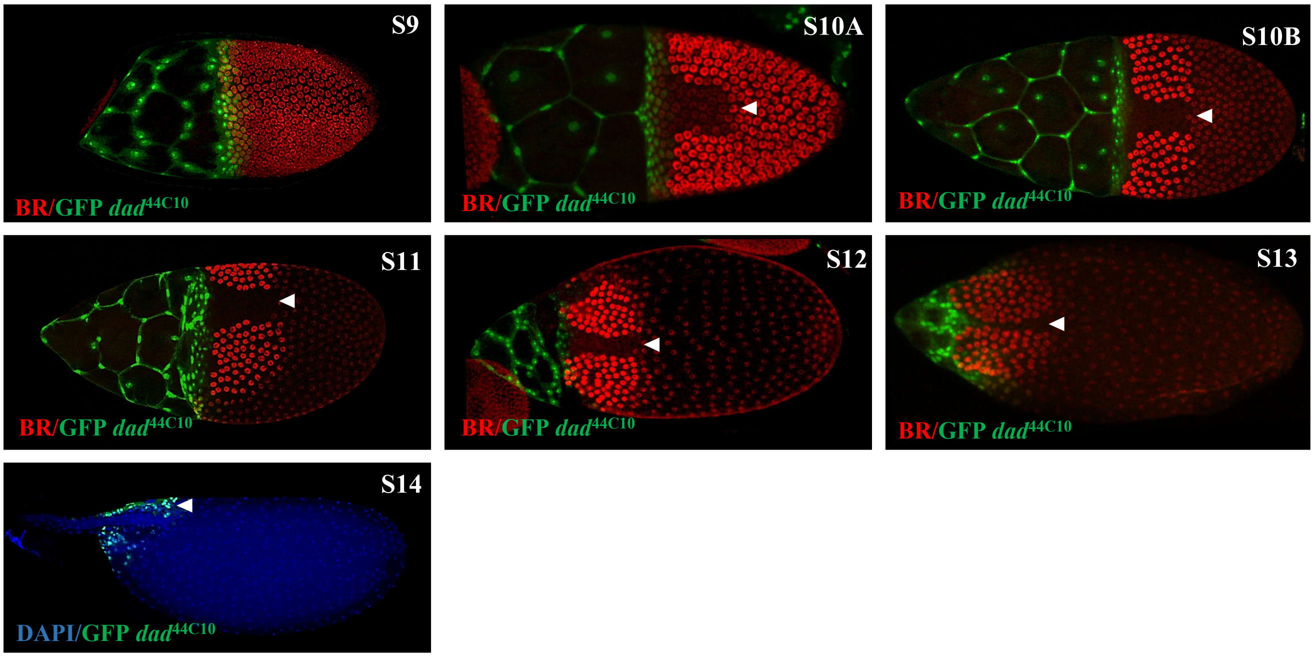

Dd.

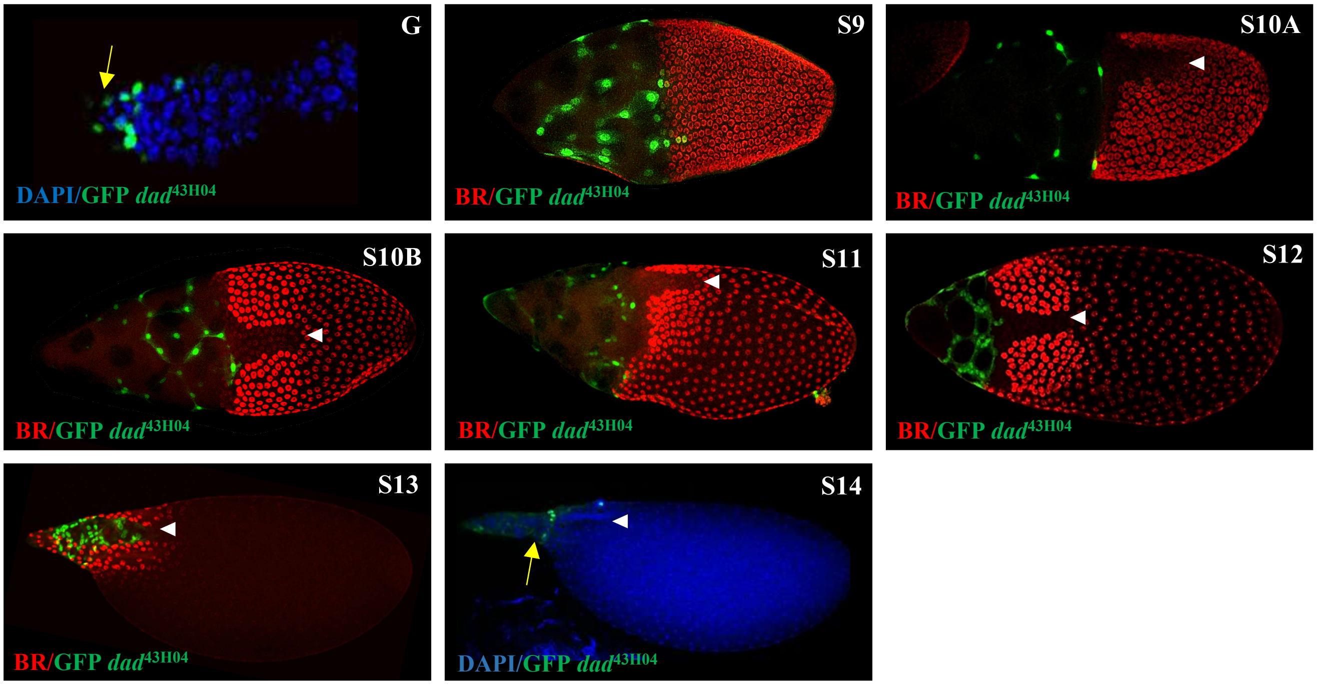

De.

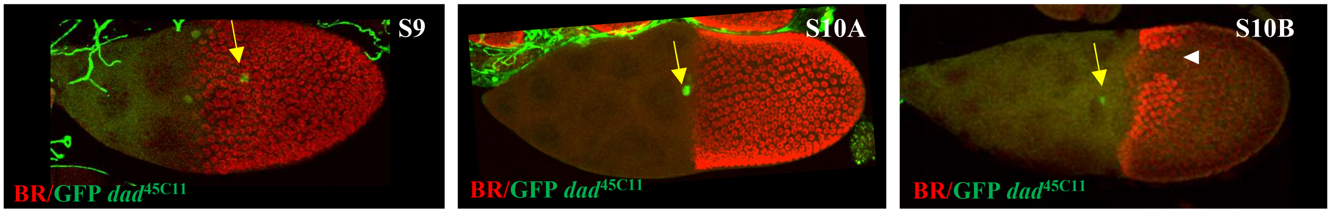

Fig S2 Da. Gene model for *dad* with RNA-seq coverage plot showing active isoforms during oogenesis. Lines mapped below model indicate lines that were screened with highlighted boxes (yellow) representing those that have expression of GFP. Reads per base range between 0-200 at all developmental stages. **Db.** Depiction of endogenous *dad* expression in AD, AV, and SC at S10A-10B of oogenesis (Muzzopappa and Wappner, 2005, Jordan et al. 2005). **Dc.** Line dad^{44C10}-GAL4 is expressed in SC, AD, and AV domains during S8-13, and in the Op at S14. **Dd.** Line dad^{43H04}-GAL4 in G (yellow arrows) and in SC at S9-13, and in the Op at S14. **De.** Line dad^{45C11}-GAL4 is expressed in the BCs (yellow arrow) at S9-10B. In the images at S9 and 10A, GFP is also expressed in trachea. Arrowhead denotes the dorsal midline. Anterior is to the left.

Ea. *division abnormally delayed (dally)*, CG4974

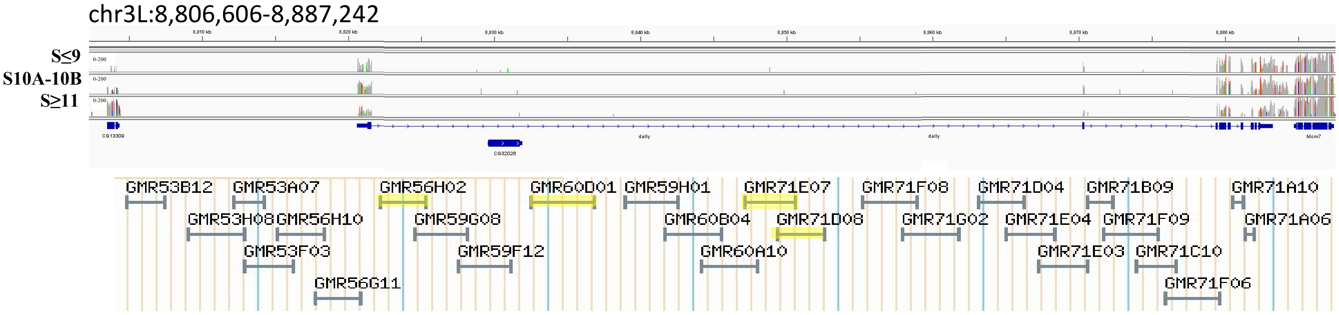

Eb.

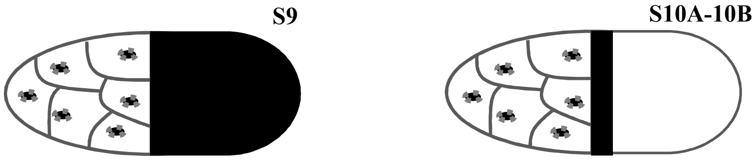

Ec.

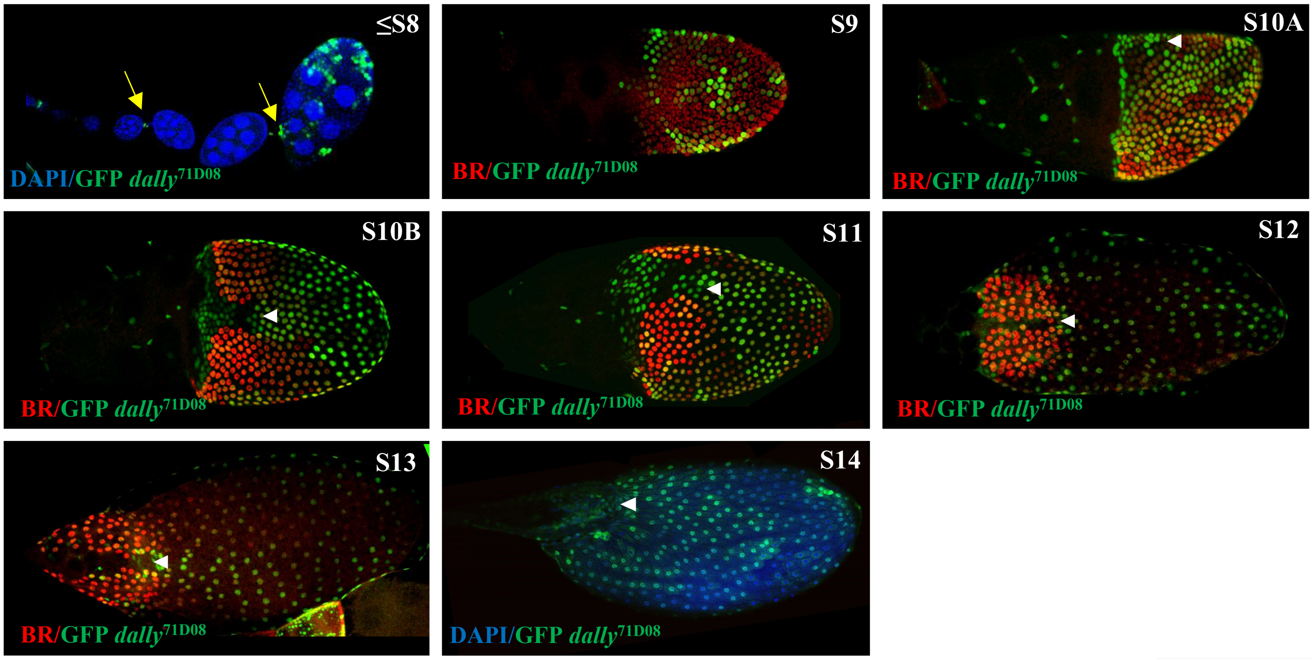

Ed.

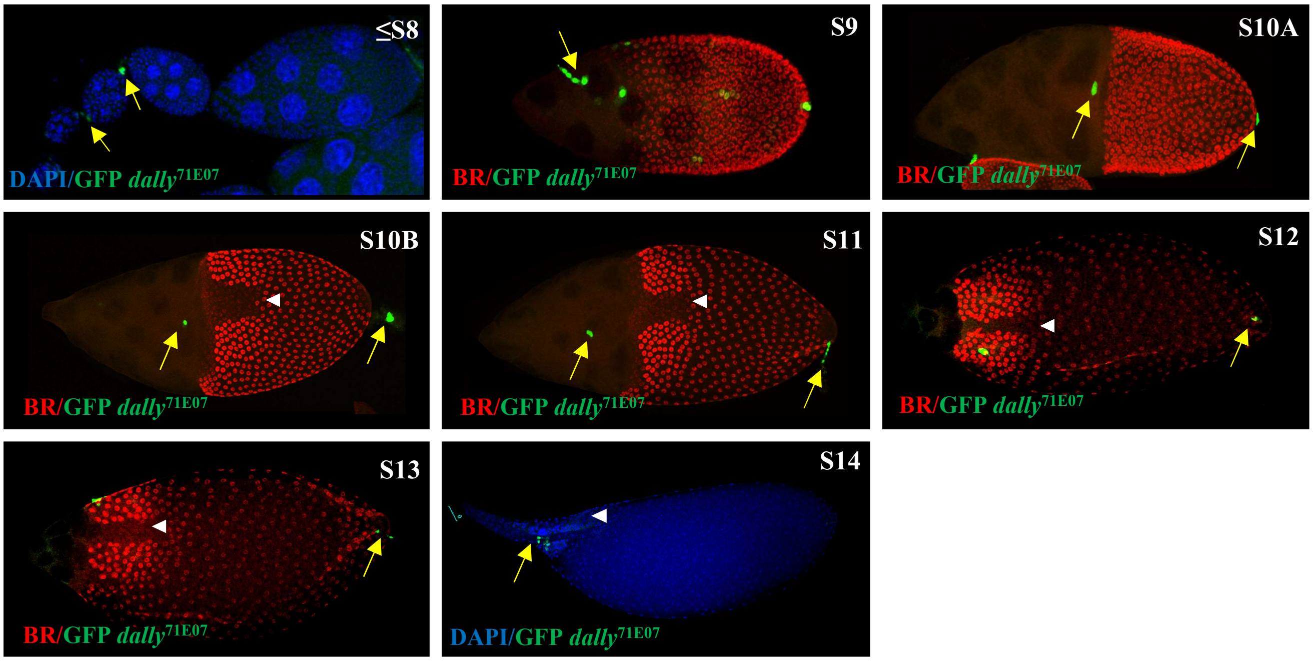

Ee.

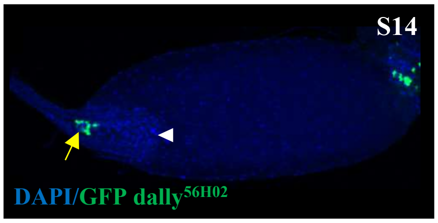

Ef.

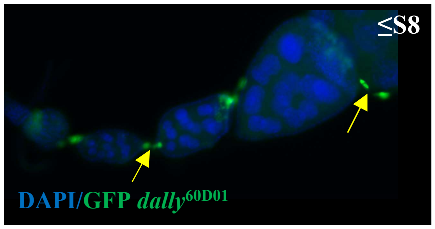

Fig S2 Ea. Gene model for *dally* with RNA-seq coverage plot showing only one isoform is expressed during oogenesis. Fragments mapped below model indicate lines screened with highlighted boxes (yellow) representing those that had expression of GFP. Reads per base range between 0-200 at all developmental stages. **Eb.** Depiction of endogenous *dally* expression U at S9, and SC, AD, and AV at S10A and S10B (Yakoby et al. 2008). **Ec.** Line *dally*^{71D08}-GAL4 expresses GFP in StC during S2-8, and U during S8-14. **Ed.** Line *dally*^{71E07}-GAL4 expresses GFP in the StC (yellow arrow) at S2-14 and in the BC (yellow arrow) during S9-11. **Ee.** Line *dally*^{56H02}-GAL4 expresses GFP in the BCs (yellow arrow) at S14. **Ef.** Line *dally*^{60D01}-GAL4 expresses GFP in StC (yellow arrows) during S2-8. Images for *dally*^{60D01}-GAL4 were taken using a Leica DM2500 compound microscope. Arrowhead denotes the dorsal midline. Anterior is to the left.

Fa. ***decapentaplegic (dpp)*****, CG9885**

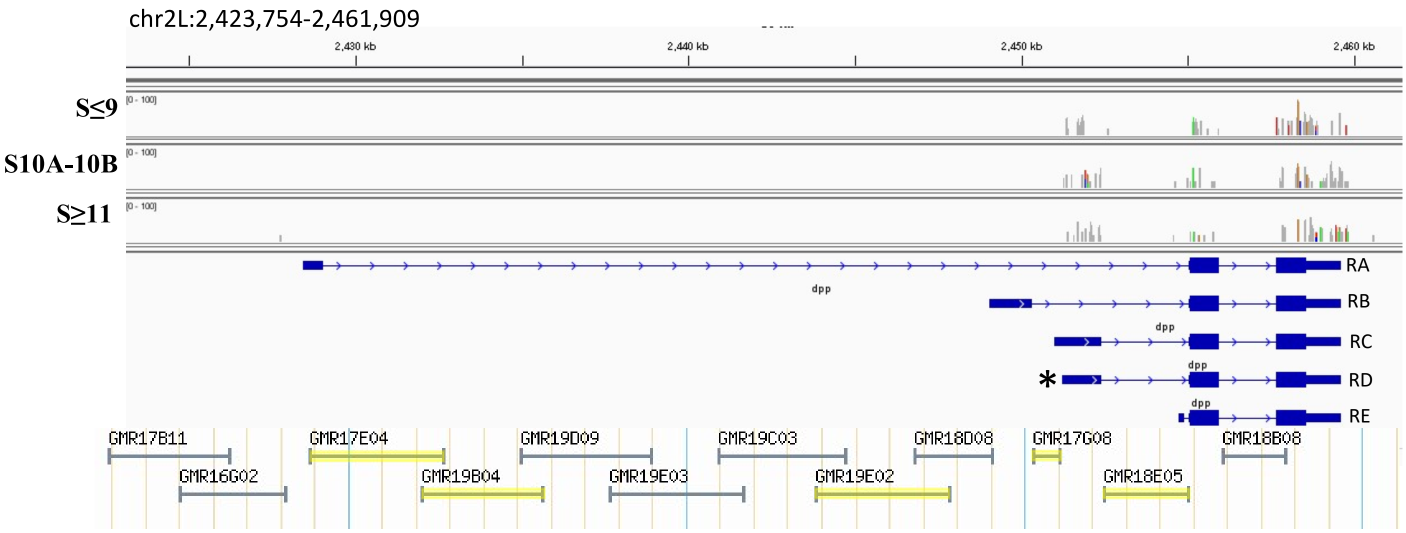

Fb.

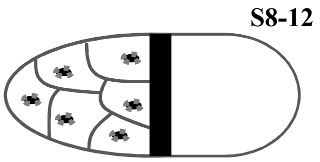

Fc.

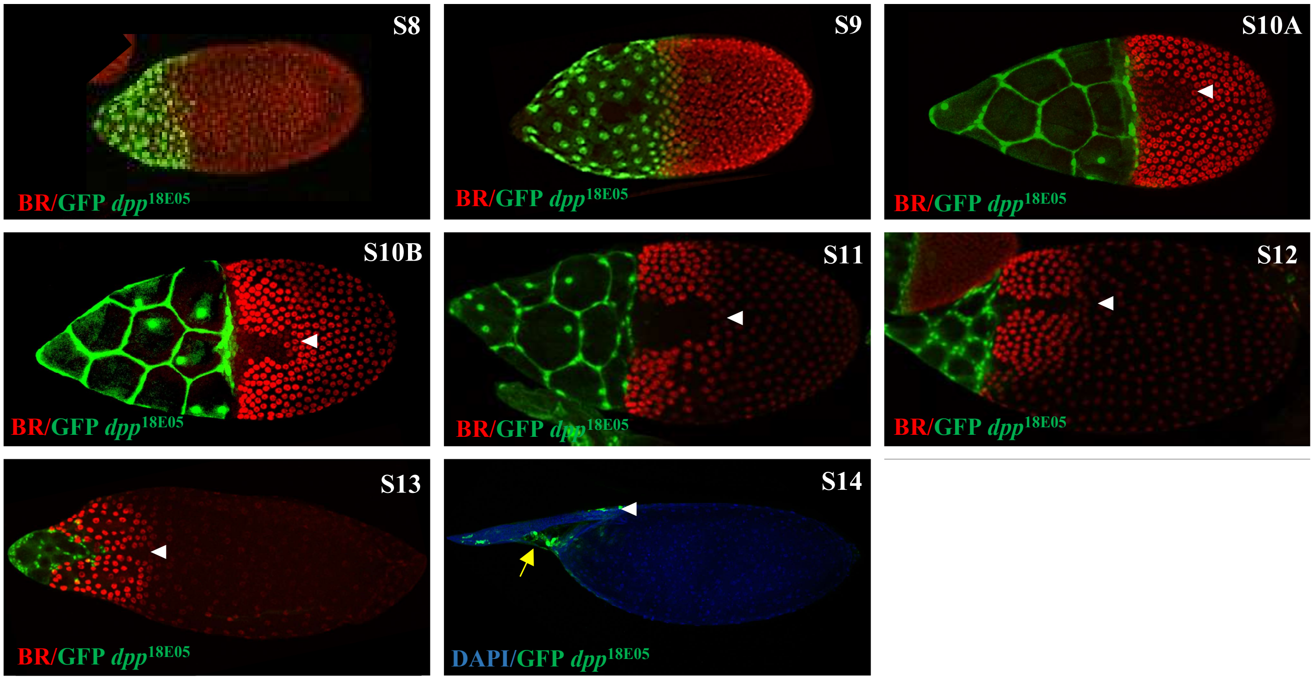

Fd.

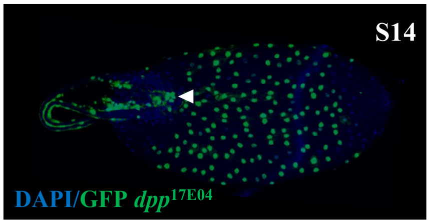

Fe.

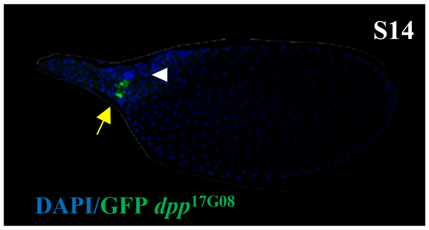

Ff.

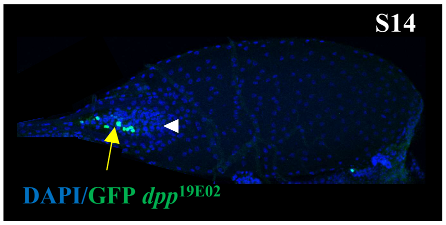

Fg.

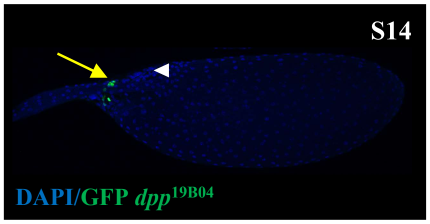

Fig S2 Fa. Gene model for *dpp* with RNA-seq coverage plot showing isoform *dpp*-RD* is expressed during oogenesis. Also *dpp*-RE could be expressed from S10. Lines mapped below model indicate lines that were screened with highlighted boxes (yellow) representing those that had expression of GFP. Reads per base range between 0-100 at all developmental stages. **Fb.** Depiction of endogenous *dpp* expression in SC, AD and AV domains during S8-12 of oogenesis (Twombly et al. 1996). **Fc.** Line *dpp*^{18E05}-GAL4 expresses GFP in SC, AD, and AV during S8-13, and Op at S14. **Fd.** Line *dpp*^{17E04}-GAL4 expresses GFP in U at S14. **Fe.** Line *dpp*^{17G08}-GAL4 expresses GFP in the BC (yellow arrow) at S14. **Ff.** Line *dpp*^{19E02}-GAL4 expresses GFP in Op at S14. **Fg.** Line *dpp*^{19B04} GAL4 expresses GFP in BC (yellow arrow) at S14. Arrowhead denotes the dorsal midline. Anterior is to the left.

Ga. *Ecdysone-induced protein 75B (Eip75B)*, CG8127

chr3L:17,940,428- 18,089,166

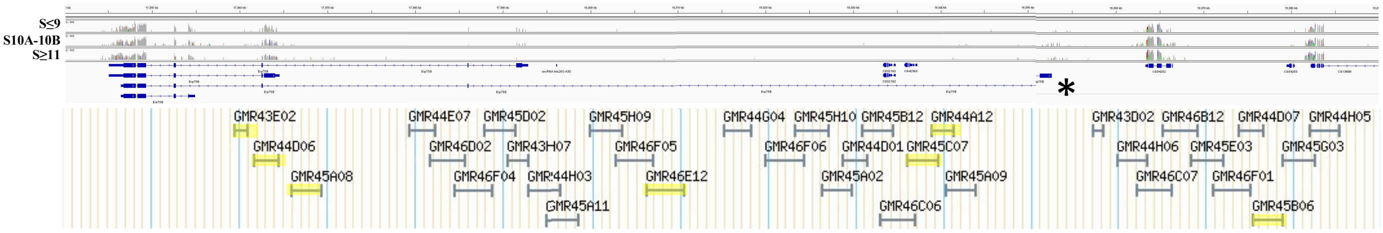

Gb.

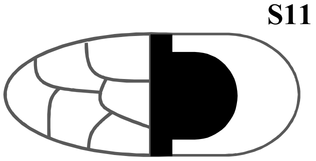

Gc.

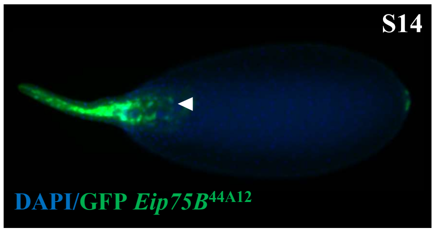

Gd.

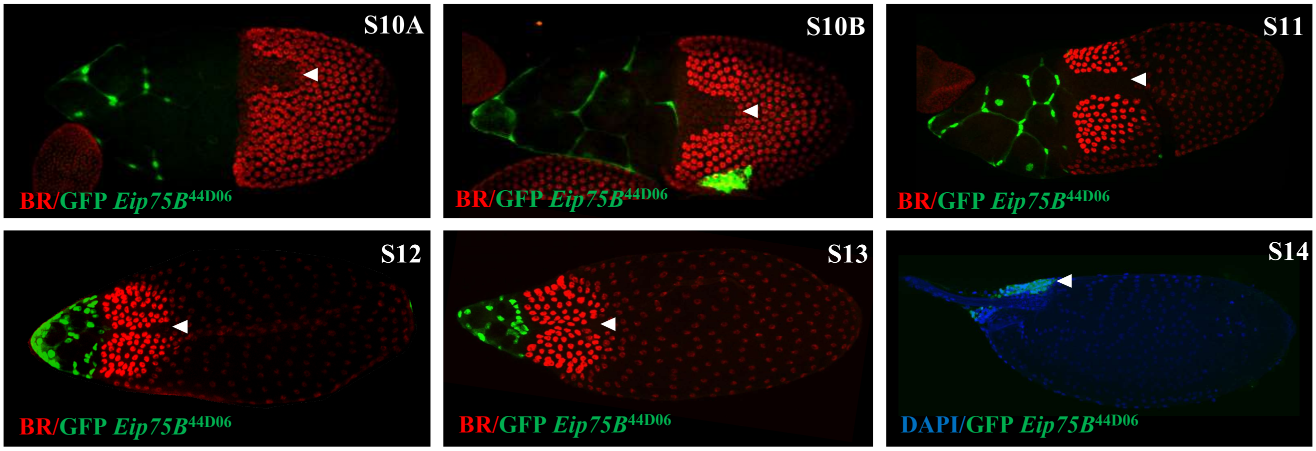

Ge.

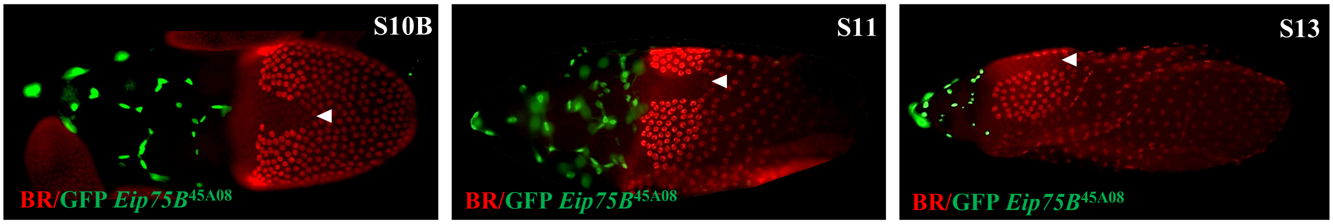

S10B
BR/GFP $Eip75B^{45A08}$
S11
BR/GFP $Eip75B^{45A08}$
S13
BR/GFP $Eip75B^{45A08}$

Gf.

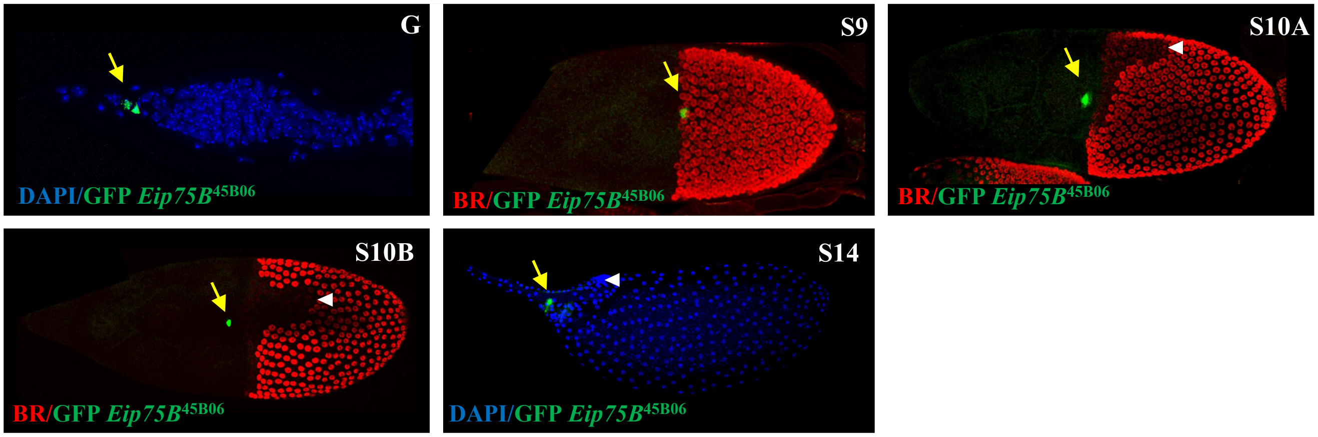

G
DAPI/GFP $Eip75B^{45B06}$
S9
BR/GFP $Eip75B^{45B06}$
S10A
BR/GFP $Eip75B^{45B06}$
S10B
BR/GFP $Eip75B^{45B06}$
S14
DAPI/GFP $Eip75B^{45B06}$

Gg.

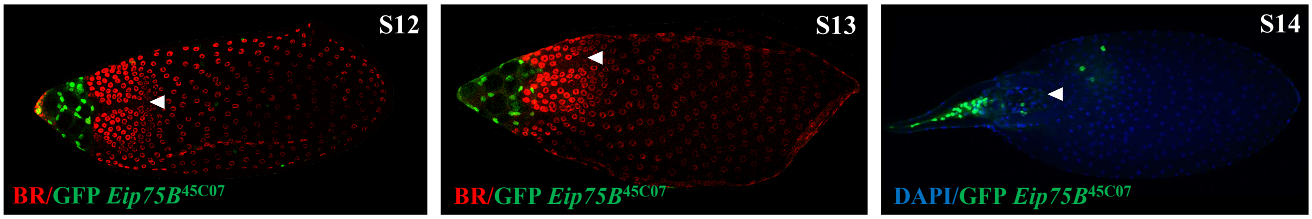

S12
BR/GFP $Eip75B^{45C07}$
S13
BR/GFP $Eip75B^{45C07}$
S14
DAPI/GFP $Eip75B^{45C07}$

Gh.

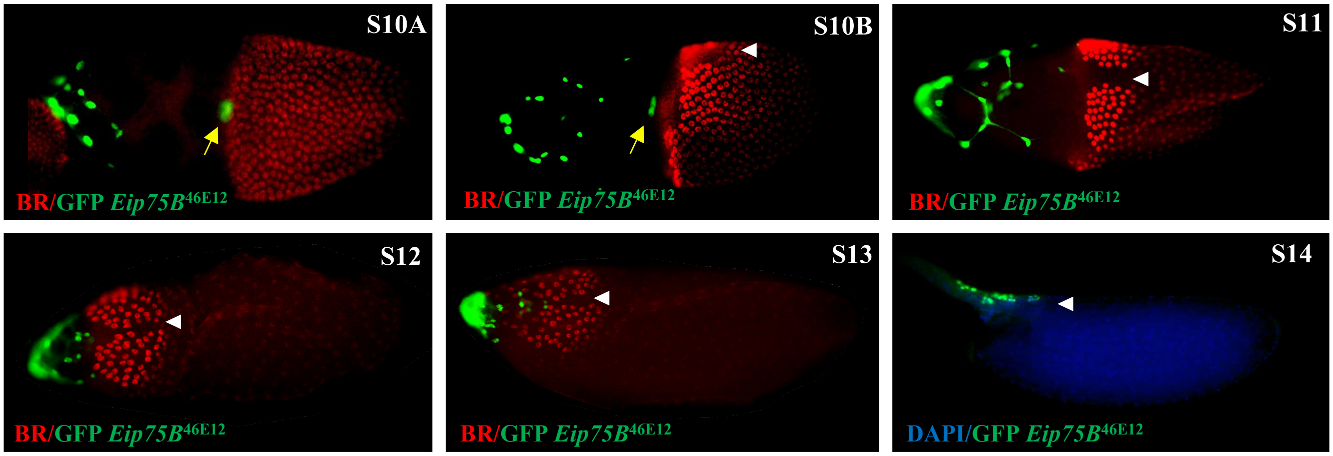

Fig S2 Ga. Gene model for *Eip75B* with RNA-seq coverage plot showing all isoforms expressed. Asterisk (*) indicates isoform used for analysis. Reads per base range between 0-500 for all developmental stages. Fragments mapped below model indicate lines screened with highlighted boxes (yellow) representing those that had expression of GFP. **Gb.** Depiction of endogenous *Eip75B* expression in AD, M, F, R and D at S11 of oogenesis (Buszczak et al. 1999). **Gc.** Line *Eip75B*^{44A12}-GAL4 expresses GFP in the DA at stage 14. **Gd.** Line *Eip75B*^{44D06}-GAL4 expresses GFP in the SC during stages 10A-13, and Op at stage 14. **Ge.** Line *Eip75B*^{45A08} is expressed in the SC at S10B-12. **Gf.** Line *Eip75B*^{45B06} GAL4 expresses GFP in G, and in BC (yellow arrow) at S9-10B and 14. **Gg.** Line *Eip75B*^{45C07}-GAL4 expresses GFP in SC at S12-13, and in the DA at S14. **Gh.** Line *Eip75B*^{46E12}-GAL4 is expressed in the SC from S10A-13 and DA at S14. Arrowhead denotes the dorsal midline. Anterior is to the left. Images for *Eip75C*^{44A12}-GAL4, *Eip75C*^{45A08}-GAL4, and *Eip75C*^{46E12}-GAL4 were taken using a Leica DM2500 compound microscope.

Ha. *Ecdysone-induced protein 78C (Eip78C)*, CG18023

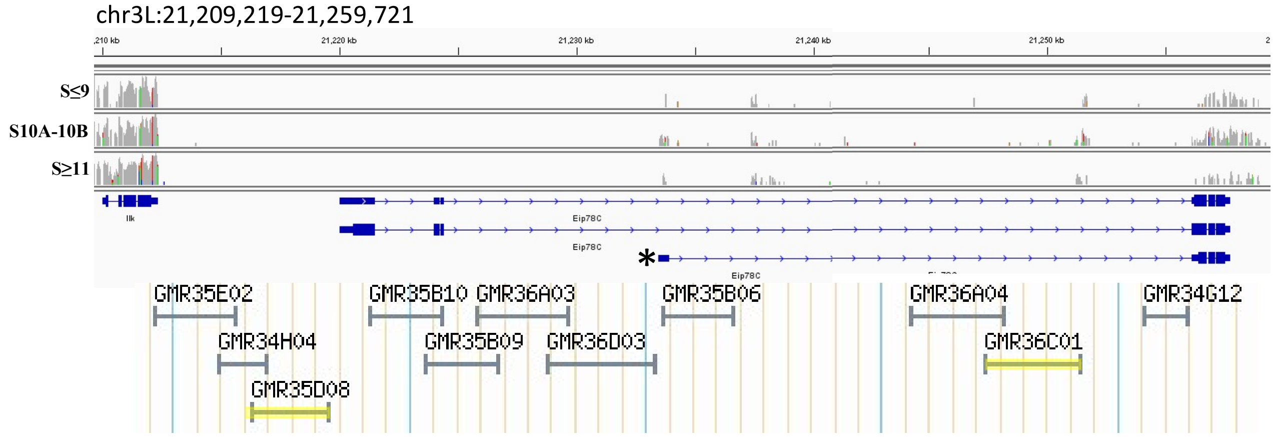

Hb.

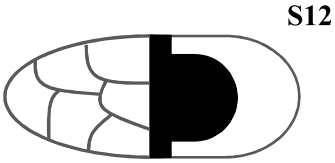

Hc.

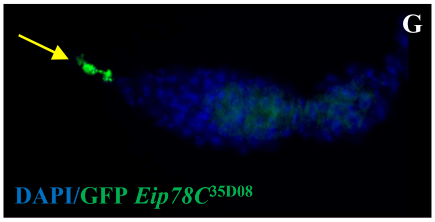

Hd.

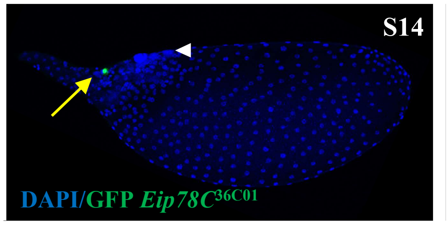

Fig S2 Ha. Gene model for *Eip78C* with RNA-seq coverage plot showing isoform RB* active during oogenesis. Fragments mapped below model indicate lines screened with highlighted boxes (yellow) representing those that had expression of GFP. Reads per base range between 0-300 for all developmental stages. **Hb.** Depiction of endogenous *Eip78C* expression in AD, M, F, R and D at S12 of oogenesis (Bryant et al. 1999). **Hc.** Line *Eip78C*^{35D08}-GAL4 expresses GFP in the G during oogenesis. **Hd.** Line *Eip78C*^{36C01}-GAL4 expresses GFP in BC at S14. Images for *Eip78C*^{35D08}-GAL4 were taken using a Leica DM2500 compound microscope. Arrowhead denotes the dorsal midline. Anterior is to the left.

Ia. *extra macrochaetae* (*emc*), CG1007

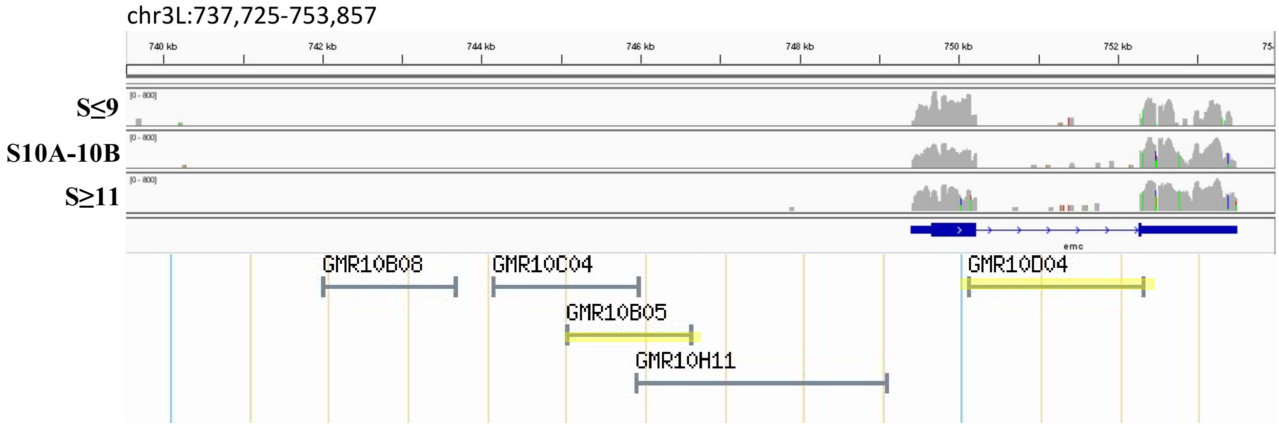

Ib.

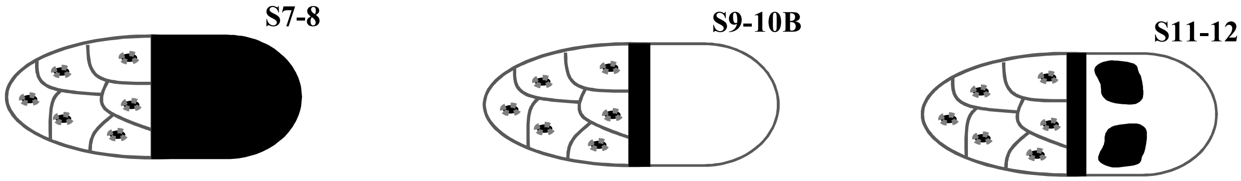

Ic.

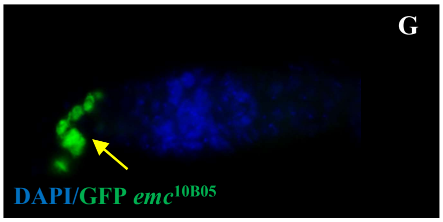

Id.

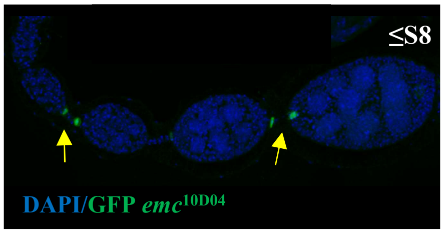

Fig S2 Ia. Gene model for *emc* with RNA-seq coverage plot showing only one isoform is expressed during oogenesis. Fragments mapped below model indicate lines screened with highlighted boxes representing those that had expression of GFP. Reads per base range between 0-800 for all developmental stages. **Ib.** Depiction of endogenous *emc* expression U at S7-8, SC, AD, and AV during S9-10B, and in AD, AV, SC, and R at S11-12 (Papadia et al. 2005). These cartoon are in dorsal views, thus the AV domain is not seen. **Ic.** Line *emc*^{10B05}-GAL4 expressing GFP in the G (yellow arrow). **Id.** Line *emc*^{10D04}-GAL4 in the StC (yellow arrows) at S2-8. Images for *emc*^{10B05}-GAL4 were taken using a Leica DM2500 compound microscope. Anterior is to the left.

Ja. *fasciclin II (fas II)*, CG3665

chrX:4,014,410-4,106,823

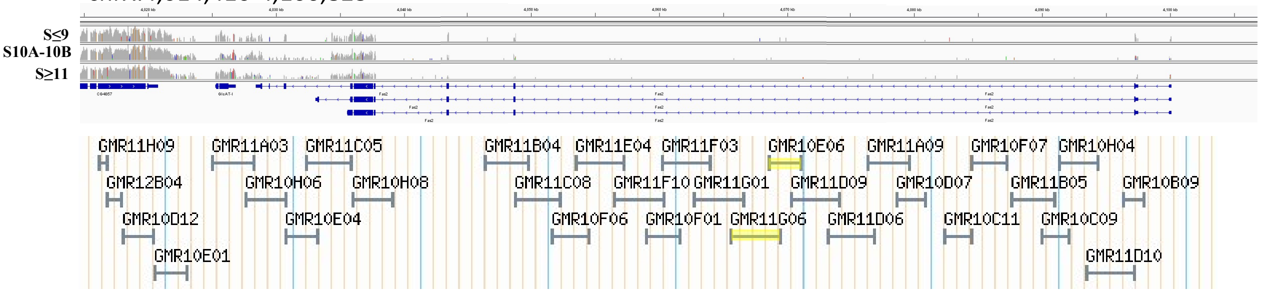

Jb.

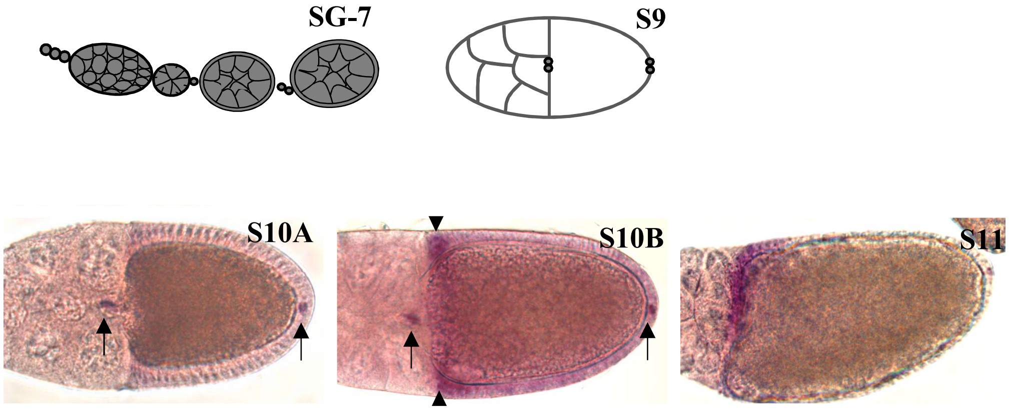

Jc.

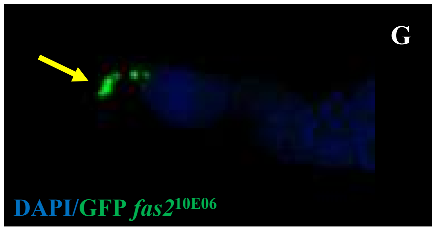

Jd.

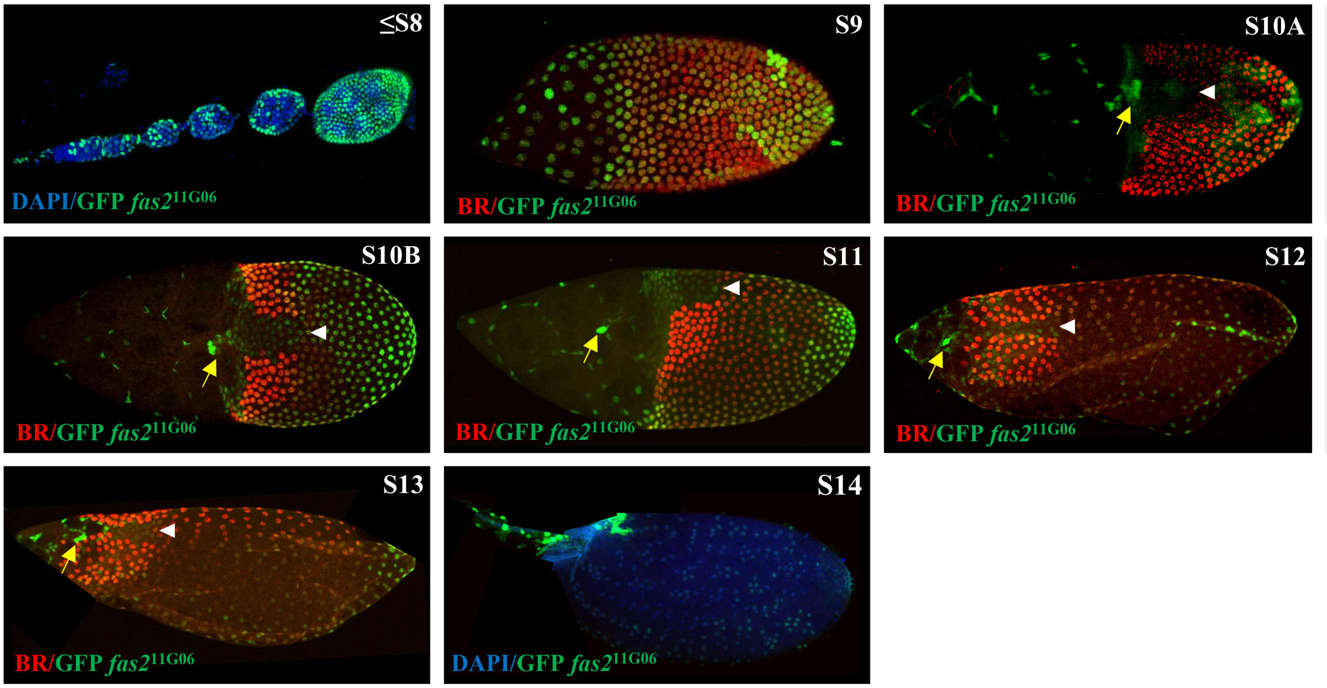

Fig S2 Ja. Gene model for *fasII* with RNA-seq coverage plot showing no discrepancies between isoforms expressed during oogenesis. Fragments mapped below model indicate those screened with highlighted boxes representing those that had expression of GFP. Reads per base range between 0-800 for all developmental stages. **Jb.** Depiction of endogenous *fasII* expression in the G. Also in the U at S1-7, and PC at S9, as previously described (Szafranski and Goode 2004). In addition, in this study we found that *fasII* is expressed in the U and PC (yellow arrows) at S10A, U, AD, AV at S10B, and AD, AV at S11. **Jc.** Line *fasII*^{10E06}-GAL4 expresses GFP in the G (yellow arrow). **Jd.** Line *fasII*^{11G06}-GAL4 expresses GFP in G, and U at S1-14. Arrowhead denotes the dorsal midline. Anterior is to the left.

Ka. *H15*, CG6604

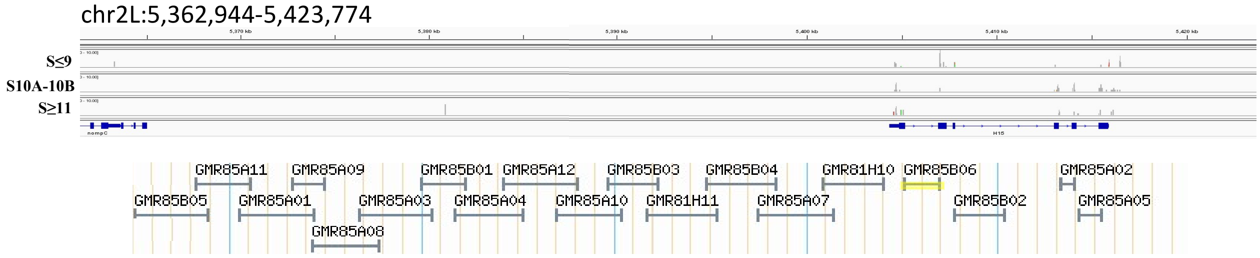

Kb.

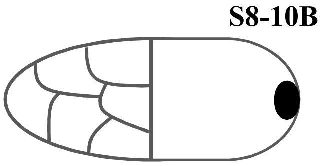

Kc.

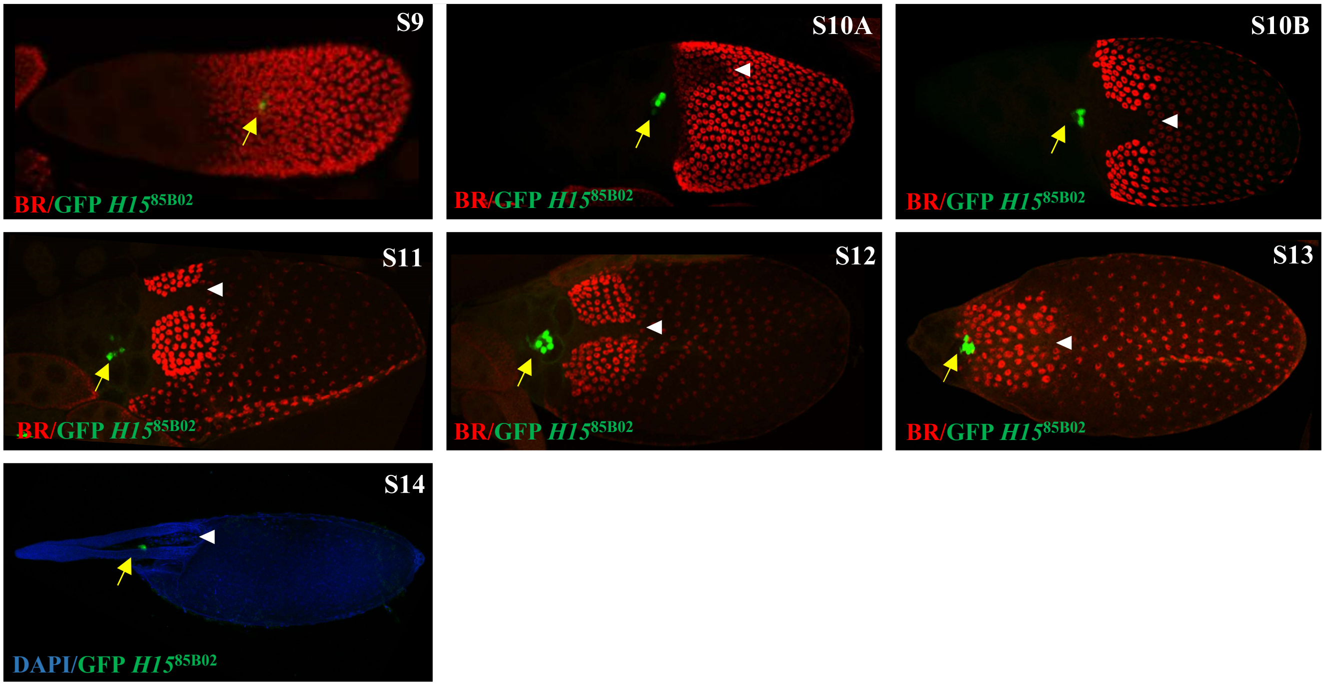

Fig S2 Ka. Gene model for *H15* with RNA-seq coverage plot showing one expressed isoform during oogenesis. Fragments mapped below model indicate lines that were screened with highlighted boxes (yellow) representing those that had expression of GFP. Reads per base range between 0-10 for all developmental stages. **Kb.** Depiction of endogenous *H15* expression in P during S8-10B of oogenesis (Fregoso Lomas et al. 2013). **Kc.** Line *H15*^{85B02}-GAL4 expresses GFP in the BCs (yellow arrows) at S9-14. Arrowhead denotes the dorsal midline. Anterior is to the left.

La. *jaguar (jar)*, CG5695

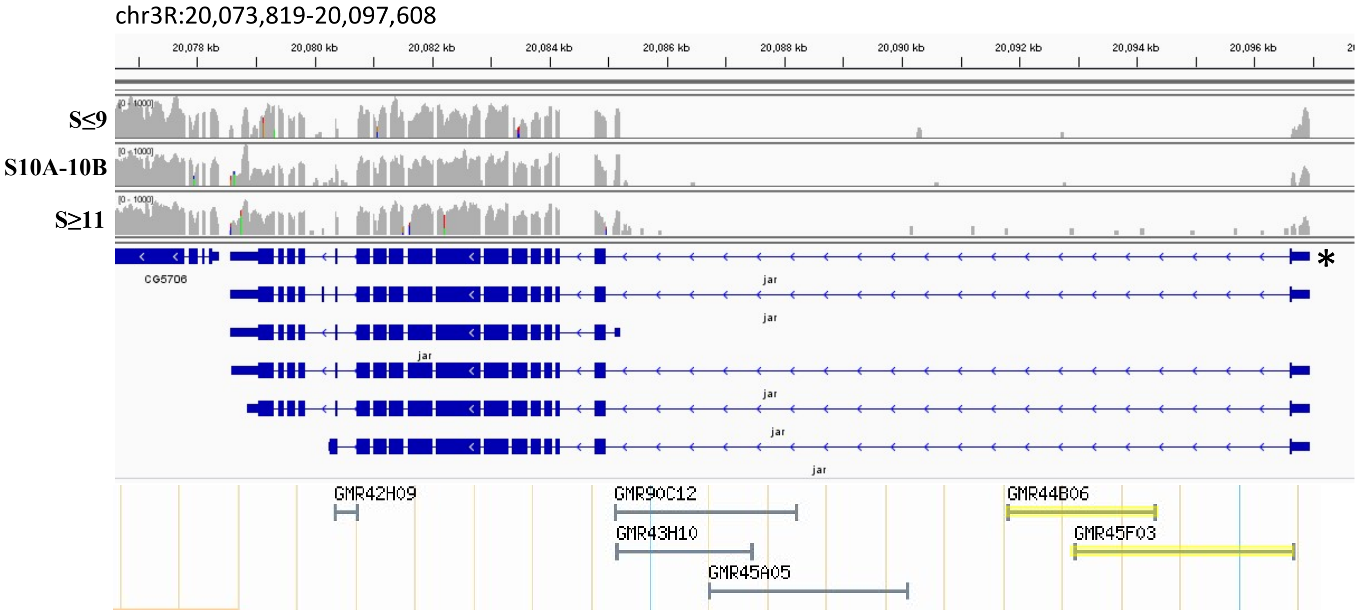

Lb.

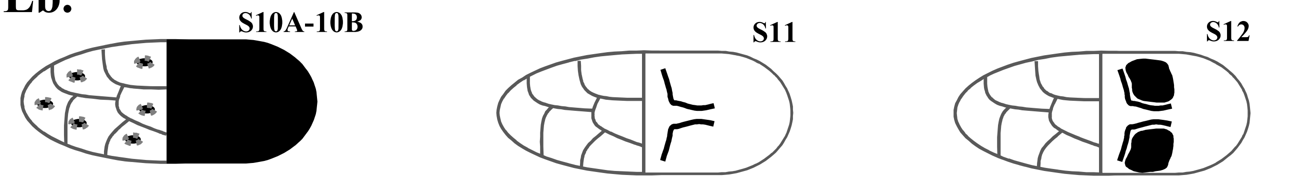

Lc.

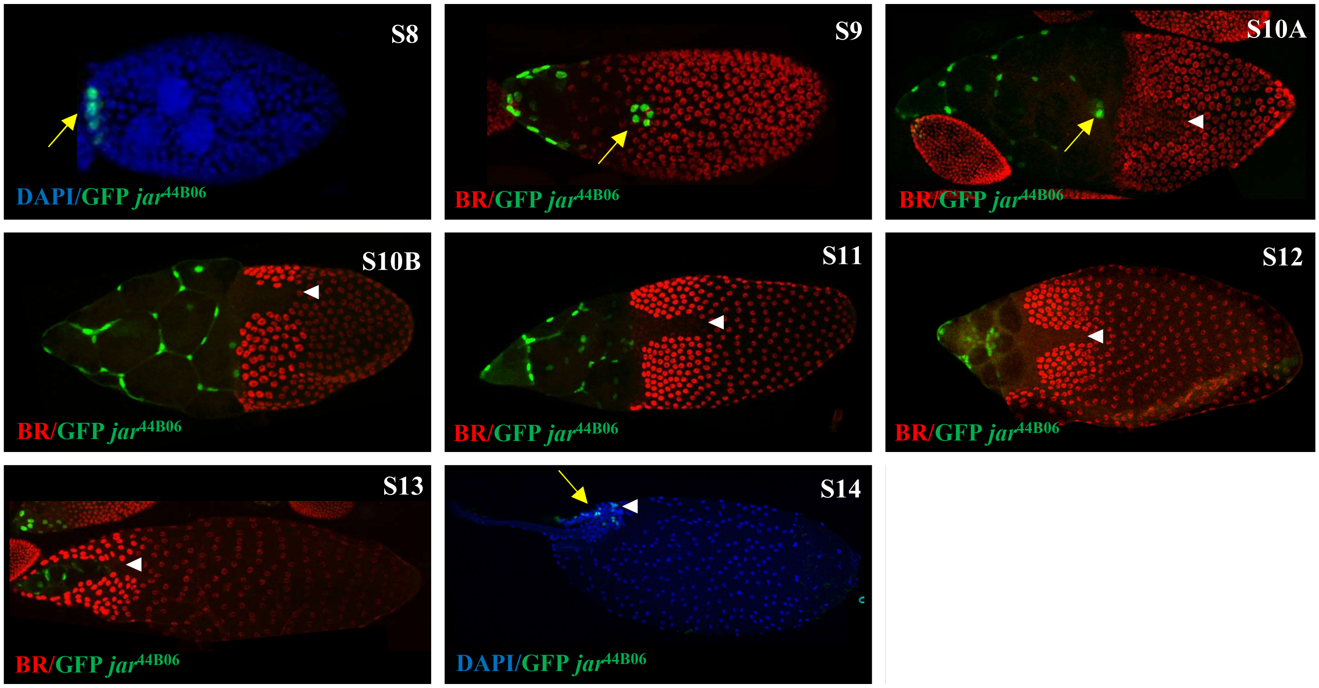

Ld.

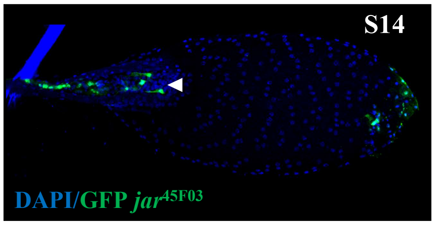

Fig S2 La. Gene model for *jar* with RNA-seq coverage plot showing isoform RJ* expressed during oogenesis. Fragments mapped below model indicate lines that were screened with highlighted boxes (yellow) representing those that had expression of GFP. Reads per base range between 0-1000 for all developmental stages. **Lb.** Depiction of endogenous *jar* expression in U at S10A-10B, F at S11, and R and F at S12 (Deng et al. 1999). **Lc.** Line *jar*$^{44B06-}$GAL4 expresses GFP in SC at S8-14 and BC at S9-10A. We note that the GFP pattern at S8 could also mark the future BC, however, the expression pattern was annotated as future SC (SC). **Ld.** Line *jar*^{45F03}-GAL4 expresses GFP in DA and P at S14. Arrowhead denotes the dorsal midline. Anterior is to the left.

Ma. *jim, CG11352*

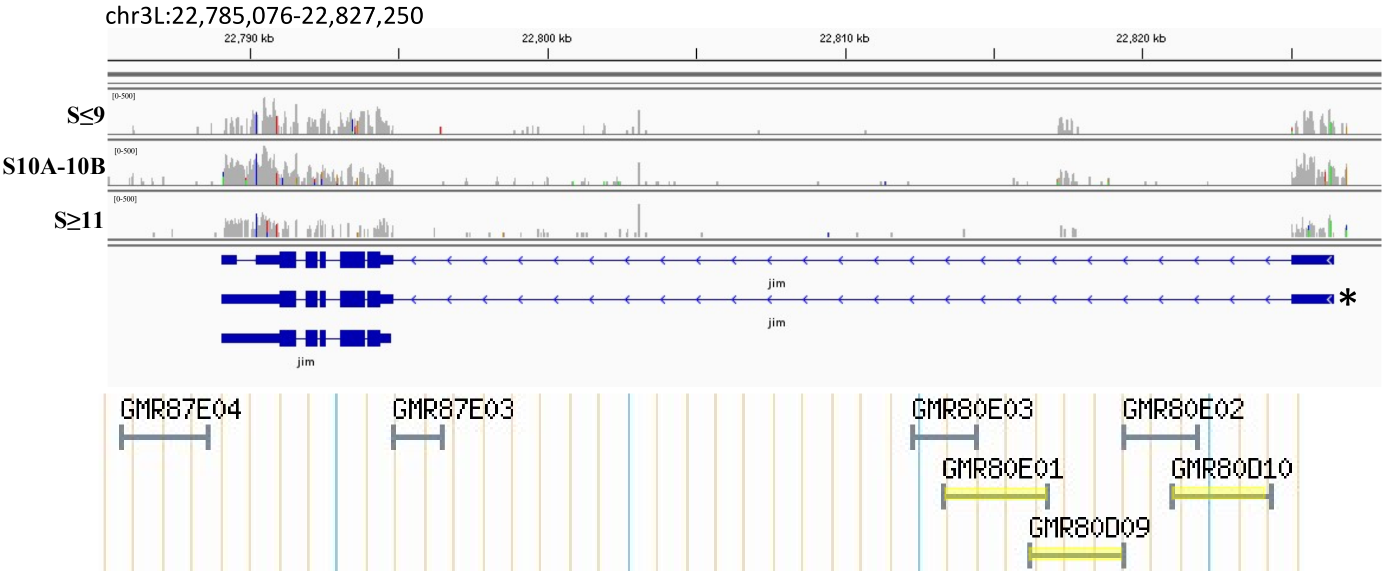

Mb.

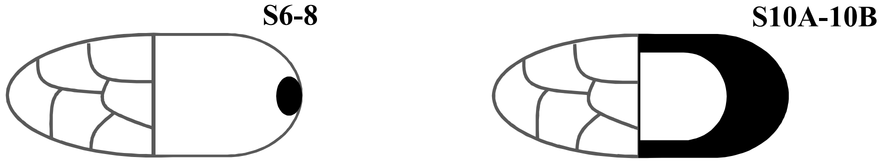

Mc.

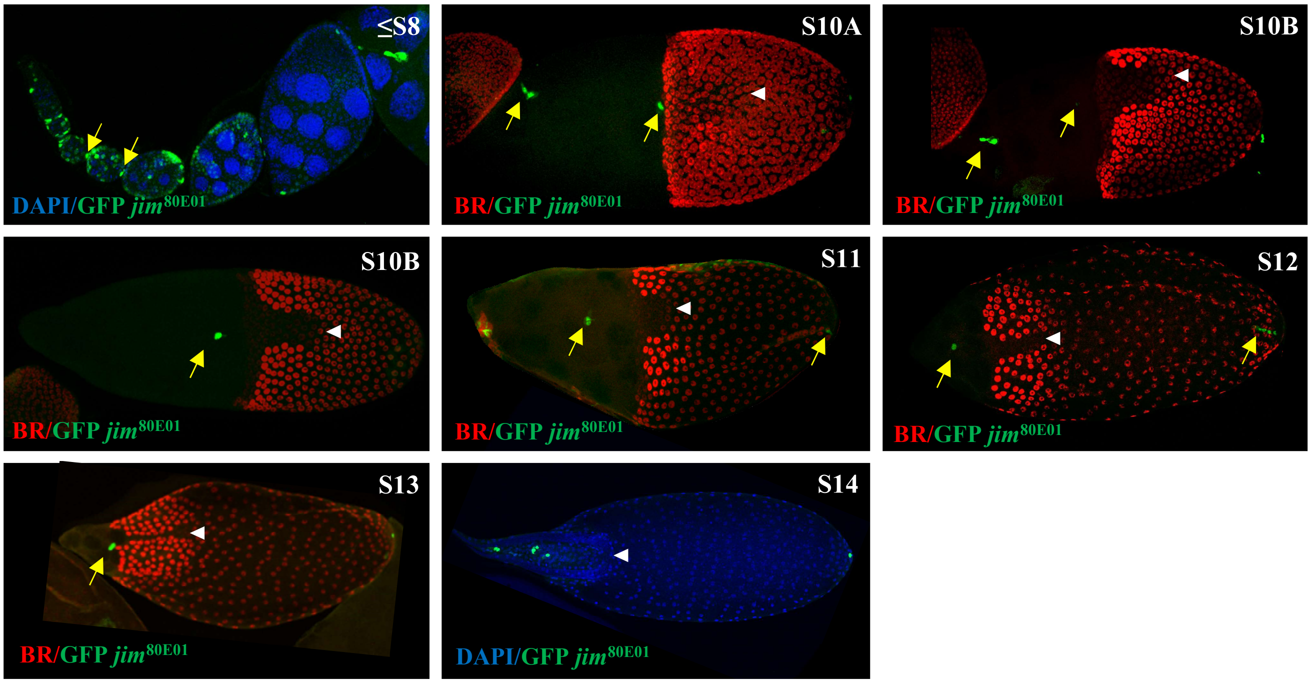

Md.

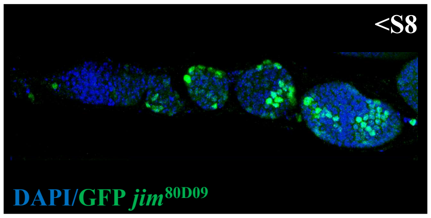

Me.

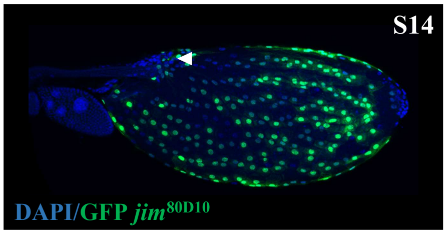

Fig S2 Ma. Gene model for *jim* with RNA-seq coverage plot showing isoforms expressed during oogenesis. Asterisk (*) indicates isoform used for mapping analysis. Fragments mapped below model indicate lines screened with highlighted boxes (yellow) representing those that had expression of GFP. Reads per base range from 0-500 for all developmental stages. **Mb.** Depiction of endogenous *jim* expression in P at S6-8 and U minus SC, AD, M, R, F, and D at S10A and S10B (Doerflinger et al. 1999). **Mc.** Line *jim*^{80E01}-GAL4 expressed in U and StC domains at S2-8 and in the StC at S10A-10B and BCs (yellow arrows) at S10A-14. **Md.** Line *jim*^{80D09}-GAL4 expresses GFP in the U domain at S2-7. **Me.** Line *jim*^{80D10}-GAL4 expresses GFP in the U domain at S14. Arrowhead denotes the dorsal midline. Anterior is to the left.

Na. ***kayak (kay)*, CG33956**

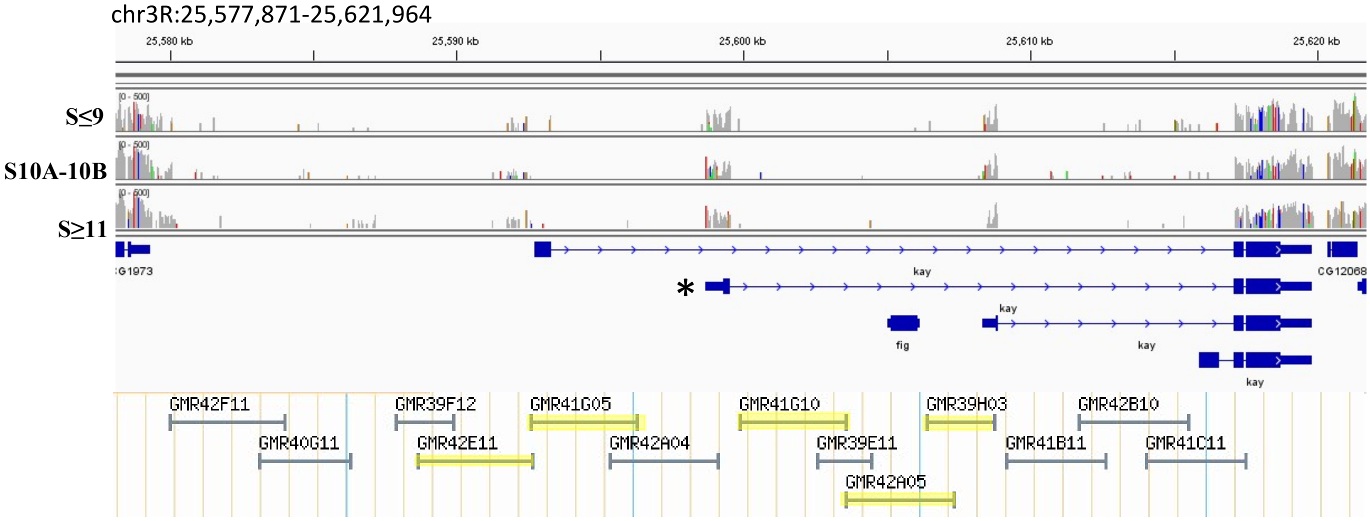

Nb.

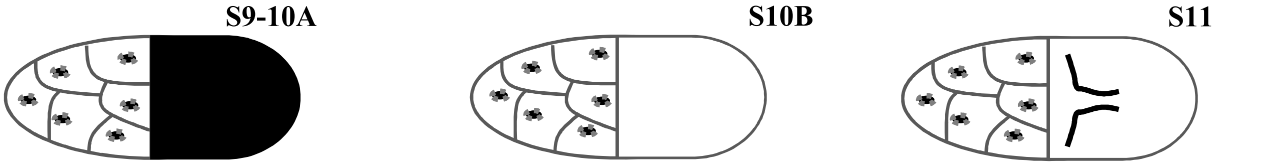

Nc.

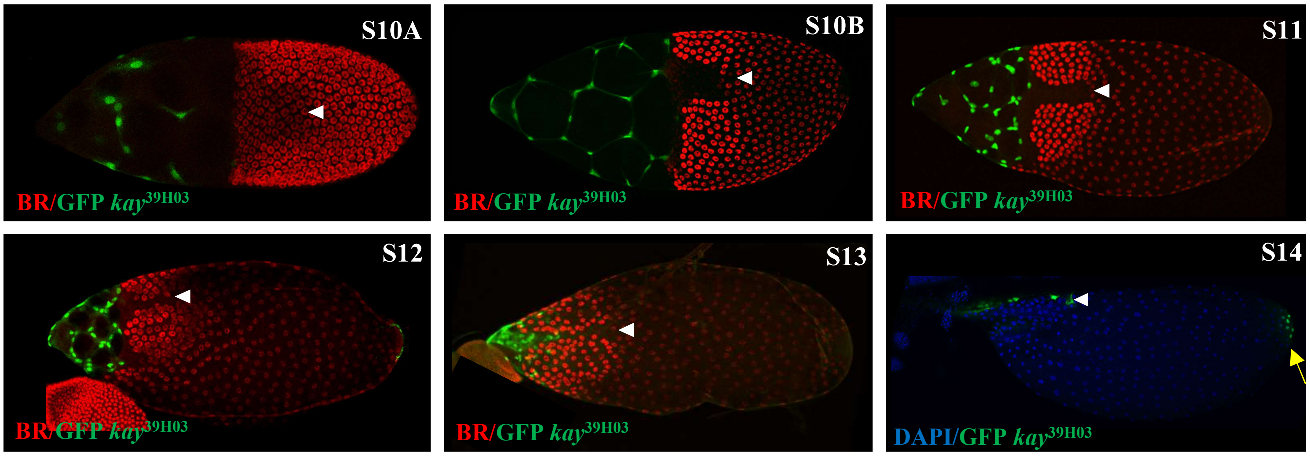

Nd.

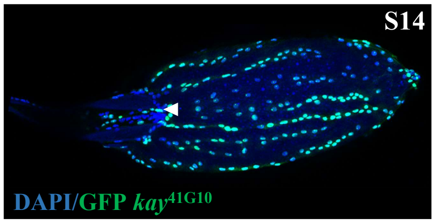

Ne.

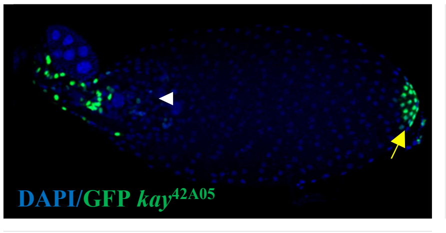

Nf.

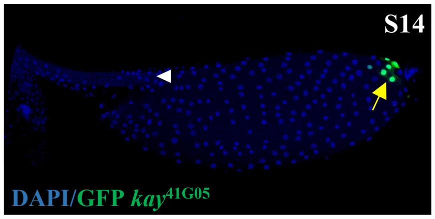

Ng.

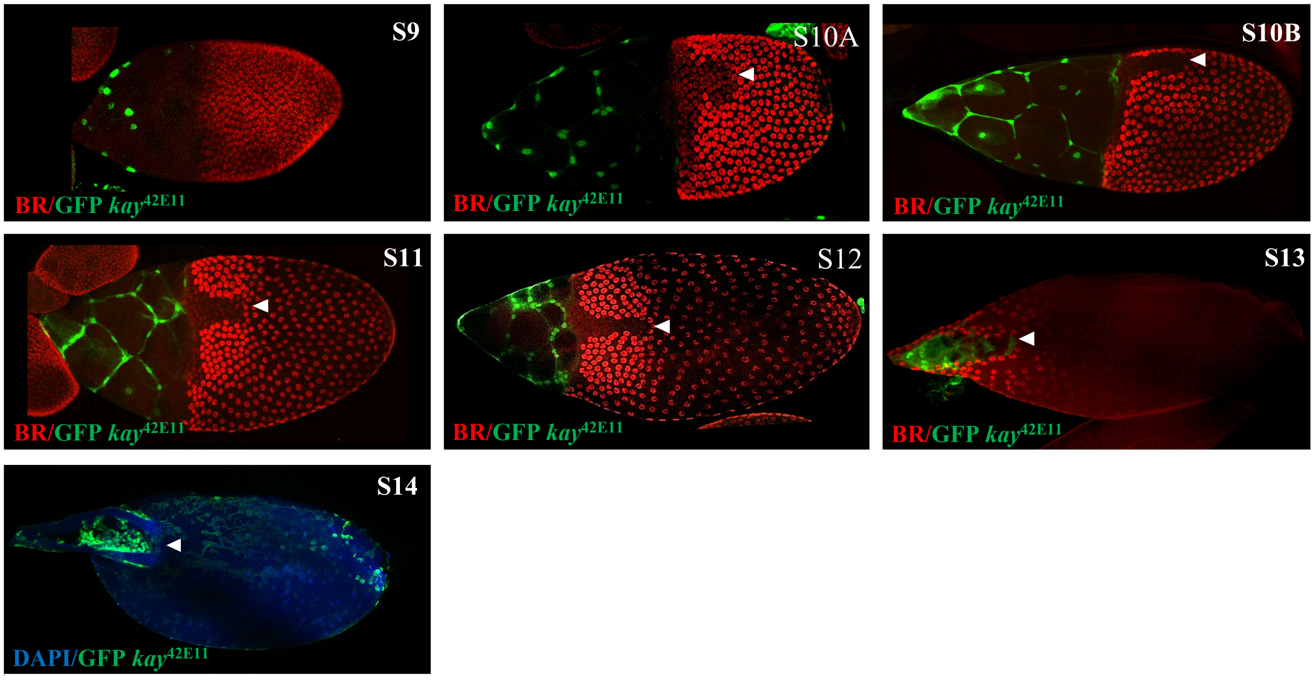

Fig S2 Na. Gene model for *kay* with RNA-seq coverage plot showing active isoforms during oogenesis. Asterisk (*) indicates isoform used for mapping analysis. Fragments mapped below model indicate lines screened with highlighted boxes (yellow) representing those that had expression of GFP. Reads per base range from 0-500 for all developmental stages. **Nb.** Depiction of endogenous *kay* expression in U at S9-10A, SC at S10B, and F and SC at S11 (Dequier et al. 2001). **Nc.** Line kay^{39H03}-GAL4 expresses GFP in SC domain at S10A-13 and Op and P at S14. **Nd.** Line kay^{41G10}-GAL4 expresses GFP in U domain at S14. **Ne.** Line kay^{42A05}-GAL4 expresses GFP in the dorsal appendages and P at S14. **Nf.** Line kay^{41G05}-GAL4 expresses GFP in the P domain at S14. **Ng.** Line kay^{42E11}-GAL4 expresses GFP in the SC at S9-13 and U at S14. Arrowhead denotes the dorsal midline. Anterior is to the left.

Oa. *Lin29, datilografo (dati)*, CG2052

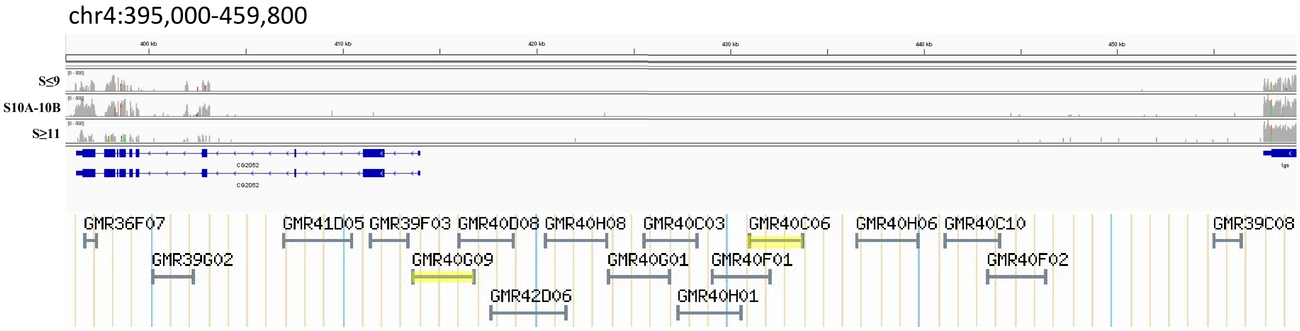

Ob.

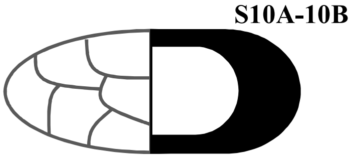

Oc.

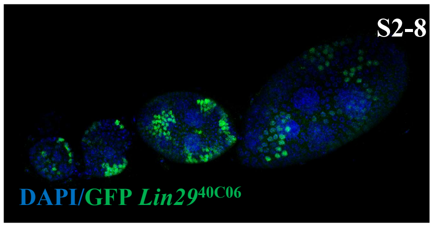

Od.

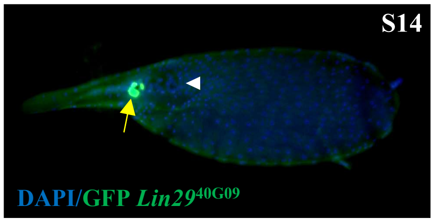

Fig S2 Oa. Gene model for *Lin29* with RNA-seq coverage plot showing only one isoform is expressed during oogenesis. Fragments mapped below model indicate fly lines screened with highlighted boxes (yellow) representing those that had expression of GFP. Reads per base range between 0-500 for all developmental stages. **Ob.** Depiction of endogenous *Lin29* expression in the U domain minus SC, AD, M, R, F, D domains during S10A-10B of oogenesis (Yakoby et al. 2008). **Oc.** Line *Lin29*^{40C06}-GAL4 in the U domain at S2-8. **Od.** Line *Lin29*^{40G09}-GAL4 in the BC domain (yellow arrow) at S14. Images for *Lin29*^{40G09}-GAL4 were taken using a Leica DM2500 compound microscope. Arrowhead denotes the dorsal midline. Anterior is to the left.

Pa. *midline (mid)*, CG6634

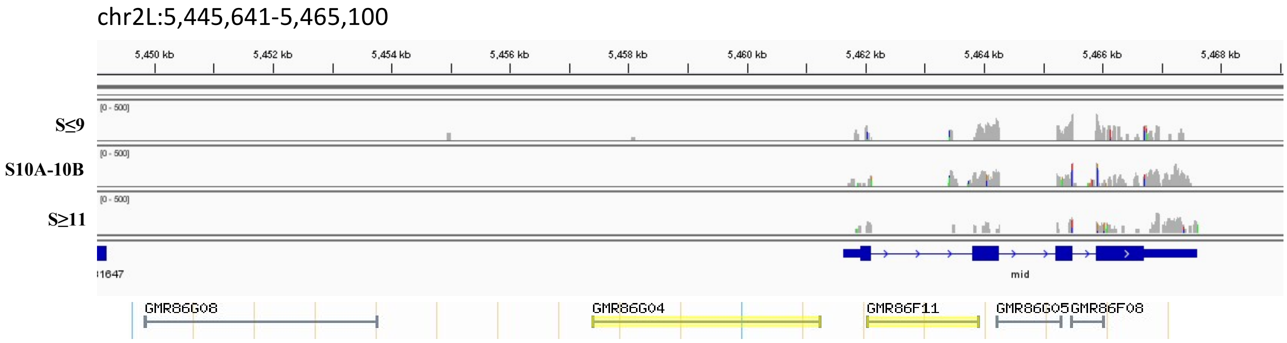

Pb.

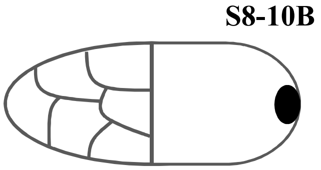

Pc.

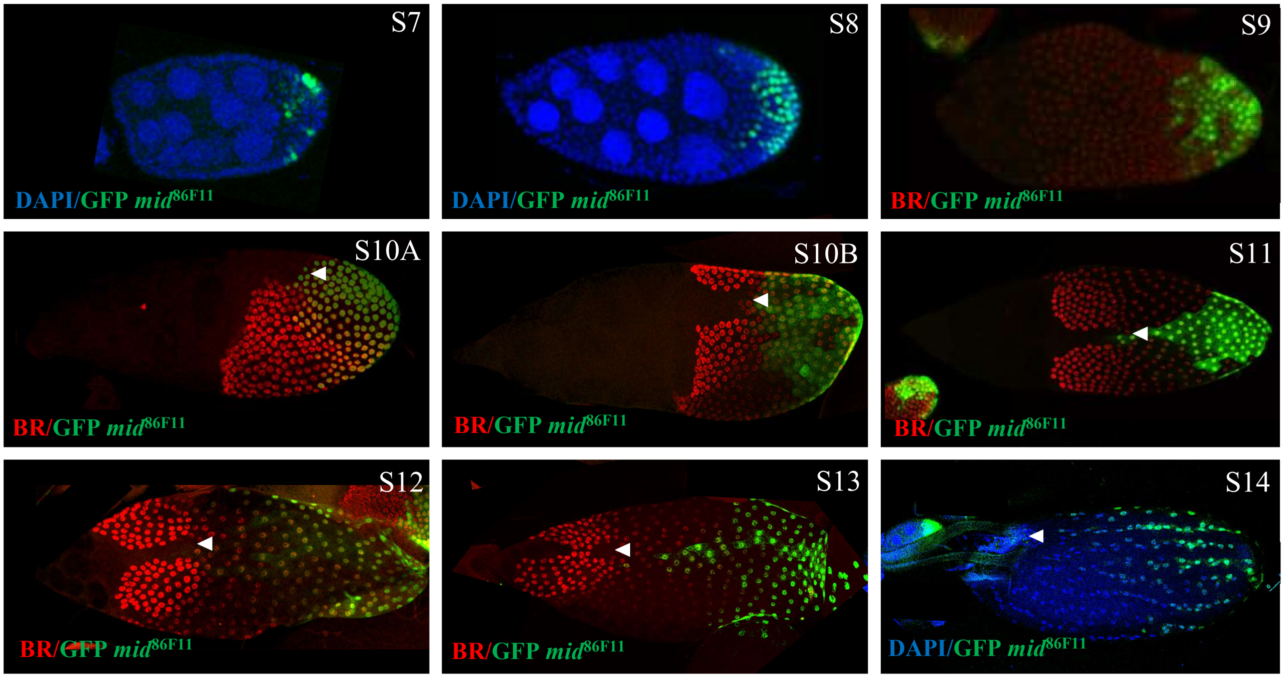

Pd.

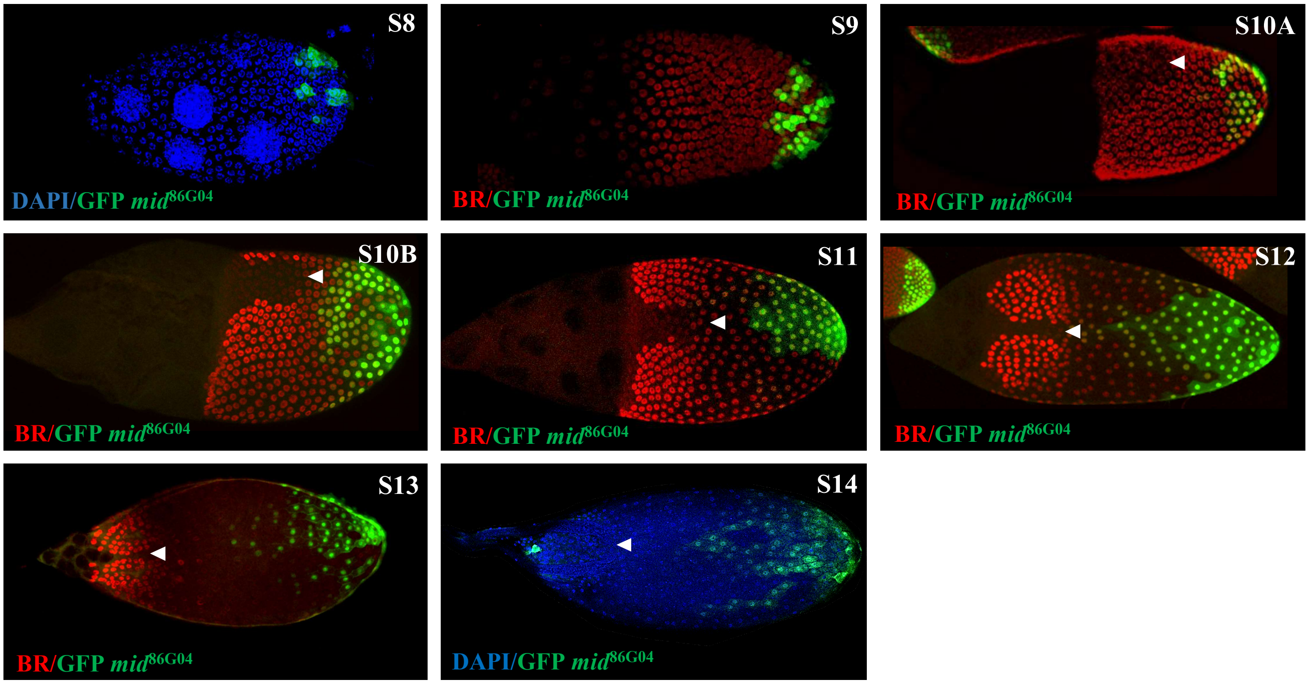

Fig S2 Pa. Gene model for *mid* with RNA-seq coverage plot showing only one isoform expressed during oogenesis. Reads per base pair range between 0-500 for all developmental stages. Fragments mapped below model indicate fly lines screened with highlighted boxes (yellow) representing those that had expression of GFP. **Pb.** Depiction of endogenous *mid* expression in P during S8-10B of oogenesis (Fregoso Lomas et al. 2013). **Pc.** Line *mid*^{86F11}-GAL4 expresses GFP in P domain at S7-14. **Pd.** Line *mid*^{86G04}-GAL4 expresses GFP in P domain at S8-14 and at the BC at S14. Arrowhead denotes the dorsal midline. Anterior is to the left.

Qa. *mirror (mirr)*, CG10601

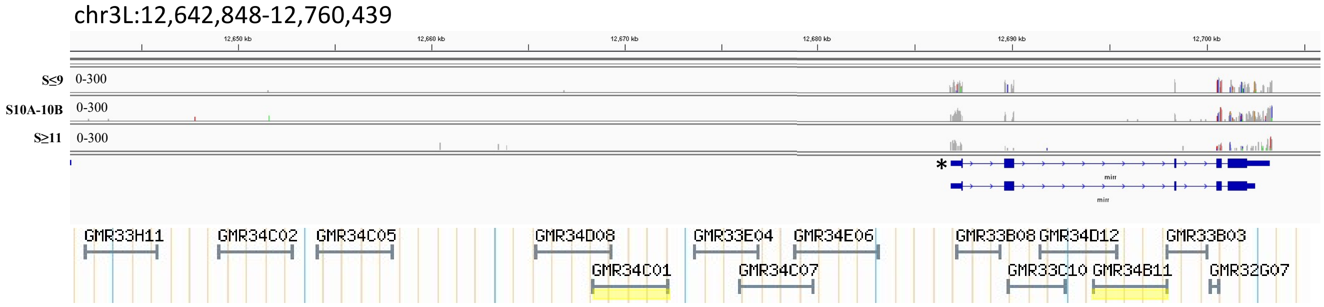

Qb.

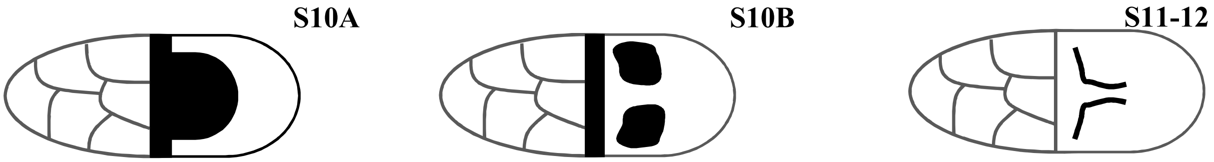

Qc.

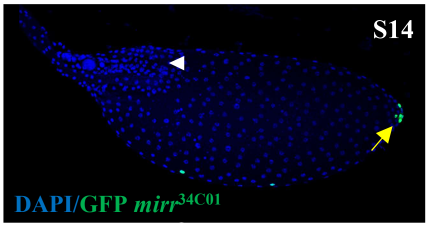

Qd.

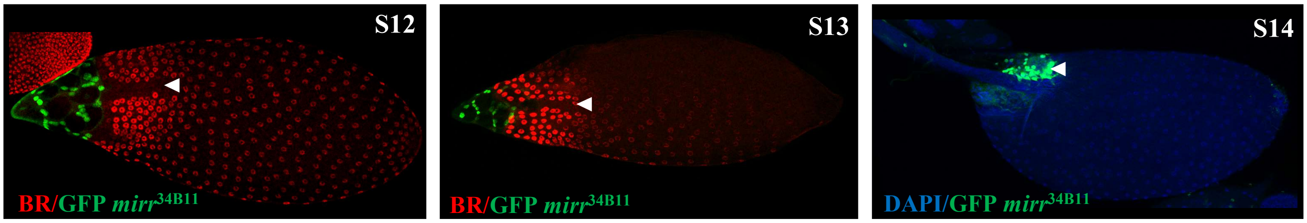

Fig S2 Qa. Gene model for *mirr* with RNA-seq coverage plot showing no discrepancies of the two isoforms expressed during oogenesis. Reads per base pair range between 0-300 for all developmental stages. Lines mapped below model indicate lines that were screened with highlighted boxes representing those that had expression of GFP. **Qb.** Depiction of endogenous *mirr* expression in AD, AV, M, R, and D at S10A, AD, AV, and R at S10B, and F at S11-12 (Jordan et al., 2000). The cartoons are dorsal views, thus the AV domain is not shown. **Qc.** Line *mirr*^{34C01}-GAL4 expressed in P domain at S14. **Qd.** Line *mirr*^{34B11}-GAL4 is expressed in SC at S12-13 and Op at S 14. Arrowhead denotes the dorsal midline. Anterior is to the left.

Ra. *pointed (pnt)*, CG17077

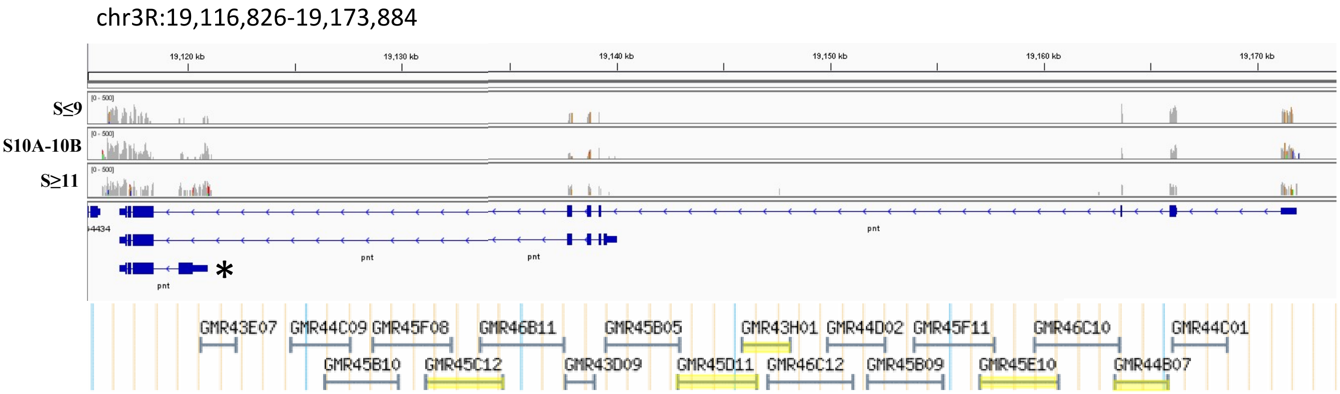

Rb.

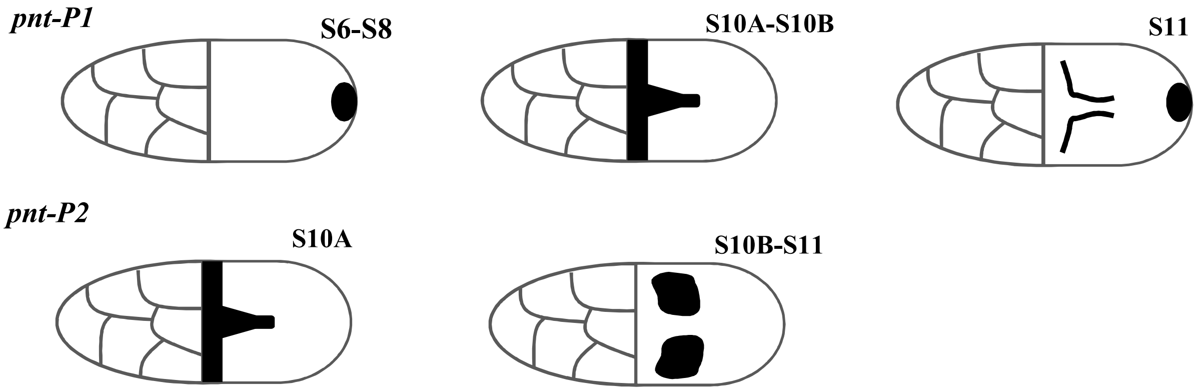

Rc.

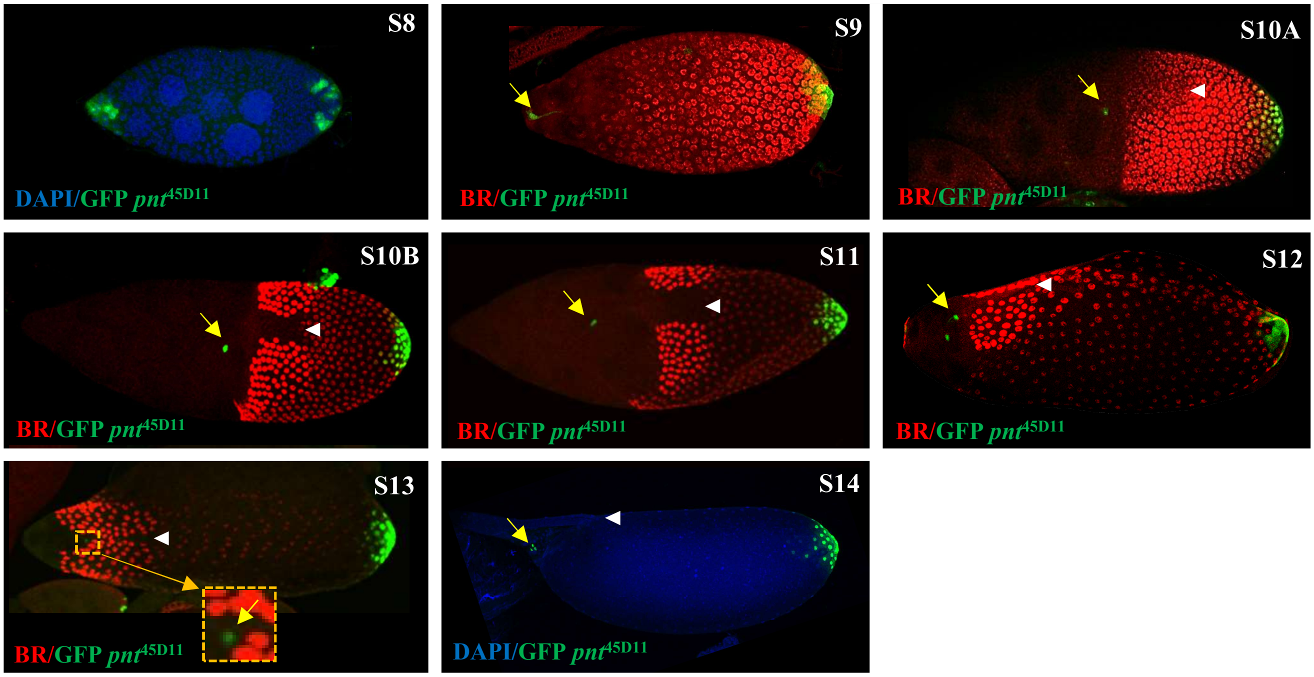

Rd.

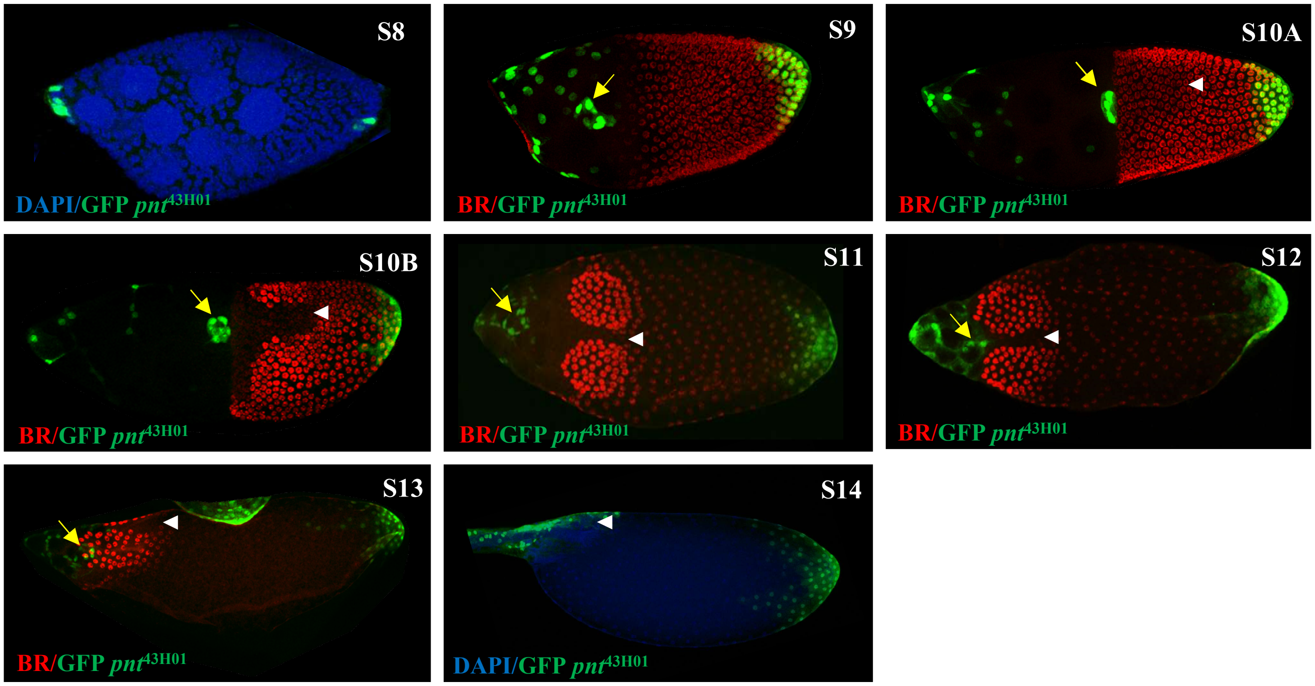

S8
DAPI/GFP pnt43H01
S9
BR/GFP pnt43H01
S10A
BR/GFP pnt43H01
S10B
BR/GFP pnt43H01
S11
BR/GFP pnt43H01
S12
BR/GFP pnt43H01
S13
BR/GFP pnt43H01
S14
DAPI/GFP pnt43H01

Re.

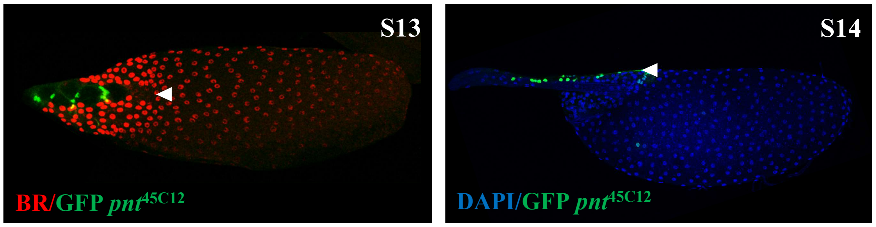

S13
BR/GFP pnt45C12
S14
DAPI/GFP pnt45C12

Rf.

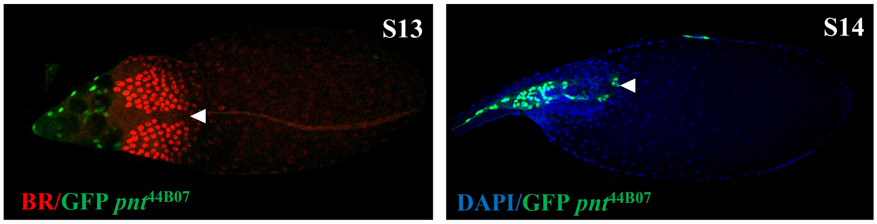

S13
BR/GFP pnt44B07
S14
DAPI/GFP pnt44B07

Rg.

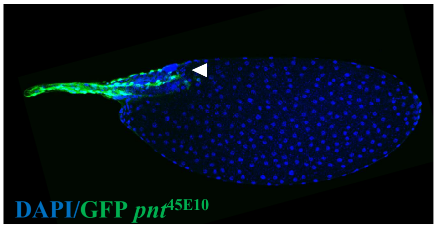

DAPI/GFP pnt45E10

Fig S2 Ra. Gene model for *pnt* with RNA-seq coverage plot showing both *pnt-P1* and *pnt-P2* isoforms active during oogenesis. The isoform, *pnt-P1*, was selected for the mapping analysis as being the only endogenous pattern detected in P during screening (indicated by asterisk*). Reads per base pair range between 0-500 for all developmental stages. Fragments mapped below model indicate lines screened with highlighted boxes (yellow) representing those that had expression of GFP. **Rb.** Depiction of two isoforms, *pnt-P1*(S6-8: P, S10A-10B: AD, AV, and M, S11: F and P), and *Pnt-P2* (S10A: M and AD; S10B-11:R) during oogenesis (Morimoto et al. 1996, Yakoby et al. 2008). **Rc.** Line *pnt*^{45D11}-GAL4 expressed in P, BC (yellow arrow) at S8-14. **Rd.** Line *pnt*^{43H01}-GAL4 expressed in P, BC (yellow arrow), and SC at S8-13, and P and DA at S14. **Re.** Line *pnt*^{45C12}-GAL4 in SC at S13 and DA at S14. **Rf.** Line *pnt*^{44B07}-GAL4 in SC at S13 and DA at S14. **Rg.** Line *pnt*^{45E10}-GAL4 expressed in the DA at S14. Arrowhead denotes the dorsal midline. Anterior is to the left.

Sa. *Ras oncogene at 85D* (*Ras85D*), CG9375

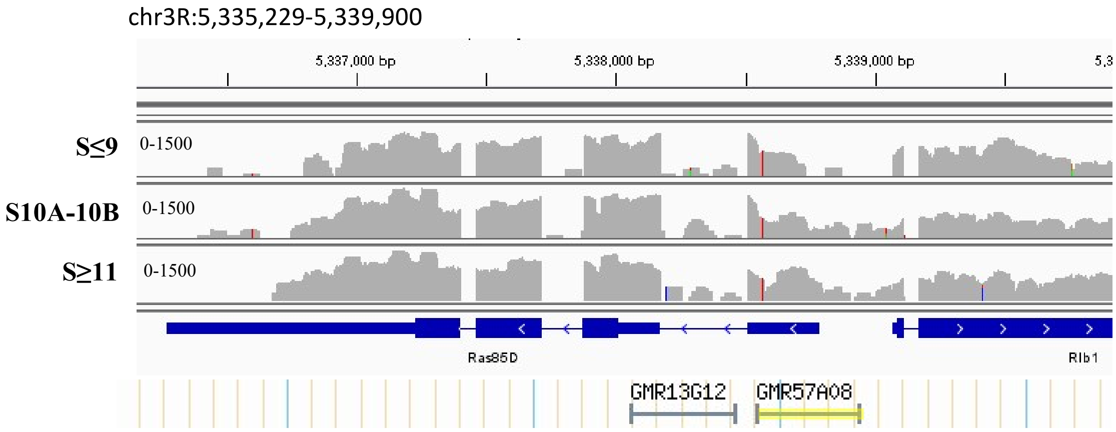

Sb.

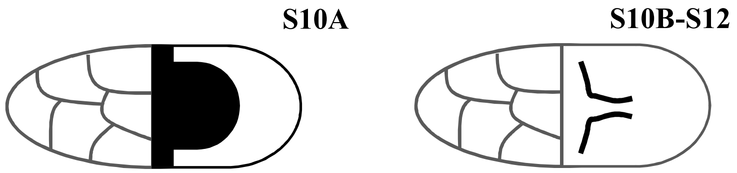

Sc.

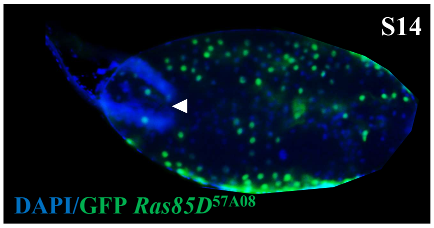

Fig S2 Sa. Gene model for *Ras85D* with RNA-seq coverage plot showing only one predicted isoform is expressed during oogenesis. Reads per base pair range between 0-1500 for all developmental stages. Fragments mapped below model indicate lines screened with highlighted box representing the GFP positive line. **Sb.** Depiction of endogenous *Ras85D* expression in AD, M, R, F, and D at S10A and F at S10B-12 (Yakoby et al. 2008). **Sc.** Stage specific GFP expression of *Ras85D*^{57A08}-GAL4 expressed in the U domain at S14. Images for *Ras85D*^{57A08}-GAL4 were taken using a Leica DM2500 compound microscope. Arrowhead denotes the dorsal midline. Anterior is to the left.

Ta. *rhomboid (rho)*, CG1004

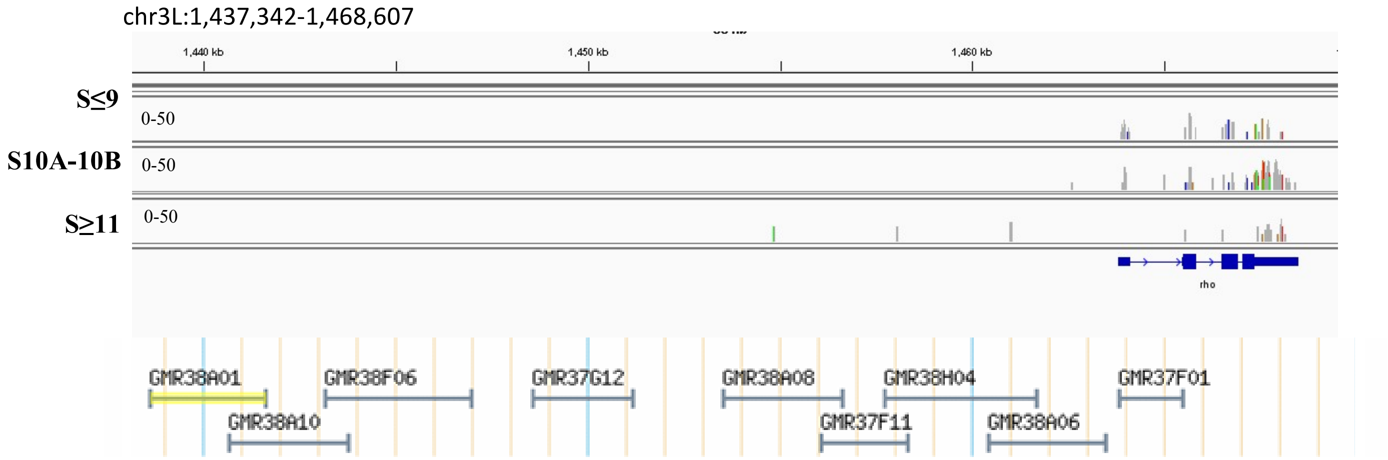

Tb.

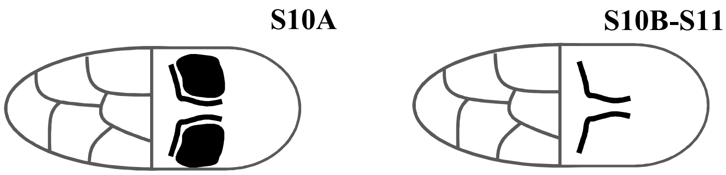

Tc.

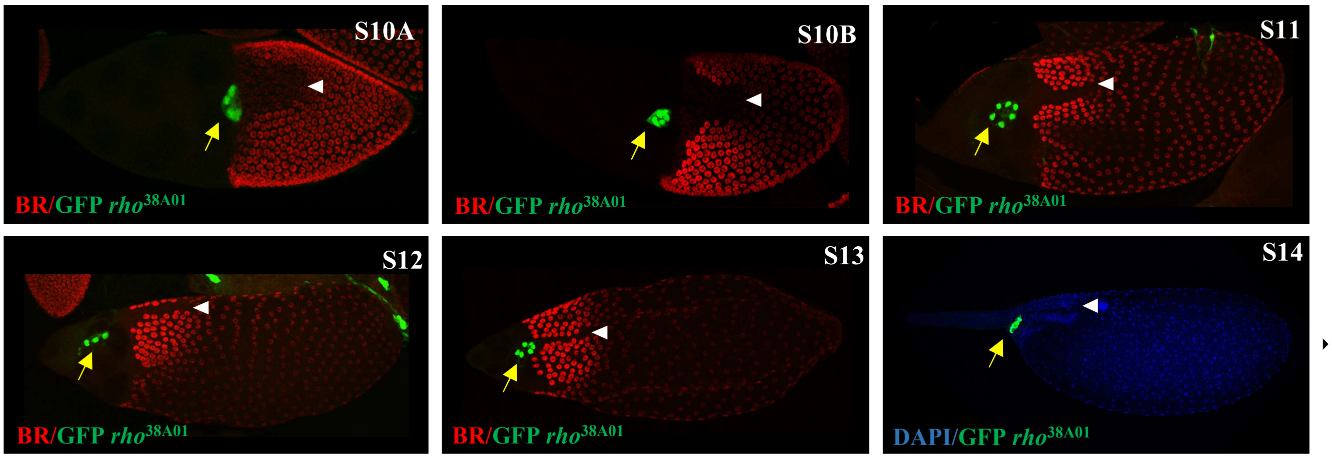

Fig S2 Ta. Gene model for *rho* with RNA-seq coverage plot showing the one predicted isoform is expressed during oogenesis. Fragments mapped below model indicate lines screened with highlighted box representing the GFP positive line. Reads per base pair range between 0-50 for all developmental stages. **Tb.** Depiction of endogenous *rho* expression in F and R at S10A and F at S10B (Ruohola-Baker et al. 1993). **Tc.** Line *rho*^{38A01}-GAL4 expressed in BC (yellow arrows) at S10A-14. Arrowhead denotes the dorsal midline. Anterior is to the left.

Ua. *scarface (scaf)*, CG11066

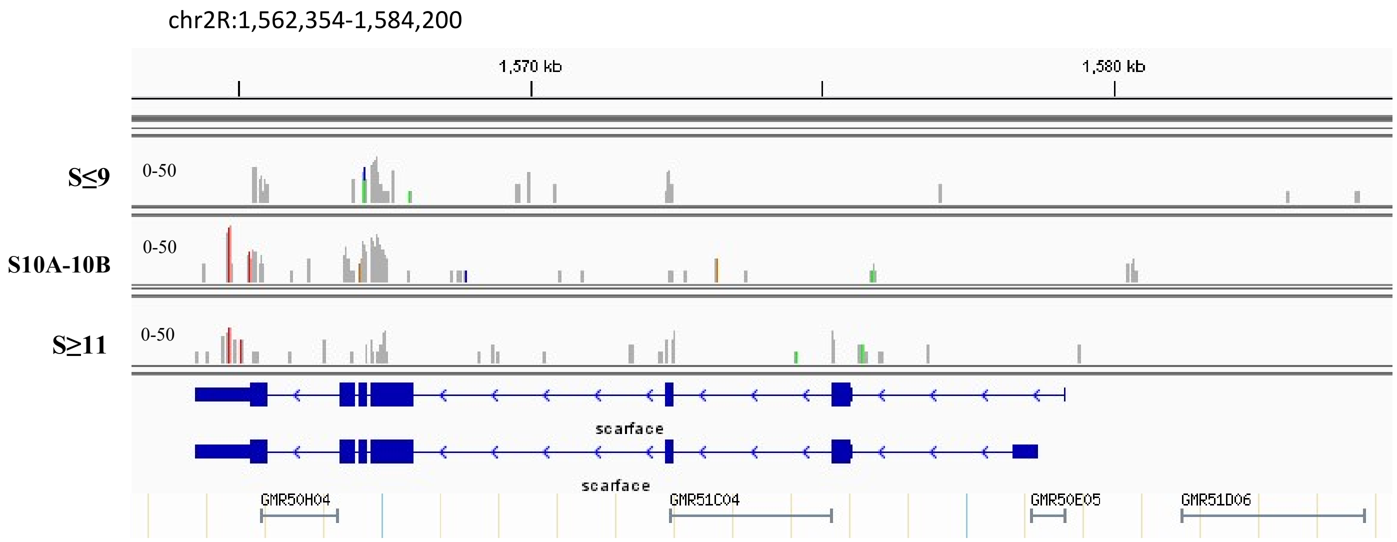

Ub.

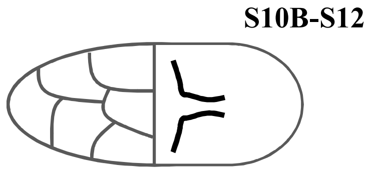

Fig S2 Ua. Gene model for *scaf* with RNA-seq coverage plot showing the active isoform during oogenesis, indicated by asterisk (*). Reads per base pair range between 0- 50 for all developmental stages. Fragments mapped below model indicate lines screened. Reads per base range between 0-50. **Ub.** Depiction of endogenous *scaf* expression in F at S10B-12 (Yakoby et al. 2008). None of the screened lines for *scaf* showed GFP expression.

Va. *shade (shd)*, CG13478

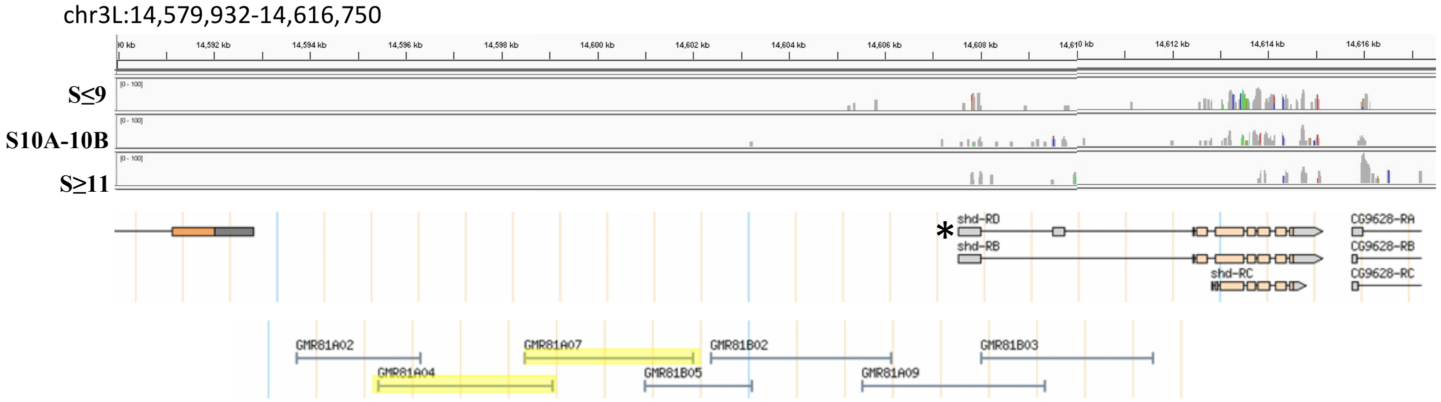

Vb.

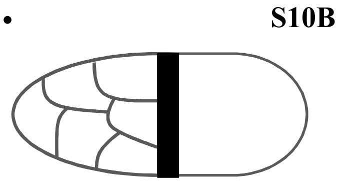

Vc.

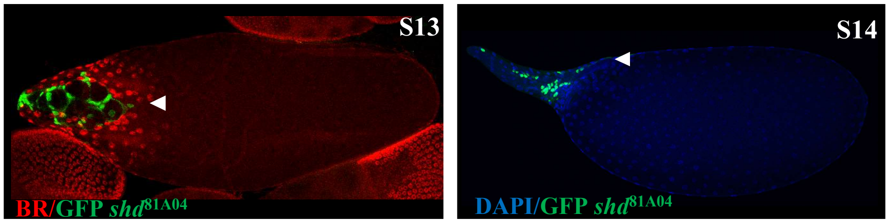

Vd.

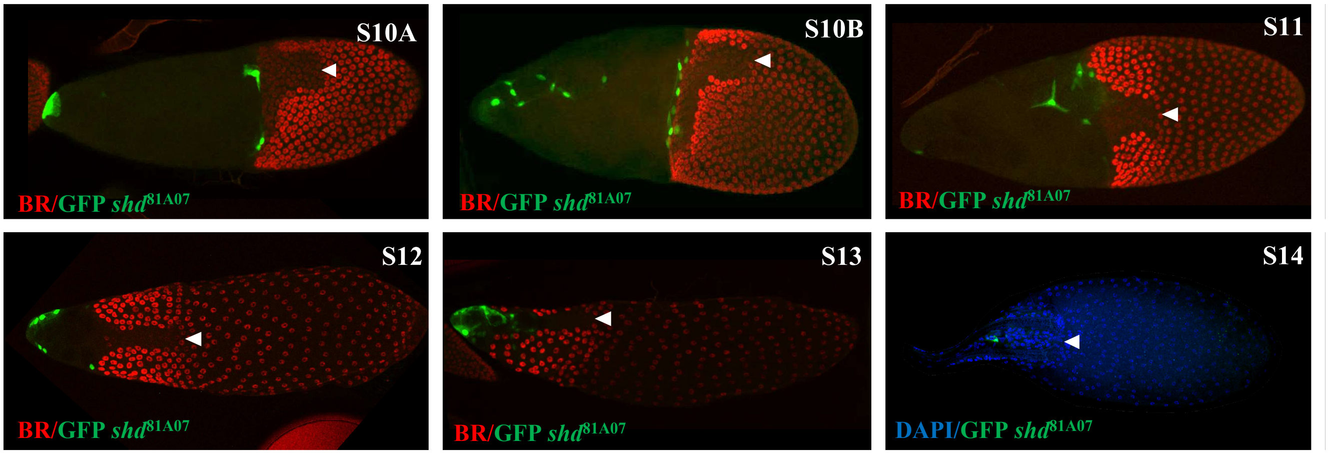

Fig S2 Va. Gene model for *shd* with RNA-seq coverage plot. Additional isoforms for *shd* are available on Flybase.org and aligned to coverage plot, where expression of isoforms RB and RD are shown. Asterisk (*) indicates isoform selected for mapping analysis. Fragments mapped below model indicate lines screened with highlighted boxes (yellow) representing those that had expression of GFP. Reads per base pair range between 0-100 for all developmental stages. Additional lines screened for *shd* are annotated under a neighboring gene, *HGTX* (CG4745). **Vb.** Depiction of endogenous *shd* expression in AD and AV at S10B (Petryk et al. 2003). **Vc.** Line *shd*^{81A04}-GAL4 expressed in SC at S13 and in the DA domain at S14. **Vd.** Line *shd*^{81A07}-GAL4 expressed in SC, AV, and AD domains at S10A-S10B. During S11-S13 it is expressed in the SC, and at S14 in the BC. Arrowhead denotes the dorsal midline. Anterior is to the left.

Bryant, Z., L. Subrahmanyan, M. Tworoger, L. LaTray, C.R. Liu et al., 1999 Characterization of differentially expressed genes in purified Drosophila follicle cells: toward a general strategy for cell type-specific developmental analysis. Proc Natl Acad Sci U S A 96 (10):5559-5564.

Buszczak, M., M.R. Freeman, J.R. Carlson, M. Bender, L. Cooley et al., 1999 Ecdysone response genes govern egg chamber development during mid-oogenesis in Drosophila. Development 126 (20):4581-4589.

Deng, W., K. Leaper, and M. Bownes, 1999 A targeted gene silencing technique shows that Drosophila myosin VI is required for egg chamber and imaginal disc morphogenesis. J Cell Sci 112 (Pt 21):3677-3690.

Deng, W.M., and M. Bownes, 1997 Two signalling pathways specify localised expression of the Broad-Complex in Drosophila eggshell patterning and morphogenesis. Development 124 (22):4639-4647.

Dequier, E., S. Souid, M. Pal, P. Maroy, J.A. Lepesant et al., 2001 Top-DER- and Dpp-dependent requirements for the Drosophila fos/kayak gene in follicular epithelium morphogenesis. Mech Dev 106 (1-2):47-60.

Doerflinger, H., J.A. Lepesant, and C. Yanicostas, 1999 Differential expression of the Drosophila zinc finger gene jim in the follicular epithelium. Mech Dev 86 (1-2):177-182.

Fregoso Lomas, M., F. Hails, J.F. Lachance, and L.A. Nilson, 2013 Response to the dorsal anterior gradient of EGFR signaling in Drosophila oogenesis is prepatterned by earlier posterior EGFR activation. Cell Rep 4 (4):791-802.

Jordan, K.C., N.J. Clegg, J.A. Blasi, A.M. Morimoto, J. Sen et al., 2000 The homeobox gene mirror links EGF signalling to embryonic dorso-ventral axis formation through notch activation. Nat Genet 24 (4):429-433.

Jordan, K.C., S.D. Hatfield, M. Tworoger, E.J. Ward, K.A. Fischer et al., 2005 Genome wide analysis of transcript levels after perturbation of the EGFR pathway in the Drosophila ovary. Dev Dyn 232 (3):709-724.

Morimoto, A.M., K.C. Jordan, K. Tietze, J.S. Britton, E.M. O'Neill et al., 1996 Pointed, an ETS domain transcription factor, negatively regulates the EGF receptor pathway in Drosophila oogenesis. Development 122 (12):3745-3754.

Muzzopappa, M. and Wappner, P. 2005 Multiple roles of the F-box protein Slimb in *Drosophila* egg chamber development. Development 132: 2561-2571

Papadia, S., G. Tzolovsky, D. Zhao, K. Leaper, D. Clyde et al., 2005 emc has a role in dorsal appendage fate formation in Drosophila oogenesis. Mech Dev 122 (9):961-974.

Petryk, A., J.T. Warren, G. Marques, M.P. Jarcho, L.I. Gilbert et al., 2003 Shade is the Drosophila P450 enzyme that mediates the hydroxylation of ecdysone to the steroid insect molting hormone 20-hydroxyecdysone. Proc Natl Acad Sci U S A 100 (24):13773-13778.

Queenan, A.M., A. Ghabrial, and T. Schupbach, 1997 Ectopic activation of torpedo/Egfr, a Drosophila receptor tyrosine kinase, dorsalizes both the eggshell and the embryo. Development 124 (19):3871-3880.

Ruohola-Baker, H., E. Grell, T.B. Chou, D. Baker, L.Y. Jan et al., 1993 Spatially localized rhomboid is required for establishment of the dorsal-ventral axis in Drosophila oogenesis. Cell 73 (5):953-965.

Szafranski, P., and S. Goode, 2004 A Fasciclin 2 morphogenetic switch organizes epithelial cell cluster polarity and motility. Development 131 (9):2023-2036.

Twombly, V., R.K. Blackman, H. Jin, J.M. Graff, R.W. Padgett et al., 1996 The TGF-beta signaling pathway is essential for Drosophila oogenesis. Development 122 (5):1555-1565.

Yakoby, N., C.A. Bristow, D. Gong, X. Schafer, J. Lembong et al., 2008 A combinatorial code for pattern formation in Drosophila oogenesis. Developmental cell 15 (5):725-737.
