# Supplementary material for: Simple Expression Domains Are Regulated by Discrete CRMs During Drosophila Oogenesis
Source: G3 (Bethesda). 2017 Jun 20;7(8):2705–18. doi: 10.1534/g3.117.043810 (PMC5555475; doi:10.1534/g3.117.043810)
Supplement: Supplementary file 3 [file 2705FigureS3.pdf]

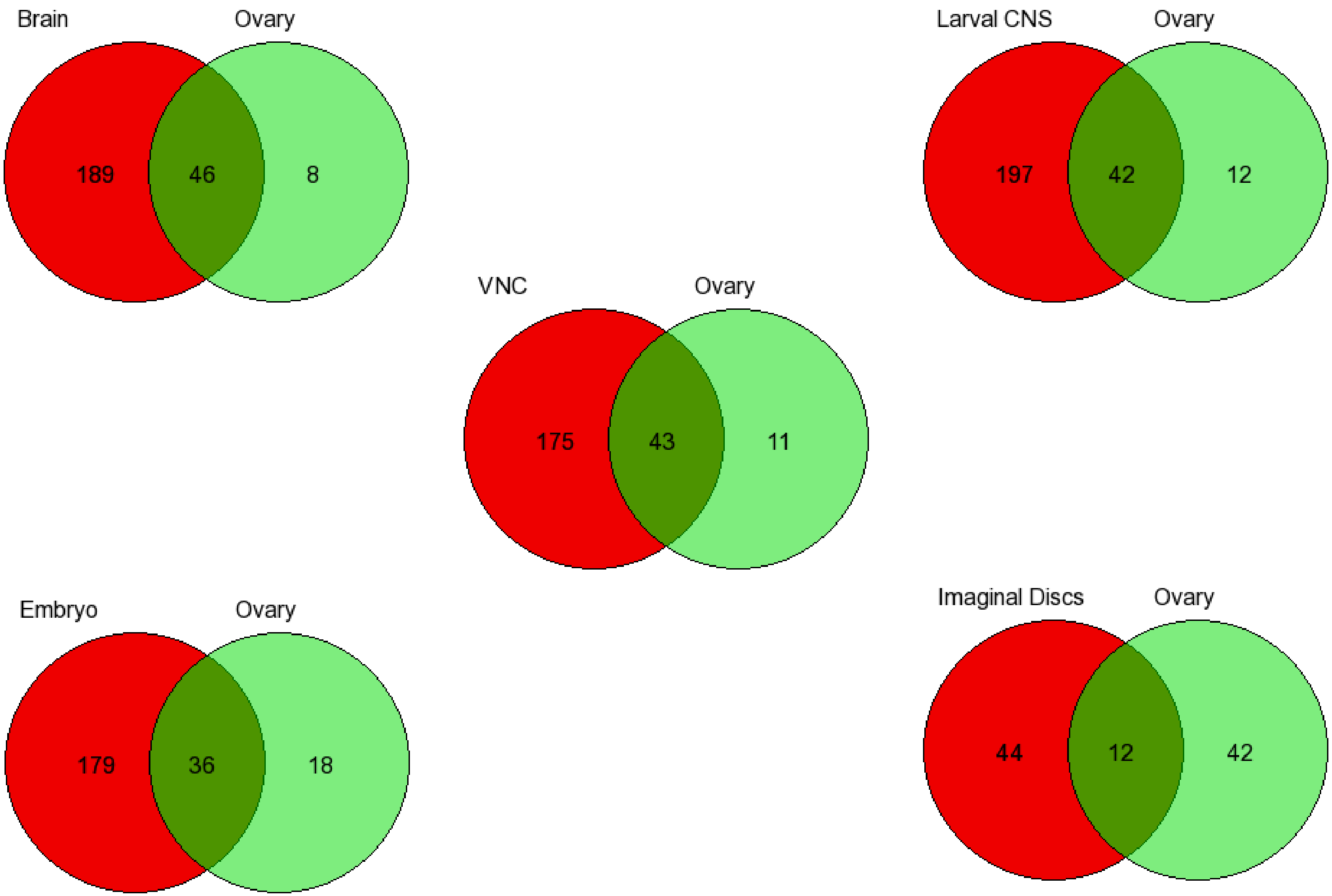

Figure S3: Venn diagram showing the overlap number of lines between the ovary and other tissues. Venn diagrams generated by http://genevenn.sourceforge.net.

Expression patterns for brain, ventral nerve cord (VNC) embryo, larval CNS, and imaginal discs can be found at: http://flweb.janelia.org/cgi-bin/flew.cgi (Jenett et al., 2012,, Manning et al., 2012, Li et al., 2014, Jory et al., 2012)
